# Autologous Extracellular Matrix‐Based Cell‐Free Therapy for Tissue Regeneration Through Trem2^+^ Macrophages Mediated Angiogenesis

**DOI:** 10.1002/EXP.20250031

**Published:** 2026-02-18

**Authors:** Mengmeng Hou, Nini Shi, Yajie Guo, Jiezhang Tang, Han Peng, Baoyan Liang, Yixuan Yu, Chenggang Yi, Huichen Li

**Affiliations:** ^1^ Department of Plastic Surgery Xijing Hospital Fourth Military Medical University Xi'an China; ^2^ Department of Biochemistry and Molecular Biology Fourth Military Medical University Xi'an China; ^3^ Military Medical Innovation Center Fourth Military Medical University Xi'an China; ^4^ Department of Biologic and Materials Sciences University of Michigan Ann Arbor Michigan USA; ^5^ Department of Plastic Surgery and Burns Tangdu Hospital Fourth Military Medical University Xi'an China; ^6^ The Second Affiliated Hospital of Zhejiang University College of Medicine Hangzhou China

**Keywords:** adipose‐derived matrix film, angiogenesis, cell‐free therapy, extracellular matrix, tissue regeneration

## Abstract

The extracellular matrix (ECM) is vital for tissue regeneration and remodeling by providing structural support and regulating cell behavior. Bioactive membranes derived from decellularized ECM show promising regenerative potential in many fields. However, they still face challenges such as immune rejection, structural disparities, and high costs associated with human‐derived materials. These issues hinder their widespread clinical application and limit their adaptability to personalized treatments. Further research is needed to improve the safety, efficacy, and accessibility of ECM‐based materials. This study develops an autologous ECM‐based membrane, termed adipose‐derived matrix film (ADF), using a simple physical method inspired by traditional “*papermaking*.” ADF exhibits favorable biological activity and mechanical strength, essential for tissue regeneration. Its production is efficient, facilitating clinical translation. Additionally, ADF can be stored long‐term at low temperatures, enabling the establishment of an “*ECM bank*” for personalized medicine. As a cell‐free therapy, ADF enhances soft tissue regeneration and wound healing, with Trem2^+^ macrophages playing a key role in neovascularization. We introduce a novel autologous adipose ECM‐derived bio‐membrane, offering new perspectives on ECM preservation and Trem2^+^ macrophage‐mediated regeneration. These findings significantly advance cell‐free regenerative medicine and redefine fundamental mechanisms of tissue repair and vascular restoration.

## Graphical abstract

ADF is prepared as a cell‐free bio‐membrane from autologous adipose ECM using simple physical techniques inspired by “papermaking”. It boasts superior properties and facilitates tissue regeneration through Trem2^+^ macrophages‐mediated angiogenesis. ADF can be stored long‐term for personalized medicine, offering insights into autologous ECM preservation and regeneration.

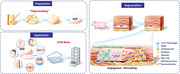

## 1 Introduction

The extracellular matrix (ECM) is a dynamic network of structural and functional proteins that regulates cellular behavior and tissue homeostasis [1]. As both a structural scaffold and biochemical signaling platform, ECM influences critical cellular processes including proliferation, differentiation, and tissue regeneration [2, 3, 4]. Decellularized ECM‐based bio‐membranes have emerged as promising regenerative medicine scaffolds [5, 6]. These bio‐membranes, derived from human skin [7], porcine dermis [8], bovine pericardium [9], and porcine intestinal submucosa [10], exhibit superior biocompatibility and programmable degradation profiles. Their unique properties enable diverse clinical applications, including breast reconstruction, wound healing, periodontal regeneration, and plastic surgery [5, 8]. However, current options (including xenogeneic and allogeneic sources) show clinical potential, limitations persist regarding immune compatibility, structural fidelity, and patient‐specific customization [6, 11]. These challenges highlight the need for improved ECM‐based solutions that better replicate native tissue microenvironments while addressing individual patient requirements [12].

Autologous ECM represents an optimal regenerative material, offering perfect biocompatibility, elimination of rejection risks, and patient‐specific bioactive components. Dermal ECM has been extensively utilized in tissue engineering due to its abundant type I and III collagen content, which provides excellent structural integrity for soft tissue repair [13, 14, 15]. The matrix is particularly valuable for burn treatment [14] and breast reconstruction owing to its rich composition of angiogenic and wound‐healing growth factors [15]. However, clinical applications are limited by donor‐site‐dependent mechanical variability, scarring potential, and insufficient elasticity for dynamic tissue environments [12, 15]. Autologous fascia lata grafts play a vital role in tendon reconstruction, pelvic floor repair, dural closure, stress urinary incontinence treatment, and abdominal wall reconstruction due to their excellent biocompatibility and mechanical strength. However, significant challenges persist, including donor‐site complications (pain and hernia formation), limited graft availability, age‐related tissue quality variability, and prolonged surgical harvesting time—issues that urgently require resolution [16, 17, 18].

Among available autologous ECM options, adipose‐derived ECM represents a uniquely advantageous biological substrate that addresses many limitations of conventional materials [6]. This source combines four critical attributes: (1) high procurement yield with minimal harvest morbidity through liposuction techniques, (2) a porous structural organization amenable to gentle decellularization, (3) a therapeutically rich biomolecular milieu containing high concentrations of adipokines and stem cell factors, and (4) established clinical efficacy across multiple surgical disciplines. The inherent bioactivity of adipose ECM significantly surpasses that of other sources in promoting tissue regeneration and integration, while its exceptional soft tissue compatibility makes it ideal for applications ranging from breast reconstruction to volumetric augmentation [19]. While suboptimal mechanical strength currently represents a notable limitation, this challenge necessitates continued refinement of processing methodologies to enhance structural integrity and improve inter‐batch consistency.

**SCHEME 1:**
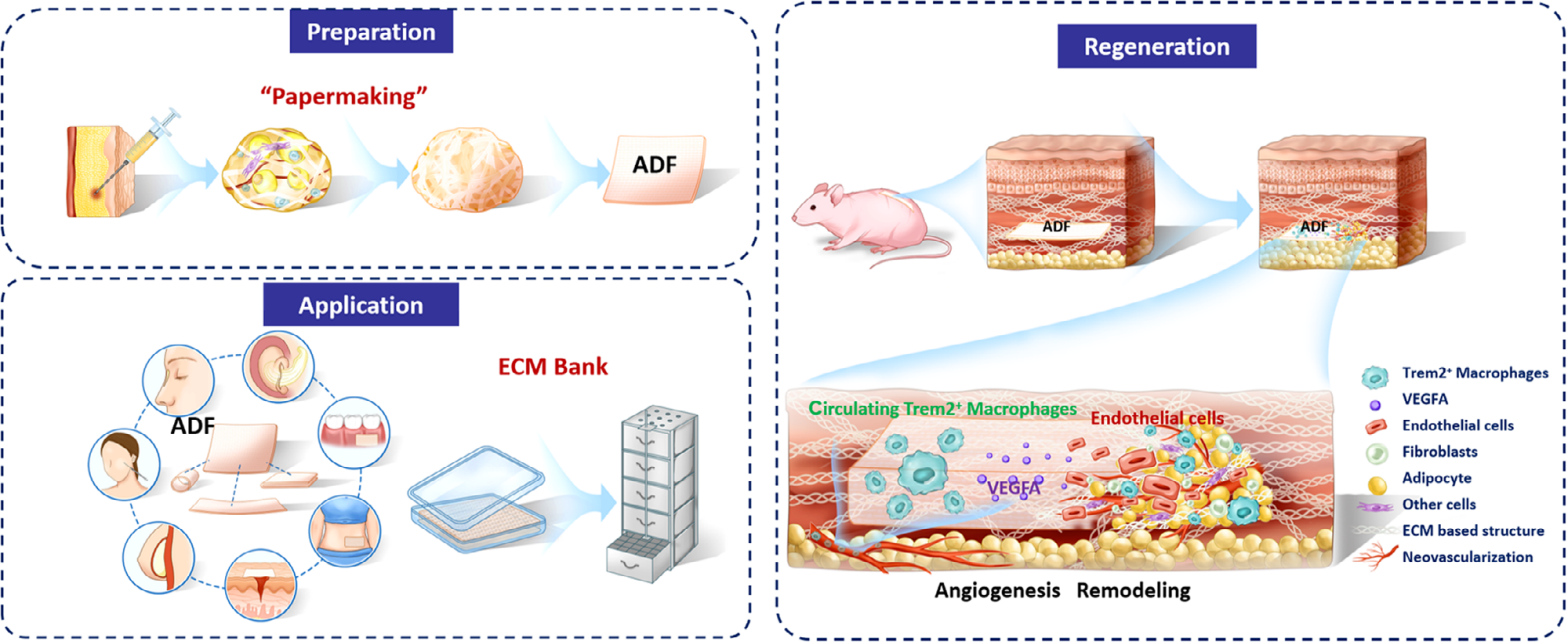
Preparation, regeneration, and application of ADF.

Drawing inspiration from the age‐old art of papermaking, we developed an autologous ECM‐based bio‐membrane from adipose tissue, termed adipose‐derived matrix film (ADF). While current clinical applications of autologous adipose tissue remain largely confined to injectable formulations [20, 21, 22, 23, 24], ADF represents a conceptual and practical advancement in adipose tissue engineering through its unique membrane morphology. The fabrication process employs simple physical techniques enabling efficient lipid and nuclear removal while preserving ECM bioactivity. The resulting ADF exhibits optimal mechanical properties with suitable tensile strength and strain capacity, overcoming the structural limitations of particulate injectable systems. Furthermore, ADF maintains structural integrity and bioactivity after prolonged cryopreservation (frozen ADF, F‐ADF), successfully addressing preservation challenges associated with adipose‐derived biomaterials [20, 21, 22, 23, 24]. Both in vitro and in vivo evaluations confirm ADF's exceptional mechanical properties, biocompatibility, and promotion of vascularization‐mediated soft tissue regeneration. Notably, ADF maintains its therapeutic efficacy in wound healing and tissue regeneration even after exposure to −80°C, demonstrating remarkable stability and clinical translation potential (Scheme 1). This membrane format leverages both the biological properties of native adipose ECM and the practical advantages of membrane‐based delivery systems, enabling previously unattainable clinical applications that require structural support.

Moreover, we observed abundant Trem2^+^ macrophage activation in both tissue regeneration and wound healing processes, suggesting a potential mechanistic link between TREM2 signaling and ADF‐driven vascularization. Single‐cell analyses and our experiments demonstrate that Trem2^+^ macrophages drive angiogenesis through VEGF‐mediated activation of vascular endothelial cells, while fibroblast‐derived FN1 signaling modulates their regenerative function. These findings establish Trem2^+^ macrophages as pivotal mediators of adipose ECM‐induced tissue regeneration, offering new mechanistic insights for regenerative medicine.

## 2 Results

### 2.1 Preparation and Characterization of ADF

**FIGURE 1:**
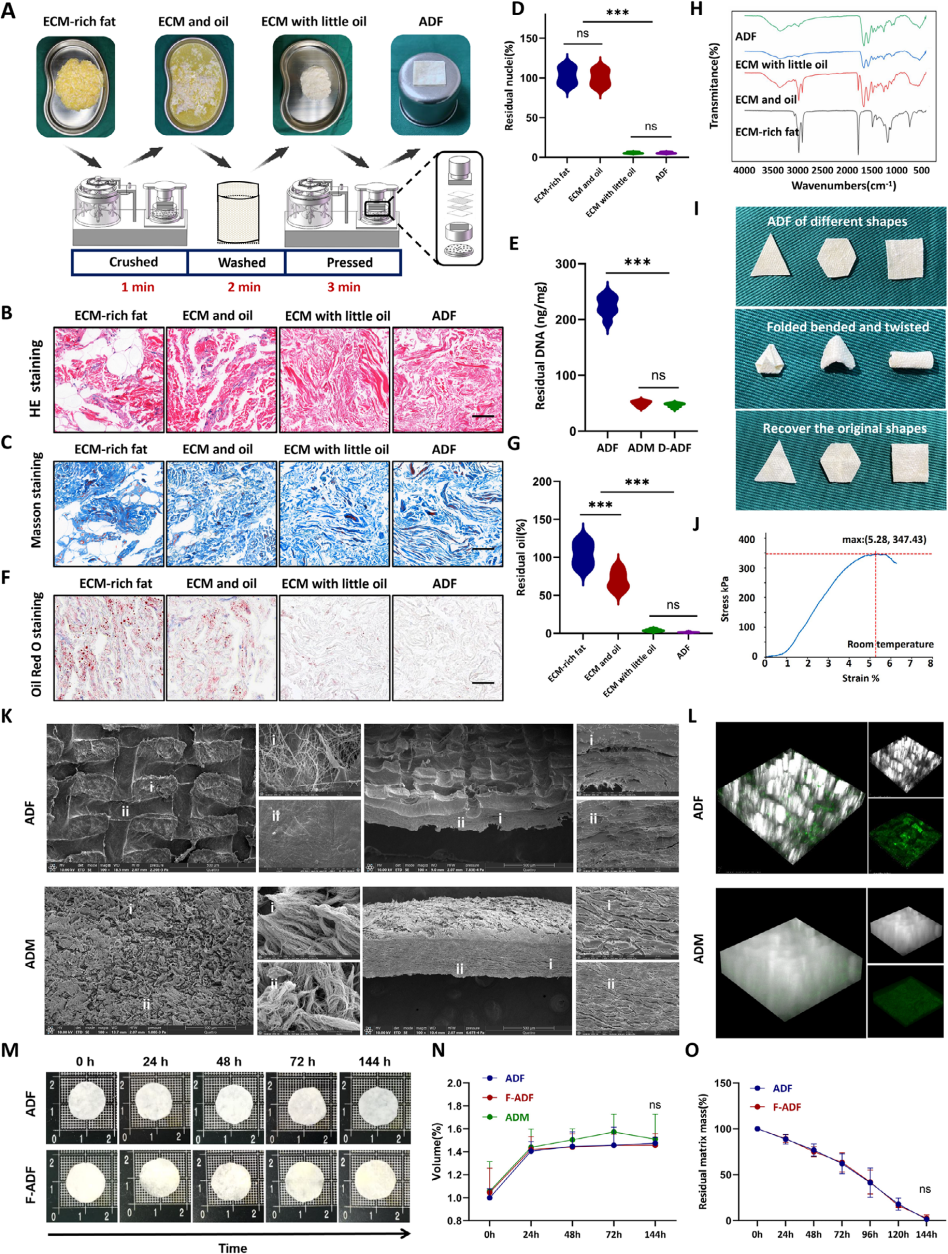
Preparation and characterization of the ADF. (A) The processing procedure of ADF. (B) Hematoxylin and eosin staining and (C) Masson's trichrome staining after different treatments of adipose tissue. Scale bar = 100 µm. (D) Residual nuclei after different treatments of adipose tissue (*n* = 8 per group). (E) Residual DNA of ADF, ADM, and D‐ADF (*n* =8 per group). (F) Oil Red O staining after different treatments of adipose tissue. Scale bar = 100 µm. (G) Residual oil after different treatments of adipose tissue (*n* = 8 per group). (H) FT‐IR spectra of different treatments of adipose tissue. (I) Digital images of the ADF under different operations (bend and twist). (J) Tensile stress–strain of ADF at room temperature. (K) The microstructure of ADF and ADM under scanning electron microscopy (front and side views). (L) 3D reconstruction image of ADF and ADM after laser confocal microscopy scanning (green light shows the surface structure, and white light imaging demonstrates uniform protrusions and depressions across the surface). (M) Images of ADF and F‐ADF immersed in physiological saline at different times. (N) Swelling ratio assessment of ADF, F‐ADF, and ADM immersed in physiological saline at different times (*n* = 8 per group). (O) Degradation rate of ADF and F‐ADF (*n* = 8 per group). *p < 0.05; **p < 0.01; ***p < 0.001; “ns” means non‐significant difference.

The basic approach of “*papermaking*” involves the following steps: (1) obtaining suitable materials rich in fiber; (2) processing to remove impurities; and (3) pressing and drying to form paper. Consistently, the preparation method of ADF is as follows: (1) sourcing autologous adipose tissue (ECM‐rich) via liposuction/abdominal surgery; (2) decellularization via sequential crushing and washing to remove lipids, blood components and cellular debris while preserving native ECM architecture; and (3) mechanical pressing to form bio‐membrane (Figure 1A and Figure S1A,B, Supporting Information). This approach transforms adipose tissue into a cohesive ECM film while maintaining bioactive components essential for regeneration.

To further evaluate the impact of each step of treatment on adipose tissue, we conducted hematoxylin and eosin (HE) staining (Figure 1B), Masson's trichrome staining (Figure 1C), and Oil red O staining (Figure 1F), and made statistical analysis to confirm the removal capability of nuclei (Figure 1D) and oil (Figure 1G). Histological analysis confirmed effective removal of nuclei while preserving ECM integrity (Figure 1B,C). Quantitative assessment revealed only 5.73 ± 0.87% residual nuclei in ADF compared to native tissue, *p* < 0.001 (Figure 1D). The residual DNA content in ADF was analyzed, with commercially acellular dermal matrix (DC‐ADM‐b, Unitrump Bio) serving as a control. The results demonstrated that ADM contained residual DNA at 47.43 ± 4.32 ng/mg (*n* = 8), meeting clinical standards for allogeneic use (Figure 1E). ADF preparation showed significant DNA removal, with residual 224.12 ± 16.91 ng/mg (*n* = 8) (Figure 1E). Although exceeding the clinical standards for allogeneic applications, it may remain acceptable for our primary intended use in rapid autologous applications. To enable potential commercial production and allogeneic applications, we subsequently developed a decellularized adipose derived matrix using established protocols and processed it into film form using our compression method, designated as decellularized adipose derived matrix film (D‐ADF). Further quantitative residual DNA analysis demonstrated that D‐ADF exhibited a residual DNA content of 46.44 ± 3.41 ng/mg (*n* = 8) (Figure 1E), showing no significant difference from ADM (*p* = 0.8310). These findings substantiate that D‐ADF complies with all requisite clinical criteria for allogeneic transplantation, thereby qualifying its suitability for downstream therapeutic product development. The technological progression offers a clinically viable alternative for patients exhibiting inadequate autologous adipose tissue availability. The assessment of α‐Gal antigen in human‐derived allogeneic tissues represents a necessary but insufficient component of pre‐clinical safety evaluation, requiring comprehensive analysis. Therefore, we conducted quantification of α‐Gal via α‐Galactosidase Microplate Assay Kit and nude mice skin as a positive control. The result confirmed undetectable levels in ADF (< 0.1 µg/g, below the threshold for clinical concern) and high levels in positive control (skin of nude mice, 43.84 ± 2.38 µg/g) (Figure S1C, Supporting Information). It further demonstrated its lower risk of immune rejection in clinical applications. Oil Red O staining (Figure 1G) demonstrated effective lipid removal of ADF, with residual lipid significantly reduced to 1.35 ± 0.55% of initial levels (*n* = 8, *p* < 0.001).

Fourier‐transform infrared spectroscopy analysis revealed distinct molecular signatures across each step of treatment on adipose tissue (Figure 1H). The characteristic N–H stretching vibration (amide A band) at 3300 cm^−1^ indicated abundant collagen and elastin content, while the CH_2_ asymmetric and symmetric stretching vibrations at 2920 and 2850 cm^−1^ demonstrated substantial lipid components. A prominent C = O stretching vibration (ester carbonyl) at 1740 cm^−1^ confirmed high triglyceride levels. ECM protein markers were identified at 1650 cm^−1^ (amide I band, collagen α‐helix structure) and 1550 cm^−1^ (amide II band). Additionally, the S = O stretching vibration at 1240 cm^−1^ suggested the presence of sulfated glycosaminoglycans (chondroitin sulfate), and the C─O─C stretching vibration at 1080 cm^−1^ reflected proteoglycan sugar chains. Comparative analysis of the four sample groups showed that both “ECM‐rich fat” and “ECM with oil” groups exhibited significant lipid peaks at 2920/2850 and 1740 cm^−1^, indicating substantial lipid retention. The “ECM with oil” group additionally displayed moderate‐intensity ECM protein peaks, confirming a coexisting state of ECM proteins and lipids. In contrast, the “ECM with little oil” and “ADF” group maintained characteristic ECM protein peaks while showing only minimal residual lipid signals, consistent with parallel histological analysis (Figure 1G).

Tensile testing was conducted on ADF at room temperature, and it exhibited the typical J‐shaped stress–strain curves common in flexible materials (Figure 1J). ADF demonstrated an extensible strength 383.35 ± 16.41 kPa and tensile strain 5.36 ± 0.52% (Figure 1J). Although ADF exhibited slightly reduced mechanical performance compared to commercial ADM, it still displayed favorable mechanical characteristics (Figure S1F, Supporting Information). Under physiological temperature and humidity conditions (equilibrated at 37°C and 95% RH for 2 h), the ADF material exhibited a reduced elastic modulus (383.35 ± 16.41 kPa) while maintaining stable tensile strain (5.34 ± 0.52%) under sustained loading (Figure S1E, Supporting Information). Additionally, humidity exposure induced swelling with a volume increase of 9.32 ± 2.04% (Figure S1H, Supporting Information). ADM also demonstrated a reduction in extensible strength (557.83 ± 21.44 kPa at room temperature and 387.35 ± 19.59 kPa at 37°C, *p* < 0.01) and strain rate (7.34 ± 1.05% at 37°C and 5.88 ± 0.96% at room temperature) (Figure S1F,G, Supporting Information). The results validated that both ADF and ADM maintain mechanical integrity under physiological temperature conditions, confirming their suitability for in vivo applications. ADF retains structural integrity while exhibiting outstanding plasticity and mechanical resilience during curling/folding operations (Figure 1I). Video S1 and Figure S1D, Supporting Information, provided a more intuitive demonstration of its properties.

The surface and cross‐sectional internal structure of ADF was examined with scanning electron microscopy (Figure 1K). The surface of ADF exhibited uniform protrusions and depressions formed by mesh constraint during pressing. It was beneficial for the attachment and contact of the bio‐membrane with tissue, as well as the early infiltration of tissue fluid (Figure 1K). The protruding parts exhibited a more porous scaffold structure conducive to cell infiltration, while the depressed parts are denser. In contrast, ADM lacked such distinctive structural characteristics (Figure 1K). Cross‐sectional observation revealed a dense layered structure of ECM, which was an important reason for its enhanced mechanical strength and properties (Figure 1K). Additionally, small lipid droplets on the ADF's surface indicated the residual oil removal under pressure. It was consistent with previous histological results (Figure 1F,G).

Laser confocal microscopy revealed that compared to ADM, both ADF (Figure 1L and Figure S1J, Supporting Information) and F‐ADF (Figure S1I, Supporting Information) exhibited a rough yet uniform surface morphology under green fluorescence excitation. The white light imaging demonstrated characteristic alternating patterns of thick and thin regions. Subsequently, we assessed the swelling behavior of the ADF, F‐ADF, and ADM. All samples reached swelling equilibrium in physiological saline within 24 h, due to the highly hydrophilic polymer skeleton (Figure 1M and Figure S1K, Supporting Information). The volume changes of ADF, F‐ADF, and ADM over 144 h was shown in Figure 1N. After reaching swelling equilibrium within 24 h, ADM, ADF, and F‐ADF remained unchanged (ADF: 1.36 ± 0.20%; F‐ADF: 1.37 ± 0.18%; and ADM: 1.42 ± 0.21%), with minimal swelling, indicating their swelling resistance (Figure 1N). Consistent with the observed volumetric alterations, ADF, F‐ADF, and ADM (Figure S1L, Supporting Information) exhibited equivalent changes in weight (ADF: 2.22 ± 0.62%; F‐ADF: 2.187 ± 0.65%; and ADM: 2.16 ± 0.59%). This characteristic makes ADF suitable for tissue stabilization and maintaining shape and volume over time after implantation.

We further evaluated the degradation of ADF and F‐ADF under collagenase I treatment by quantifying residual matrix mass at 24 h (ADF: 90.08 ± 5.58%, F‐ADF: 91.79 ± 5.35%), 48 h (ADF: 75.74 ± 13.75%, F‐ADF: 77.64 ± 6.43%), 72 h (ADF: 62.03 ± 10.86 %, F‐ADF: 58.43 ± 13.32%), 96 h (ADF: 39.08 ± 15.64%, F‐ADF: 45.81 ± 17.28%), 120 h (ADF: 15.43 ±7.34%, F‐ADF: 18.81 ± 6.46%), and 144 h (ADF: 1.41 ± 2.18%, F‐ADF: 2.61 ± 3.71%) timepoints (Figure 1O). Cryopreservation does not significantly alter the collagenase susceptibility of ADF, with no significant difference in degradation rates between ADF and F‐ADF throughout the observation period (*p* > 0.05 at all timepoints).

### 2.2 In Vitro Biocompatibility and In Vivo Safety Assessment of ADF

**FIGURE 2:**
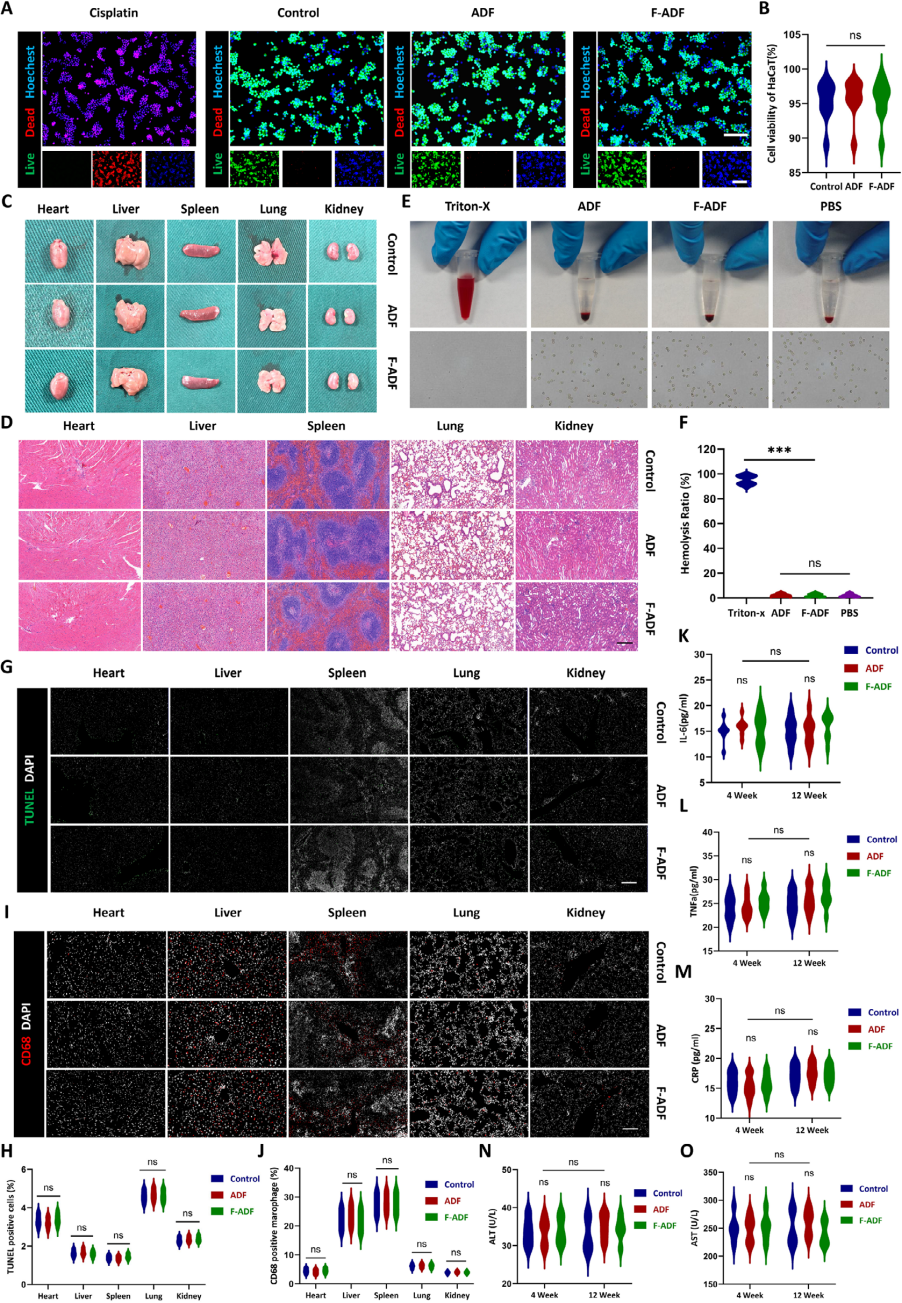
In vitro biocompatibility and in vivo safety assessment of ADF. (A) Representative fluorescence images of calcein‐AM/PI/DAPI staining (green: viable cells; red: nonviable cells; blue: nuclei) in HaCaT after being cultured with ADF extracts for 24 h. (B) Cell viability of HaCaT cells (*n* = 8 per group). (C) Digital images and HE staining (D) of heart, liver, spleen, lung, and kidney tissues at 12 weeks post‐implantation. Scale bar = 100 µm. (E) Digital images of the blood incubated with ADF and F‐ADF for 2 h. (F) Hemolysis ratio of ADF and F‐ADF (*n* = 8 per group). (G) TUNEL staining and (H) quantification of TUNEL‐positive cells. Scale bar = 100 µm. (I) Immunofluorescence staining of CD68 and (J) quantification of CD68‐positive cells. Scale bar = 100 µm. (K) Serum IL‐6, (L) TNF‐α, and (M) CRP levels measured by ELISA at 4 and 12 weeks post‐implantation (*n* = 8 per group). Serum ALT (N) and AST (O) detection at 4 and 12 weeks post‐implantation (*n* = 8 per group). *p < 0.05; **p < 0.01; ***p < 0.001; “ns” means non‐significant difference.

Live/dead cell viability assay was conducted to evaluate the cytotoxicity profiles of ADF and F‐ADF using four clinically relevant cell types: keratinocytes (HaCat cells) (Figure 2A), adipose‐derived stem cells (ADSCs) (Figure S2A, Supporting Information), human umbilical vein endothelial cells (HUVEC) (Figure S2C, Supporting Information), and human dermal fibroblasts (HDF) (Figure S2E, Supporting Information). Following 24 h exposure to ADF/F‐ADF conditioned media (with cisplatin‐treated cells serving as positive controls), calcein‐AM/PI dual staining demonstrated exceptional biocompatibility of both formulations. Quantitative analysis revealed consistently high viability (> 95% calcein‐AM positive cells) and minimal cytotoxicity (< 3% PI‐positive cells) across all cell lineages (Figure 2B and Figure S2B,D,F, Supporting Information), with performance comparable to negative controls and significantly superior to cisplatin‐treated groups (> 95%). Notably, F‐ADF groups showed almost the absence of fluorescence detectable apoptotic or necrotic cells, confirming preserved biocompatibility even after extended storage. These results collectively validate the excellent cytocompatibility of both ADF and F‐ADF formulations for potential clinical applications.

Hemocompatibility, a critical determinant of biomaterial safety, was evaluated through hemolysis assays. As expected, the positive control (Triton‐X 100) induced severe hemolysis, evidenced by a marked transition from colorless to bright red in the supernatant (Figure 2E). In contrast, both ADF and F‐ADF exhibited exceptional blood compatibility, with supernatants maintaining a light yellow hue indistinguishable from the PBS negative control (Figure 2E). Quantitative assessment confirmed that the hemolysis rates for ADF and F‐ADF were below the 5% threshold (ADF: 1.63 ± 1.41% and F‐ADF: 1.88 ± 1.35%) (Figure 2F), complying with ISO 10993‐4: 2017 standards for blood‐contacting materials. These data conclusively demonstrate the hemocompatibility of both formulations, affirming their suitability for clinical applications involving vascularized tissues or direct blood contact.

A comprehensive systemic biocompatibility evaluation was performed through histological and immunohistochemical analyses of major organs (heart, liver, spleen, lung, and kidney) at 12 weeks post‐implantation. Macroscopic photographic evaluation of all organs revealed no macroscopic abnormalities, including organomegaly, necrosis, or edema (Figure 2C). HE staining revealed preserved tissue architecture in all experimental groups (ADF, F‐ADF, and blank control), with no evidence of pathological alterations (Figure 2D). Quantitative assessment demonstrated minimal apoptosis (TUNEL‐positive cells < 5%) across all groups (ADF: 2.69 ± 1.34%, F‐ADF: 2.69 ± 1.12%, and control: 2.75 ± 1.08%, one‐way ANOVA with Tukey's post hoc test, *p* = 0.7740) (Figure 2G,H). Physiological CD68^+^ macrophage infiltration was observed at baseline levels (< 5% in heart, kidney, and lung) with moderately higher distribution in liver (23.47 ± 3.59%) and spleen (27.97 ± 3.37%) (reflecting native immune surveillance functions), showing no significant intergroup differences (*p* = 0.8523) (Figure 2I,J). These findings were consistent across all analyzed specimens (five organs per animal; *n* = 8 per group) evaluated through double‐blind examination by two independent pathologists. The collective data confirm the absence of systemic toxicity, negligible inflammatory responses, and biocompatibility.

We also performed integrated hematological, biochemical, and immunological analyses. Hematological evaluation demonstrated physiological maintenance of all cellular components, with erythrocyte (week 4: 9.05 ± 0.88 × 10^12^/L and week 12: 8.98 ± 0.93 × 10^12^/L in ADF) and leukocyte (week 4: 1.72 ± 0.14 × 10^9^/L and week 12: 1.76 ± 0.23 × 10^9^/L in ADF) (Figure S2G,H, Supporting Information). It showed no statistically significant differences compared to sham controls and F‐ADF group (*p* > 0.05 for all parameters). Complete blood count data are presented in Tables 1 and 2. Hepatic function remained stable in both ADF and F‐ADF groups, with transaminase levels (ALT: 28.15–39.00 U/L and AST: 219.85–290.23 U/L) consistently within normal ranges at 4 and 12 weeks post‐implantation, showing no significant difference versus sham controls (Figure 2N,O). Renal function evaluation demonstrated physiological levels of nitrogen metabolism markers (BUN: 5.20–6.51 mmol/L and CRE: 9.02–11.50 µmol/L) in all experimental groups, with values equivalent to sham controls throughout the observation period (Figure S2I,J, Supporting Information). Comprehensive serum biochemistry data are provided in Tables 3 and 4.

**TABLE 1:** Blood routine examination of 4 weeks.

	Control	ADF	F‐ADF
WBC (10^9^/L)	1.79 ± 0.27	1.72 ± 0.21	1.81 ± 0.12
Neu (10^9^/L)	0.94 ± 0.19	0.91 ± 0.09	0.96 ± 0.21
Lym (10^9^/L)	0.77 ± 0.15	0.68 ± 0.13	0.72 ± 0.51
Mon (10^9^/L)	0.05 ± 0.02	0.05 ± 0.06	0.04 ± 0.03
Eos (10^9^/L)	0.04 ± 0.02	0.04 ± 0.01	0.03 ± 0.09
Bas (10^9^/L)	0 ± 0	0 ± 0	0 ± 0
Neu (%)	52.13 ± 4.96	51.32 ± 4.89	52.4 5 ± 4.13
Lym (%)	42.43 ± 4.6	42.47 ± 4.7	42.75 ± 4.1
Mon (%)	3.09 ± 1.4	3.05 ± 1.2	3.12 ± 1.1
Eos (%)	2.21 ± 1.04	2.25 ± 1.03	2.19 ± 1.45
Bas (%)	0.14 ± 0.11	0.14 ± 0.09	0.16 ± 0.05
RBC (10^12^/L)	8.23 ± 0.73	8.14 ± 0.53	8.27 ± 0.89
HGB (g/L)	132.4 ± 11.76	133.7 ± 11.35	132.9 ± 11.24
HCT (%)	41.32 ± 3.5	41.57 ± 3.1	41.07 ± 3.9
MCV (fL)	50.27 ± 0.98	50.34 ± 0.78	50.51 ± 0.14
MCH (pg)	16.08 ± 0.43	16.13 ± 0.03	16.75 ± 0.41
MCHC (g/L)	320.4 ± 4.25	321.4 ± 4.22	320.1 ± 4.97
RDW‐CV (%)	13.45 ± 0.54	13.51 ± 0.49	13.37 ± 0.76
RDW‐SD (fL)	28.01 ± 0.93	27.03 ± 0.87	28.12 ± 0.33
PLT (10^9^/L)	608.5 ± 82.91	607.5 ± 82.35	610.5 ± 82.11
MPV (fL)	5.65 ± 0.24	5.61 ± 0.56	5.57 ± 0.13
PDW	15.34 ± 0.21	15.37 ± 0.46	15.17 ± 0.88
PCT (%)	0.34 ± 0.05	0.31 ± 0.08	0.34 ± 0.14

Abbreviations: Bas, basophil count; Bas, basophil percentage; Eos, eosinophil count; Eos, eosinophil percentage; HCT, hematocrit; HGB, hemoglobin; Lym, lymphocyte count; Lym, lymphocyte percentage; MCH, mean corpuscular hemoglobin; MCHC, mean corpuscular hemoglobin concentration; MCV, mean corpuscular volume; Mon, monocyte count; Mon, monocyte percentage; MPV, mean platelet volume; Neu, neutrophil count; Neu, neutrophil percentage; PCT, plateletcrit; PDW, platelet distribution width; PLT, platelet count; RBC, red blood cell count; RDW‐CV, red cell distribution width (coefficient of variation); RDW‐SD, red cell distribution width (standard deviation); WBC, white blood cell count.

**TABLE 2:** Blood routine examination of 12 weeks.

	Control	ADF	F‐ADF
WBC (10^9^/L)	1.82 ± 0.26	1.71 ± 0.22	1.80 ± 0.13
Neu (10^9^/L)	0.93 ± 0.18	0.92 ± 0.08	0.95 ± 0.20
Lym (10^9^/L)	0.76 ± 0.16	0.69 ± 0.14	0.71 ± 0.50
Mon (10^9^/L)	0.06 ± 0.03	0.04 ± 0.05	0.05 ± 0.04
Eos (10^9^/L)	0.03 ± 0.03	0.05 ± 0.02	0.04 ± 0.08
Bas (10^9^/L)	0 ± 0	0 ± 0	0 ± 0
Neu (%)	52.20 ± 4.95	51.30 ± 4.90	52.50 ± 4.12
Lym (%)	42.40 ± 4.59	42.50 ± 4.69	42.80 ± 4.09
Mon (%)	3.10 ± 1.39	3.04 ± 1.21	3.11 ± 1.11
Eos (%)	2.20 ± 1.05	2.26 ± 1.02	2.18 ± 1.44
Bas (%)	0.15 ± 0.10	0.13 ± 0.08	0.15 ± 0.06
RBC (10^12^/L)	8.22 ± 0.74	8.15 ± 0.52	8.26 ± 0.88
HGB (g/L)	132.3 ± 11.75	133.8 ± 11.34	133.0 ± 11.23
HCT (%)	41.30 ± 3.51	41.60 ± 3.09	41.10 ± 3.89
MCV (fL)	50.26 ± 0.97	50.35 ± 0.77	50.50 ± 0.15
MCH (pg)	16.09 ± 0.42	16.12 ± 0.04	16.74 ± 0.40
MCHC (g/L)	320.3 ± 4.26	321.5 ± 4.21	320.2 ± 4.96
RDW‐CV (%)	13.44 ± 0.55	13.50 ± 0.48	13.38 ± 0.75
RDW‐SD (fL)	28.00 ± 0.94	27.02 ± 0.86	28.11 ± 0.34
PLT (10^9^/L)	608.6 ± 82.90	607.4 ± 82.34	610.4 ± 82.10
MPV (fL)	5.64 ± 0.25	5.62 ± 0.55	5.58 ± 0.14
PDW	15.33 ± 0.22	15.38 ± 0.45	15.18 ± 0.87
PCT (%)	0.35 ± 0.06	0.32 ± 0.07	0.33 ± 0.13

Abbreviations: Bas, basophil count; Bas, basophil percentage; Eos, eosinophil count; Eos, eosinophil percentage; HCT, hematocrit; HGB, hemoglobin; Lym, lymphocyte count; Lym, lymphocyte percentage; MCH, mean corpuscular hemoglobin; MCHC, mean corpuscular hemoglobin concentration; MCV, mean corpuscular volume; Mon, monocyte count; Mon, monocyte percentage; MPV, mean platelet volume; Neu, neutrophil count; Neu, neutrophil percentage; PCT, plateletcrit; PDW, platelet distribution width; PLT, platelet count; RBC, red blood cell count; RDW‐CV, red cell distribution width (coefficient of variation); RDW‐SD, red cell distribution width (standard deviation); WBC, white blood cell count.

**TABLE 3:** Blood biochemical analysis of 4 weeks.

	Control	ADF	F‐ADF
ALT (U/L)	31.22 ± 9.43	30.98 ± 9.12	31.43 ± 8.98
AST (U/L)	240.86 ± 132.25	241.57 ± 130.14	239.98 ± 131.45
TG (mmol/L)	1.37 ± 0.41	1.34 ± 0.42	1.35 ± 0.34
TC (mmol/L)	2.58 ± 0.42	2.56 ± 0.34	2.61 ± 0.25
Glu‐G (mmol/L)	4.66 ± 1.29	4.36 ± 1.31	4.27 ± 1.16
ALP (U/L)	193.81 ± 25.21	190.65 ± 25.21	189.81 ± 24.76
γ‐GT (U/L)	1.46 ± 0.48	1.37 ± 0.52	1.49 ± 0.21
CREA (µmol/L)	10.86 ± 1.56	10.23 ± 0.52	11.02 ± 0.11
TP (g/L)	55.38 ± 2.53	54.25 ± 2.33	55.22 ± 2.73
α‐AMY (U/L)	3136.44 ± 925.96	3142.44 ± 923.94	3125.41 ± 947.36
CK (U/L)	2364.61 ± 1613.78	2378.16 ± 1613.32	2367.54 ± 1611.24
LDH (U/L)	1027.5 ± 172.53	1021.5 ± 174.36	1030.5 ± 171.61
ALB II (g/L)	31.12 ± 1.34	30.21 ± 1.45	31.56 ± 1.31
LIP (U/L)	32.41 ± 5.73	33.15 ± 5.71	32.56 ± 5.32
UREA (mmol/L)	8.65 ± 1.1	8.63 ± 1.0	8.72 ± 1.4
Ca (mmol/L)	2.25 ± 0.07	2.31 ± 0.04	2.28 ± 0.13
P (mmol/L)	2.44 ± 0.2	2.56 ± 0.3	2.47 ± 0.1
Mg II (mmol/L)	1.47 ± 0.08	1.46 ± 0.03	1.51 ± 0.11
BUN (mmol/L)	5.07 ± 0.37	5.17 ± 0.32	5.64 ± 0.72

Abbreviations: ALB, albumin; ALP, alkaline phosphatase; ALT, alanine aminotransferase; AST, aspartate aminotransferase; BUN, blood urea nitrogen; Ca, calcium; Glu‐G, glucosel; CK, creatine kinase; LDH, lactate dehydrogenase; LIP, lipase; Mg, magnesium; P, phosphorus; TC, total cholesterol; TG, triglycerides; TP, total protein; CREA, creatinine; UREA, urea; α‐AMY, alpha‐amylase; γ‐GT, gamma‐glutamyl transferase.

**TABLE 4:** Blood biochemical analysis of 12 weeks.

	Control	ADF	F‐ADF
ALT (U/L)	31.20 ± 9.42	30.99 ± 9.11	31.43 ± 9.17
AST (U/L)	240.90 ± 132.20	241.55 ± 130.16	239.99 ± 131.41
TG (mmol/L)	1.38 ± 0.40	1.35 ± 0.41	1.36 ± 0.33
TC (mmol/L)	2.59 ± 0.41	2.57 ± 0.33	2.60 ± 0.26
Glu‐G (mmol/L)	4.67 ± 1.28	4.35 ± 1.30	4.28 ± 1.15
ALP (U/L)	193.80 ± 25.20	190.64 ± 25.20	189.82 ± 24.75
γ‐GT (U/L)	1.47 ± 0.47	1.38 ± 0.51	1.50 ± 0.20
CREA (µmol/L)	10.85 ± 1.55	10.24 ± 0.51	11.03 ± 0.12
TP (g/L)	55.37 ± 2.52	54.26 ± 2.32	55.23 ± 2.72
α‐AMY (U/L)	3136.40 ± 925.95	3142.45 ± 923.93	3125.40 ± 947.35
CK (U/L)	2364.60 ± 1613.77	2378.15 ± 1613.31	2367.55 ± 1611.23
LDH (U/L)	1027.4 ± 172.52	1021.4 ± 174.35	1030.6 ± 171.60
ALB II (g/L)	31.11 ± 1.33	30.20 ± 1.44	31.55 ± 1.30
LIP (U/L)	32.40 ± 5.72	33.14 ± 5.70	32.55 ± 5.31
UREA (mmol/L)	8.66 ± 1.09	8.64 ± 1.01	8.73 ± 1.39
Ca (mmol/L)	2.26 ± 0.08	2.32 ± 0.05	2.27 ± 0.14
P (mmol/L)	2.45 ± 0.19	2.55 ± 0.29	2.48 ± 0.11
Mg II (mmol/L)	1.48 ± 0.09	1.45 ± 0.04	1.52 ± 0.10
BUN (mmol/L)	5.08 ± 0.36	5.18 ± 0.31	5.63 ± 0.71

Abbreviations: ALB: albumin; ALP, alkaline phosphatase; ALT, alanine aminotransferase; AST, aspartate aminotransferase; BUN, blood urea nitrogen; Ca, calcium; CK, creatine kinase; Glu‐G, glucose; LDH, lactate dehydrogenase; LIP, lipase; Mg: magnesium; P, phosphorus; TC, total cholesterol; TG, triglycerides; TP, total protein; CREA, creatinine; UREA, urea; α‐AMY, alpha‐amylase; γ‐GT, gamma‐glutamyl transferase.

ELISA analysis showed baseline levels of pro‐inflammatory cytokines (IL‐6: 15.89 ± 1.62 pg/mL, TNF‐α: 24.37 ± 2.25 pg/mL, and CRP: 15.39 ± 1.89 pg/mL) in the ADF group, comparable to sham controls and the F‐ADF group, indicating no systemic inflammatory response (Figure 2K–M). Collectively, these multilayered analyses, encompassing cellular, metabolic, and immunological parameters, provide robust evidence for the hemocompatibility and systemic biocompatibility of both ADF and F‐ADF implants, supporting their translational potential for clinical applications.

Comprehensive safety evaluation established the excellent biocompatibility of both ADF and F‐ADF, demonstrating > 95% cell viability across multiple cell lines (indicating negligible cytotoxicity), < 5% hemolysis (per ISO 10993‐4 standards), and preserved material stability after 12‐month cryopreservation. Histopathological and serum biochemical analyses revealed normal organ function markers (ALT, AST, BUN, and CRE) and baseline‐level inflammatory cytokines (IL‐6, TNF‐α, and CRP), with no evidence of systemic toxicity or significant immune activation compared to sham controls. These findings collectively validate the clinical‐grade safety profile of these materials.

### 2.3 Functional Regulation of ADF on Adipose‐Derived Mesenchymal Stem Cells, Vascular Endothelial Cells, Keratinocytes, and Fibroblasts

The ECM is rich in bioactive components that critically regulate cellular behavior [25, 26, 27, 28, 29]. To evaluate the biological effects of ADF, we established an in vitro co‐culture model to assess the functional modulation. ADF demonstrates a pronounced ability to promote adipose tissue regeneration due to its tissue‐specific extracellular matrix composition derived from native adipose tissue [30]. ADF and F‐ADF effects on ADSCs were assessed for proliferation, migration, and adipogenic potential.

**FIGURE 3:**
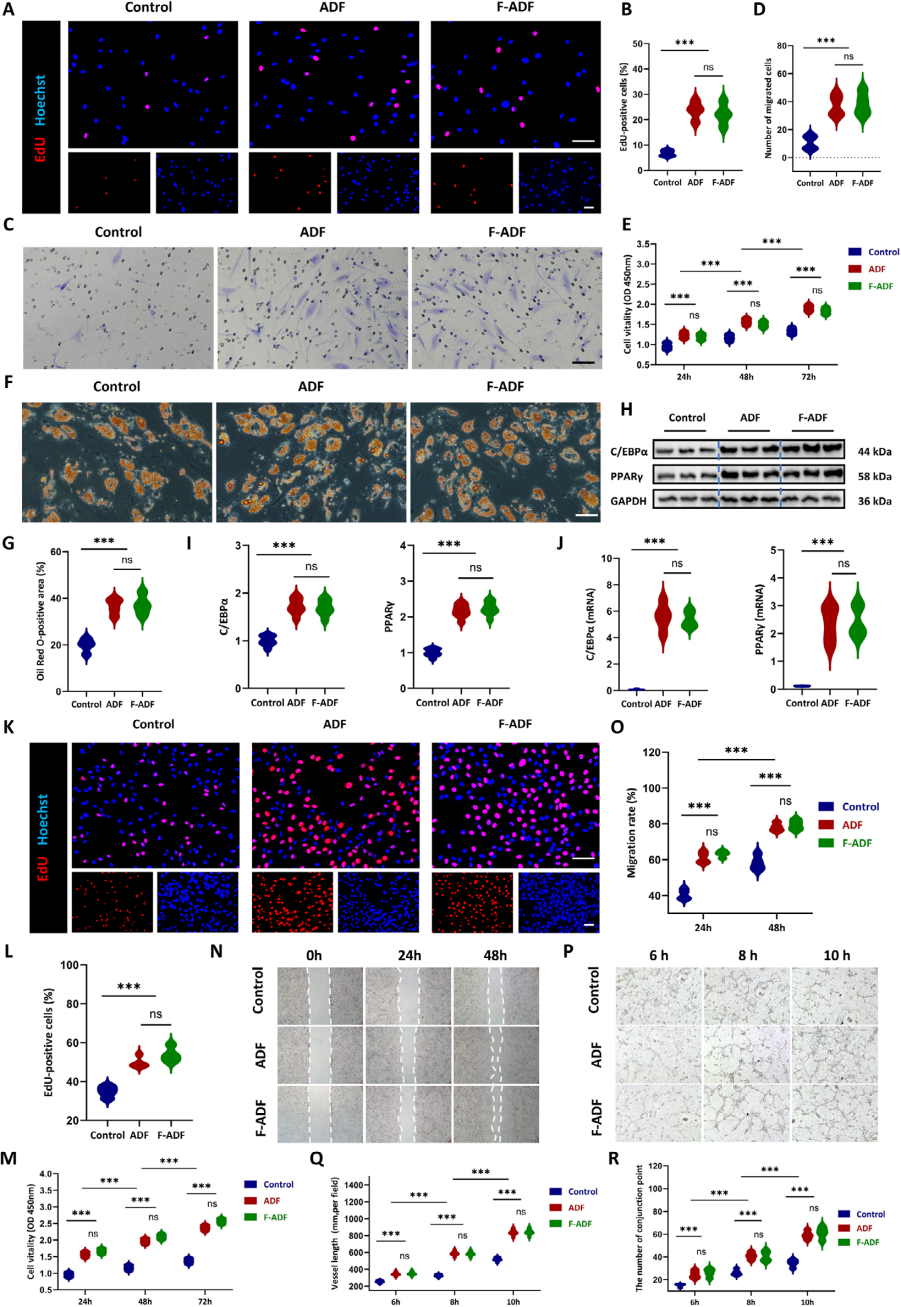
Functional regulation of ADF on ADSCs and HUVEC. (A,B) Cell proliferation of ADSCs after being cultured with ADF and F‐ADF extracts for 24 h with EdU assay (*n* = 8 per group). Scale bar = 100 µm. (C,D) Cell migration of ADSCs after being cultured with ADF and F‐ADF extracts for 24 h with Transwell assay (*n* = 8 per group). Scale bar = 100 µm. (E) CCK‐8 assay of ADSCs after being cultured with ADF and F‐ADF extracts for 24, 48, and 72 h (*n* = 8 per group). (F,G) Oil Red O staining of ADSCs after being cultured with ADF and F‐ADF extracts for 21 days (*n* = 8 per group). Scale bar = 100 µm. (H,I) Western blot analysis of C/EBPα and PPAR γ (*n* = 8 per group). (J) qPCR analysis of C/EBPα and PPAR γ (*n* = 8 per group). (K,L) Cell proliferation of HUVEC after being cultured with ADF and F‐ADF extracts for 24 h with EdU assay (*n* = 8 per group). Scale bar = 100 µm. (M) CCK‐8 assay of HUVEC after being cultured with ADF and F‐ADF extracts for 24, 48, and 72 h (*n* = 8 per group). (N,O) Cell migration of HUVEC after being cultured with ADF and F‐ADF extracts for 24 h, with scratch assay (*n* = 8 per group). (P) Tube formation assessment of HUVEC, (Q) number of conjunction points, and (R) total vessel length after being cultured with ADF and F‐ADF extracts for 24 h (*n* = 8 per group). *p < 0.05; **p < 0.01; ***p < 0.001; “ns” means non‐significant difference.

EdU (5‐Ethynyl‐2'‐deoxyuridine) labeling revealed significantly higher proliferation rates in both ADF (23.57 ± 3.59%) and F‐ADF (22.15 ± 4.25%) groups compared to controls (6.75 ± 1.24%, *p* < 0.001), with no significant difference between ADF and F‐ADF groups (*p* = 0.8206) (Figure 3A,B). Consistent with these findings, CCK‐8 (Cell Counting Kit‐8) assays showed time dependent enhancement of ADSCs proliferation, with ADF and F‐ADF exhibiting similar growth curves that were markedly superior to controls at all time points (24 h to 72 h, *p* < 0.001) (Figure 3E). ADF and F‐ADF significantly enhanced ADSC migration in Transwell assays. Compared to controls (10.75 ± 4.99 cells per field), both materials induced a >3 fold increase in migrated cells (ADF: 37.25 ± 7.54; F‐ADF: 38.50 ± 8.34 cells per field, *p* < 0.001), with comparable efficacy between groups (*p* = 0.9667) (Figure 3C,D). These results collectively demonstrate that both ADF and F‐ADF effectively promote ADSCs proliferation and migration without compromising bioactivity.

Oil Red O quantification showed ADF (36.44 ± 3.53%) and F‐ADF (37.23 ± 4.28%) significantly enhanced ADSC lipid accumulation versus controls (19.79 ± 2.97%, *p* < 0.001). No statistically significant difference was observed between ADF and F‐ADF groups (*p* = 0.9491), indicating equivalent adipogenic potential (Figure 3F,G). At the molecular level, western blot analysis confirmed substantial upregulation of key adipogenic transcription factors in both ADF and F‐ADF groups. PPARγ protein expression increased 2.17 ± 0.13 fold in ADF and 2.23 ± 0.18 fold in F‐ADF compared to controls (*p* < 0.001), while C/EBPα expression rose 1.72 ± 0.17 fold in ADF and 1.69 ± 0.16 fold in F‐ADF (*p* < 0.001). No significant differences were detected between ADF and F‐ADF for either marker (*p* = 0.7240 in PPARγ and *p* = 0.6831 in C/EBPα) (Figure 3H,I). These protein‐level findings were corroborated by qPCR (quantitative polymerase chain reaction) analysis at the transcriptional level. PPARγ mRNA expression increased 2.36 ± 0.62 fold in ADF and 2.43 ± 0.55 fold in F‐ADF (*p* < 0.001), while C/EBPα mRNA levels rose 5.58 ± 0.99 fold in ADF and 5.37 ± 0.61 fold in F‐ADF (*p* < 0.001). Again, intergroup comparisons showed no statistically significant variation (*p* = 0.9698 in PPARγ and *p* = 0.8825 in C/EBPα; *n* = 8 per group) (Figure 3J). The combined phenotypic and molecular evidence demonstrates that ADF and F‐ADF effectively promote adipogenesis through activation of the PPARγ and C/EBPα signaling pathway. Enhanced adipogenesis and marker expression confirm ADF/F‐ADF's therapeutic potential for adipose regeneration.

Both ADF and F‐ADF boosted vascularization through complementary mechanisms. ADF/F‐ADF co‐cultures significantly increased HUVEC proliferation (ADF: 49.86 ± 3.87%; F‐ADF: 53.46 ± 5.86%) compared to controls (34.75 ± 2.99%, *p* < 0.001) (Figure 3K,L). Findings corroborated by CCK‐8 assays showed similar optical density increases (24–72 h, *p* < 0.001) at 72 h (2.37 ± 0.12 for ADF, 2.57 ± 0.17 for F‐ADF vs. 1.37 ± 0.08 for controls; *p* < 0.001) (Figure 3M). Scratch assays showed 78.12 ± 5.34% wound closure with ADF and 76.81 ± 4.79% with F‐ADF vs. 58.24 ± 4.09% in controls at 48 h (*p* < 0.001) (Figure 3N,O). Most critically, Matrigel tube formation assays quantified superior neovascularization capacity (Figure 3P), with ADF and F‐ADF producing significantly greater total vessel length (835.87 ± 35.52 mm per field and 840.99 ± 38.61 mm per field respectively vs. 518.75 ± 23.08 mm per field in controls at 10 h, *p* < 0.001) (Figure 3Q) and conjunction point formation (53.31 ± 2.27 and 55.75 ± 2.28 conjunctions per field vs. 33.25 ± 1.47 in controls at 10 h, *p* < 0.001) (Figure 3R). Throughout all assays (*n* = 8 replicates per group), no statistically significant differences emerged between ADF and F‐ADF performance (all intergroup *p *> 0.05). The concordant results obtained from proliferation, migration, and tube formation assays collectively demonstrate that both ADF and F‐ADF effectively promote angiogenesis through multiple synergistic mechanisms, thereby establishing their potential as ideal scaffold materials for vascularized tissue engineering applications and as angiogenesis‐promoting adjuncts.

The results also demonstrated that both ADF and F‐ADF significantly enhanced the proliferation and migration of key skin cell populations. For HDF, EdU assays revealed proliferation rates of 18.87 ± 3.56% (ADF) and 17.97 ± 2.45 % (F‐ADF) versus 7.25 ± 2.23% (control, *p* < 0.001) (Figure S3A,B, Supporting Information), with CCK‐8 assays showing similar optical density increases (24 to 72 h, *p* < 0.001) (Figure S3I, Supporting Information). HDF migration was similarly enhanced, with transwell assays demonstrating 70.54 ± 3.11 (ADF) and 65.25 ± 3.09 (F‐ADF) migrated cells versus 33.51 ± 4.25 (control, *p* < 0.001) (Figure S3C,D, Supporting Information). Functional assays demonstrated comprehensive enhancement of HaCaT cell activity by ADF and F‐ADF, with EdU proliferation assays revealing significantly increased replication rates (ADF: 63.38 ± 2.99% and F‐ADF: 67.39 ± 8.32%) compared to controls (40.03 ± 3.76%, *p* < 0.001). CCK‐8 viability testing confirmed sustained metabolic activity from 24 to 72 h (*p* < 0.001 vs. control), while Transwell migration assays showed approximately twofold greater cell movement (ADF: 64.25 ± 4.57 and F‐ADF: 64.00 ± 2.94 cells) relative to untreated groups (39.75 ± 4.03 cells, *p* < 0.001), collectively indicating potent stimulation of keratinocyte function (Figure S3E–H,J, Supporting Information). Notably, no significant differences were observed between ADF and F‐ADF groups in any assay (all *p* > 0.68), indicating comparable efficacy in promoting both fibroblast and keratinocyte functions critical for skin regeneration. These findings strongly suggest that both ADF and F‐ADF equally enhance the proliferative and migratory capacities of essential skin cell types, supporting their potential application in wound healing and tissue repair.

### 2.4 Protein Composition and Function Analysis of ADF

**FIGURE 4:**
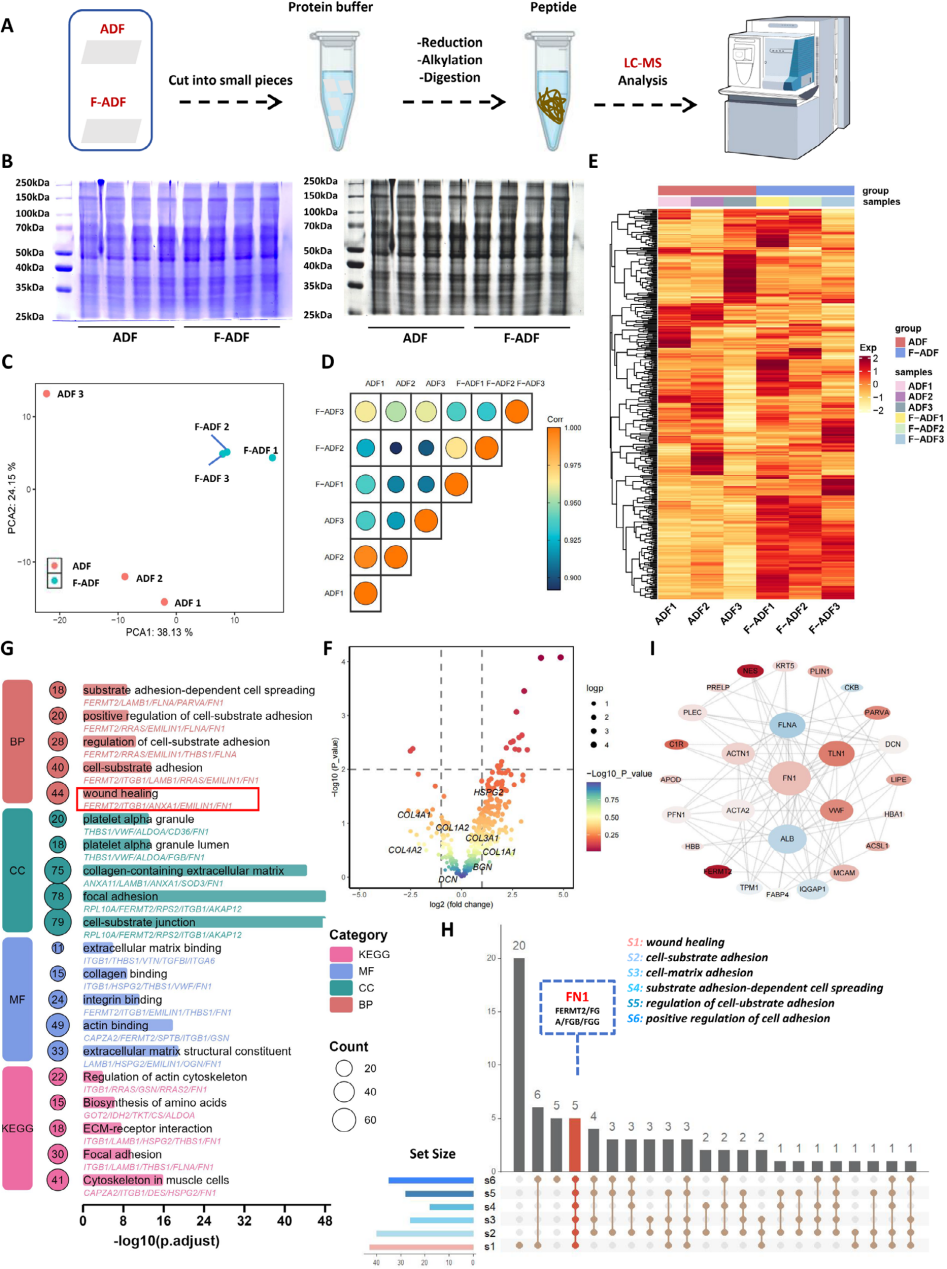
Protein composition and function analysis of ADF. (A) Schematic workflow of proteomic analysis. (B) Total protein profiles visualized by Coomassie Brilliant Blue staining. (C) Principal component analysis (PCA) scatter plot comparing ADF and F‐ADF. (D) Correlation matrix of protein expression profiles across ADF and F‐ADF samples. (E) Hierarchical clustering heatmap of differentially expressed proteins. (G) Functional enrichment analysis was performed on the 100 most abundant proteins identified in ADF. (H) Upset plot analysis of proteins among the different gene sets S1–S6. (I) Protein–protein interaction (PPI) network of high‐confidence interactors.

The ECM constitutes a complex network of various proteins, serving as a reservoir for growth factors and bioactive molecules. Therefore, we utilized proteomics to detect and analyze the active components in ADF and F‐ADF. Total proteins were extracted from ADF and F‐ADF samples using RIPA lysis buffer, enriched with protease inhibitors and phenylmethylsulfonyl fluoride (Figure 4A). The samples underwent reduction and subsequent digestion. The resulting peptides were purified and subjected to analysis (Figure 4A). To validate protein integrity, we performed SDS‐PAGE analyses of ADF and F‐ADF samples with Coomassie Blue staining (Figure 4B). The results analysis revealed well‐resolved protein band patterns spanning almost the full molecular weight range (25–250 kDa). The observed sharp band boundaries indicated preserved protein integrity, with consistent detection of characteristic ECM components, particularly collagen I (140 kDa). These findings collectively validate the effectiveness of our extraction protocol in maintaining native ECM composition.

Our integrated proteomic analysis demonstrates remarkable compositional similarity between ADF and F‐ADF through a comprehensive multi‐modal approach. Principal component analysis (PCA) of global proteomic profiles revealed substantial overlap between sample groups, with the first two principal components PC1 (24.15%) and PC2 (38.13%), collectively explaining 62.28% of total variance, indicating these dimensions captured the majority of biologically relevant variation (Figure 4C). The observed dispersion within groups reflects our intentional inclusion of samples from three distinct individuals per group (female, age 30–40, BMI 19–24) to validate methodological robustness, whereas the overlapping distributions between ADF and F‐ADF clusters suggest minimal cryopreservation‐induced alterations (Figure 4C). This interpretation is further supported by correlation analyses showing strong intra‐group (0.925–0.950) and inter‐group (0.900–0.975) correlations (Figure 4D), as well as hierarchical clustering patterns that visually confirm the absence of significant segregation between treatment groups (Figure 4E). Differential expression analysis using stringent criteria (log_2_FC > 1, log_10_ *p*‐value > 2) identified only 24 significantly altered proteins among 418 detected species, representing just 5.7% of the total proteome (Figure 4F). It is particularly noteworthy that our mass spectrometry analysis revealed no statistically significant differences in the content of various ECM proteins between the groups, including freeze‐sensitive components such as heparan sulfate proteoglycans, Col4A1, Col4A2, Col3A1, BGN, and DCN, as well as structurally stable proteins like Col1A1 and Col1A2 (Figure 4F).

The uniform expression distribution across abundance ranges, as confirmed by MA plots, and the consistent findings across complementary analytical platforms (PCA, correlation matrices, heatmaps, and volcano plots) collectively provide robust evidence for the high degree of proteomic conservation between ADF and F‐ADF preparations. This strategy combines unsupervised pattern recognition with supervised statistical testing, ensuring comprehensive evaluation of potential processing effects while controlling for biological variability inherent in human‐derived samples. The convergence of results across independent analytical modalities strongly supports the conclusion that the fibrin modification process preserves the essential protein composition and functional characteristics of the native adipose‐derived matrix.

Based on the compelling evidence of proteomic conservation, we proceeded with functional characterization of the top 100 most abundant proteins in ADF. Gene ontology (GO) analysis revealed that ADF‐associated proteins were significantly enriched in critical biological processes essential for tissue regeneration, particularly wound healing (GO: 0042060) and cell‐substrate adhesion (GO: 0031589) (Figure 4G). These proteins predominantly localize to collagen‐enriched extracellular matrices (GO: 0062023) and cell‐substrate junctions (GO: 0030057), demonstrating their structural role in maintaining tissue integrity (Figure 4G). Molecular functional analysis identifies three key capabilities: extracellular matrix binding (GO: 0050840), integrin‐mediated adhesion (GO: 0033627), and actin cytoskeletal interaction (GO: 0030864), which collectively facilitate the mechanotransductive processes underlying cellular migration and dynamic matrix remodeling (Figure 4G). KEGG (Kyoto Encyclopedia of Genes and Genomes) pathway analysis demonstrates statistically significant enrichment in three functionally interrelated pathways: focal adhesion (ko04510), ECM‐receptor interaction (ko04151), and amino acid biosynthesis (ko01230), establishing a mechanistic link between ADF's molecular composition and its regenerative potential through integrated structural, signaling, and metabolic functions (Figure 4G).

Through comprehensive protein–protein interaction (PPI) network analysis of the global proteome, we identified several pivotal functional mediators. Notably, FN1 (Fibronectin 1) emerged as a central extracellular matrix (ECM) organizer, while FERMT2 (Kindlin‐2) was implicated in integrin activation, and fibrinogen subunits (FGA/FGB/FGG) were associated with provisional matrix formation (Figure 4H). Subsequent application of the MCODE algorithm (implemented in Cytoscape with parameters) for PPI network construction and visualization revealed FN1, ALB, and VWF as primary hub genes (degree centrality > 40), with ACTB and HBA1 functioning as secondary regulators (Figure 4I). In the network visualization, node size represents interaction degree, while a color gradient (red: log_2_FC > 2; blue: log_2_FC < −1) indicates differential expression patterns and interaction strength (Figure 4I).

The observed proteomic conservation between ADF and F‐ADF, along with their congruent pathway enrichment profiles, provides compelling evidence that fibrin modification preserves critical bioactive components while maintaining structural and functional integrity. These findings collectively establish the ADF‐derived matrices as functionally optimized scaffolds capable of orchestrating physical support through ECM architecture, mediating regenerative signaling via growth factor sequestration, and facilitating dynamic reciprocity through cell‐matrix feedback loops, thereby creating a pro‐regenerative microenvironment that promotes tissue repair.

### 2.5 ADF Promotes Tissue Regeneration and Remodeling, While Maintaining Its Shape and Performance

**FIGURE 5:**
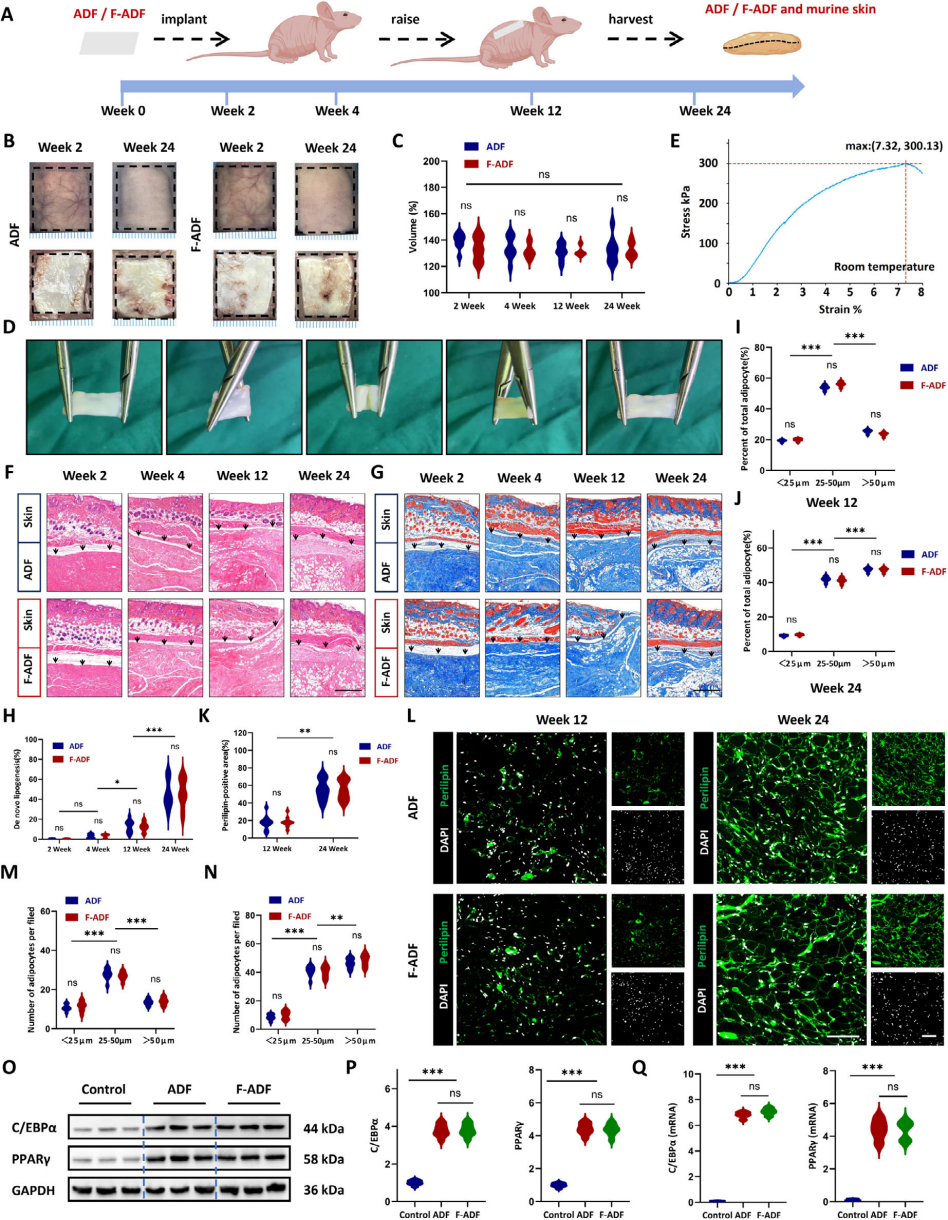
Tissue regeneration and remodeling of ADF. (A) Schematic of ADF/F‐ADF implantation. (B) Gross morphology of implants at 2 and 24 weeks post‐implantation. (C) Volume assessment of ADF and F‐ADF at 2, 4, 12, and 24 weeks post‐implantation. (D) Digital images of the ADF and F‐ADF post‐implantation under different operations (bend, twist, and stretch). (E) Tensile stress–strain of ADF post‐implantation at room temperature. (F) Hematoxylin and eosin staining of ADF and F‐ADF at intervals of 2, 4, 12, and 24 weeks post‐implantation with overlying murine skin. Scale bar = 500 µm. (G) Masson's trichrome staining of ADF and F‐ADF at intervals of 2, 4, 12, and 24 weeks post‐implantation with overlying murine skin. Scale bar = 500 µm. (H) Quantification of adipose tissue regeneration volume (%). (I,J) Adipocyte size distribution (< 25 µm, 25–50 µm, > 50 µm) at 12, 24 weeks post‐implantation. (K,L) Perilipin immunofluorescence (green) and positive area analysis. Scale bar = 100 µm. (M,N) Size distribution of perilipin‐positive adipocytes (< 25 µm, 25–50 µm, > 50 µm) at 12, 24 weeks post‐implantation. (O) Western blot analysis of C/EBPα and PPAR γ (*n* = 8 per group). (P) qPCR analysis of C/EBPα and PPAR γ (*n* = 8 per group). Dotted lines: implant‐host tissue interface. *p < 0.05; **p < 0.01; ***p < 0.001; “ns” means non‐significant difference.

ADF and F‐ADF exhibit bioactive effects in vitro, stimulating ADSCs proliferation (Figure 3A,B) and migration (Figure 3C,D), promoting adipogenesis (Figure 3F,G), and enhancing angiogenesis (Figure 3K–R). To evaluate their regenerative performance in vivo, we performed subcutaneous implantation in nude mice dorsal regions, with tissue sampling at 2, 4, 12, and 24 weeks post‐operation (Figure 5A). Both ADF and F‐ADF displayed exceptional structural stability following implantation, maintaining the original architecture with minimal distortion (Figure 5B and Figure S4A, Supporting Information). Quantitative volumetric measurements revealed significant expansion to 139.29 ± 5.95% (ADF) and 133.58 ± 9.14% (F‐ADF) of original volumes by 2 weeks (*p* < 0.001 vs. pre‐implantation, *n* = 8 per group). The expanded volume remained remarkably stable throughout the study period (4 weeks: 134.08 ± 8.67% ADF, 131.79 ± 5.56% F‐ADF; 12 weeks: 131.15 ± 6.09% ADF, 131.61 ± 3.75% F‐ADF; 24 weeks: 132.39 ± 9.95% ADF, 132.48 ± 5.89% F‐ADF, *p* > 0.05 for all inter‐timepoint comparisons by two‐way ANOVA) (Figure 5C). The observed volumetric stability closely matched in vitro swelling equilibrium measurements (Figure 1N), suggesting tissue fluid absorption at the early stage post‐implantation. These findings collectively validate that both ADF and F‐ADF maintain dimensional stability and structural integrity under physiological conditions, fulfilling essential criteria for reliable performance in regenerative medicine.

To further assess the mechanical properties after regeneration, as shown in Figure 5D, ADF demonstrated good mechanical performance, being stretchable, foldable, and capable of returning to its original form. Stress–strain analyses demonstrated characteristic J‐shaped curves at both room temperature (Figure 5E) and 37°C (Figure S4B, Supporting Information), with no statistically significant differences between conditions (*p* = 0.8428). Ultimate tensile strength decreased from 383.35 ± 16.41 kPa pre‐implantation to 298.73 ± 18.26 kPa in vivo (*p* = 0.0120), while strain capacity increased from 5.36 ± 0.52% to 7.15 ± 1.54% (*p* = 0.0086) (Figure 1J,5E). Functional testing confirmed maintained foldability and shape memory properties (Video S2, Supporting Information). These collective findings demonstrate the materials' stable integration and controlled regenerative performance in biological environments.

Histological evaluation of implanted ADF and F‐ADF scaffolds revealed a well‐orchestrated, time‐dependent adipose tissue regeneration process (Figure 5F,G). Initial assessment by HE staining demonstrated that both ADF and F‐ADF maintained homogeneous dense structures at the marginal zones during the first 2 weeks post‐implantation (Figure 5F). The regenerative process became evident by 4 weeks, with the emergence of small adipocyte‐like cells, suggesting early adipocyte differentiation. Importantly, quantitative analysis showed progressive adipose tissue formation, with adipose‐like structures appearing in both marginal and central zones by 12 weeks. Ultimately, the tissue developed into adipose tissue, occupying 45.70 ± 14.06% of the total area by 24 weeks (*p* < 0.001 vs. 12 weeks) (Figure 5H). Masson's trichrome staining provided crucial insights into the extracellular matrix remodeling process accompanying adipogenesis. At 2 weeks post‐implantation, collagen fibers maintained a dense and organized architecture (Figure 5G). However, by 4 weeks, we observed significant collagen reorganization coinciding with the formation of adipose tissue niches surrounded by collagen fibers (Figure 5G). This structural remodeling created a permissive microenvironment for subsequent adipose tissue expansion.

We used ImageJ to analyze HE sections at two critical time points in adipose regeneration (12 and 24 weeks) to quantify lipid droplet number and diameter (immature adipocytes: < 25 µm; transitional adipocytes: 25–50 µm; mature adipocytes: > 50 µm) (Figure 5I,J). Detailed morphometric analysis of adipocyte maturation revealed distinct temporal patterns. Transitional adipocytes (25–50 µm diameter) predominated at 12 weeks (68.3 ± 5.2% of total adipocytes), while mature adipocytes (> 50 µm) became the major population by 24 weeks (54.7 ± 4.8%) (Figure 5I,J). Notably, no significant differences were observed between ADF and F‐ADF groups in any maturation parameter (*p* > 0.05), indicating comparable adipogenic potential.

Perilipin immunofluorescence demonstrated progressive adipogenesis, increasing from 17.91 ± 7.87% positive area at 12 weeks to 55.51 ± 11.87% at 24 weeks (*p* < 0.001) (Figure 5K,L). Quantitative analysis of perilipin^+^ adipocytes (categorized by size and consistent with HE staining) revealed dynamic maturation in ADF groups. Immature (10.75 ± 1.67%), transitional (27.75 ± 2.92%), and mature adipocytes (13.50 ± 1.62%) at week 12, progressed to 8.63 ± 2.07%, 40.76 ± 3.84%, and 46.54 ± 3.46%, respectively, by week 24, demonstrating clear temporal maturation (Figure 5M,N). Most significantly, western blot and qPCR analyses demonstrated significant upregulation of adipogenic master regulators in regenerated ADF (12‐week post‐implantation) compared to control skin, with PPARγ showing 4.42 ± 0.35 fold (protein) and 4.41 ± 0.64 fold (mRNA) increases, and C/EBPα exhibiting 3.78 ± 0.34 fold (protein) and 6.81 ± 0.24 fold (mRNA) enhancements (*p* < 0.001) (Figure 5O–Q). No statistically significant differences were observed between ADF and F‐ADF (PPARγ mRNA 4.34 ± 0.58 fold, protein 4.37 ± 0.36 fold, and C/EBPα mRNA 7.07 ± 0.28 fold, protein 3.80 ± 0.33 fold, *p* > 0.05 for all comparative analyses) (Figure 5O–Q).

These molecular findings provide compelling evidence for the adipogenic commitment of the regenerated tissue. The cytokine microenvironment analysis revealed a precisely timed inflammatory phase followed by resolution. Pro‐inflammatory IL‐6 levels peaked at 2 weeks in both ADF (19.94 ± 1.53 pg/mg) and F‐ADF (20.45 ± 1.62 pg/mg) groups, showing significant elevation compared to controls (5.04 ± 0.98 pg/mg, *p* < 0.001) (Figure S4C,D, Supporting Information). Conversely, anti‐inflammatory IL‐10 demonstrated progressive upregulation, reaching maximal levels by 4 weeks (ADF: 15.64 ± 1.53 pg/mg and F‐ADF: 15.32 ± 1.47 pg/mg) that were significantly higher than both the 2‐week timepoint (*p* < 0.001) and control groups (*p* < 0.001) (Figure S4C,D, Supporting Information). This coordinated cytokine shift (IL‐6 decline and IL‐10 rise) temporally aligned with histological evidence of adipogenesis initiation (Figure S4C,D, Supporting Information).

Our quantitative analysis reveals that ADF scaffolds orchestrate spatiotemporal adipose regeneration through a coordinated biological cascade. It promoted ECM remodeling to establish adipogenic niches while precisely regulating inflammatory resolution (IL‐6/IL‐10 shift). The scaffolds robustly activate the PPARγ and C/EBPα pathway in ADF and F‐ADF, confirming preserved regenerative capacity post‐cryopreservation. These findings strongly support the clinical potential of ADF‐based regeneration strategies

### 2.6 Cell Infiltration‐Mediated Angiogenesis Plays a Crucial Role in ADF Regeneration and Remodeling

**FIGURE 6:**
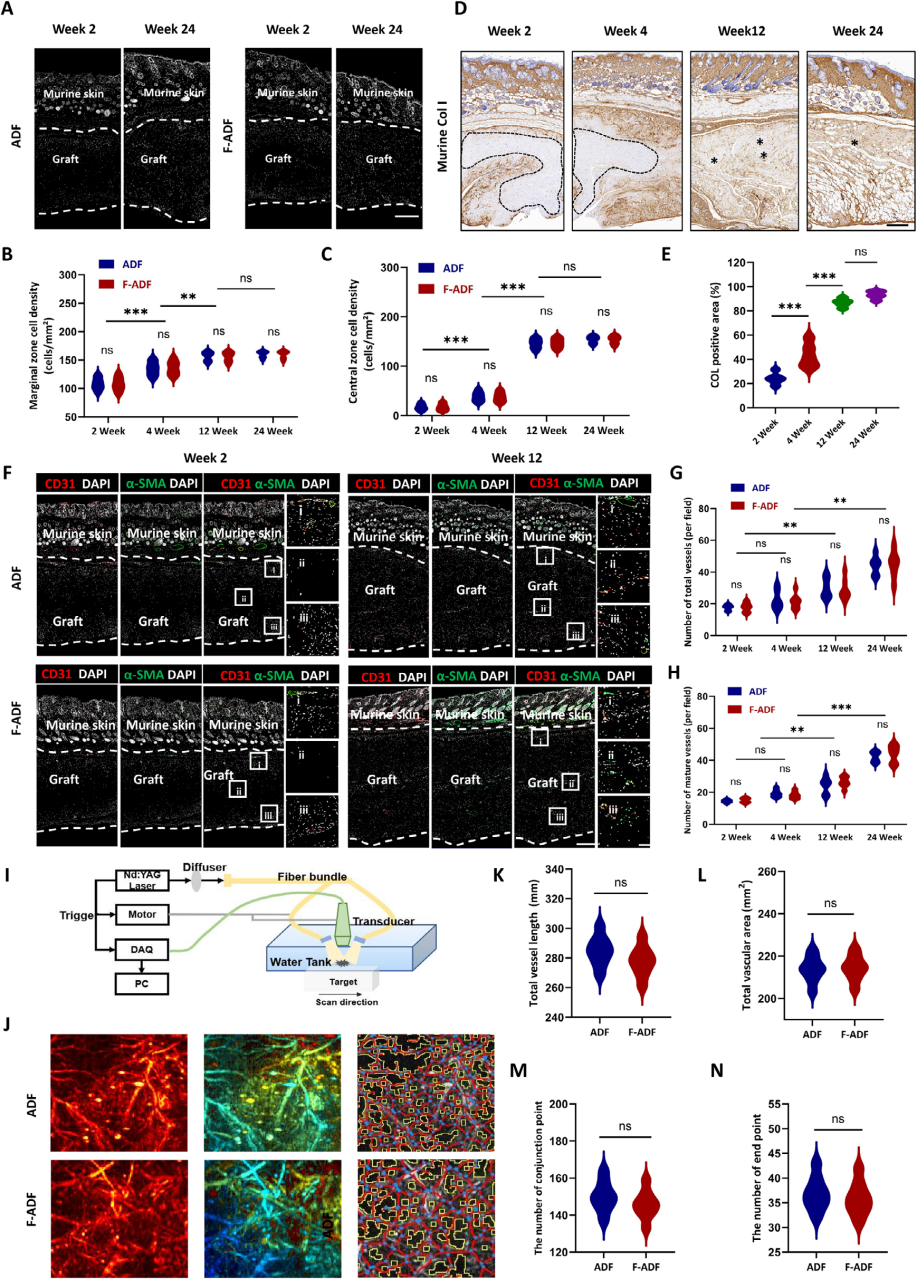
Cell infiltration‐mediated angiogenesis plays a crucial role in ADF regeneration and remodeling. (A) Cellular infiltration into ADF/F‐ADF implants with (B) marginal and (C) central zone quantification (*n* = 8 per group). Scale bar = 300 µm. (D,E) Murine collagen I immunohistochemistry (brown) of ADF and F‐ADF post‐implantation and analysis. Scale bar = 500 µm. Dotted lines and * indicate the unreplaced area of implanted ADF and F‐ADF. (F) Immunofluorescent staining (CD31 red and α‐SMA green) of ADF and F‐ADF post‐implantation with overlying murine skin and (G) total vessels and (H) mature vessels analysis. Scale bars, 300 µm in overview images (left) 200 µm in magnified images (right). Dotted lines: implant‐host tissue interface. (I) Photoacoustic Imaging (PAI) for vessel detection schematic diagram. (J) 3D reconstruction image of vessels (left: origin 3D image; middle: 3D image with depth information; right: vessels fitting analysis plot) and analysis for (K) total vessel length, (L) total vascular area, (M) number of conjunction points, and (N) number of end points. *p < 0.05; **p < 0.01; ***p < 0.001; “ns” means non‐significant difference.

Cellular infiltration serves as the primary driver for tissue regeneration and remodeling [31, 32]. To evaluate this process, we performed immunofluorescence staining at 2, 4, 12, and 24 weeks post‐implantation (Figure 6A and Figure S4E, Supporting Information). DAPI nuclear staining (white light) and quantitative analysis of infiltrating cells within the implanted ADF and F‐ADF (dashed‐line demarcated zones) revealed distinct spatial‐temporal patterns. The grafts were divided into marginal (upper and lower quartiles) and central (middle half) zones for analysis (Figure 6A and Figure S4E, Supporting Information). At 2 weeks, cellular infiltration was predominantly localized to the peripheral regions (108.25 ± 11.17 cells/mm^2^) and little localized to the central zones (17.38 ± 6.72 cells/mm^2^) (Figure 6B,C). Over time, infiltration progressively extended toward the central regions, achieving uniform distribution by 12 weeks (central zones: 145.75 ± 10.29 cells/mm^2^). By 24 weeks, cellular density plateaued (153.63 ± 7.39 cells/mm^2^) (Figure 6B,C). Quantitative analysis revealed comparable cellular infiltration patterns between ADF and F‐ADF groups, with no statistically significant differences observed (*p* > 0.05 for all comparative metrics). We hypothesize that upon reaching a critical infiltration threshold, the process shifts toward orchestrating lineage‐specific differentiation, thereby driving tissue regeneration and remodeling. These findings align closely with histological assessments of adipogenesis, underscoring infiltrating cells as pivotal regulators of tissue regeneration.

Tissue regeneration involves gradual scaffold degradation and replacement by host‐derived components [33, 34]. To monitor this remodeling process in human‐derived ADF, containing abundant collagen, we implemented species‐specific immunohistochemistry targeting mouse collagen (Figure 6D). This method precisely tracked graft replacement dynamics, differentiating host versus donor matrix contributions. Host collagen replacement progressed from marginal zones (24.21 ± 4.77% at 2 weeks) to central regions (43.63 ± 9.66% at 4 weeks), reaching near‐complete remodeling by 24 weeks (93.10 ± 3.21%) (Figure 6D,E). This spatiotemporal pattern correlated strongly with cellular infiltration, confirming host cell‐mediated functional scaffold remodeling.

Given the interdependence of cellular infiltration and angiogenesis, we evaluated vascularization using CD31 (total vessels) and α‐SMA/CD31 co‐staining (mature vessels) (Figure 6F). Skin fluorescence from normal nude mice served as controls (Figure S4G, Supporting Information). Neovascularization mirrored infiltration dynamics, progressing from marginal to central (Figure 6G,H, and Figure S4E, Supporting Information). Both total and mature vessel densities increased significantly from 2 to 24 weeks in ADF and F‐ADF (Figure 6G,H and Figure S4E, Supporting Information). To further characterize neovascularization, photoacoustic imaging with 3D vascular reconstruction was performed at 12 weeks (Figure 6I,J). Reconstructed images (left: 3D vasculature, middle: depth map, right: fitted vessels) demonstrated robust, morphologically mature vasculature in both groups (Figure 6I,J). Quantitative analysis of vascular length (ADF: 284.82 ± 10.38 and F‐ADF: 277.95 ± 12.43, *p* = 0.2023) (Figure 6K), vascular area (ADF: 213.7 ± 6.19 and F‐ADF: 214.4 ± 8.76, *p* = 0.8343) (Figure 6L), conjunction points (ADF: 150.21 ± 8.49 and F‐ADF: 145.67 ± 7.84, *p* = 0.3021) (Figure 6M), and end points (ADF: 37.25 ± 4.83 and F‐ADF: 36.49 ± 4.58, *p* = 0.9160) (Figure 6N) revealed no significant intergroup differences. These results collectively indicate that infiltrating cells orchestrate sustained angiogenesis and vascular maturation, ultimately facilitating ADF regeneration and remodeling.

### 2.7 Macrophages Are Key Infiltrating Cells Mediating Angiogenesis, Regeneration, and Remodeling of ADF

**FIGURE 7:**
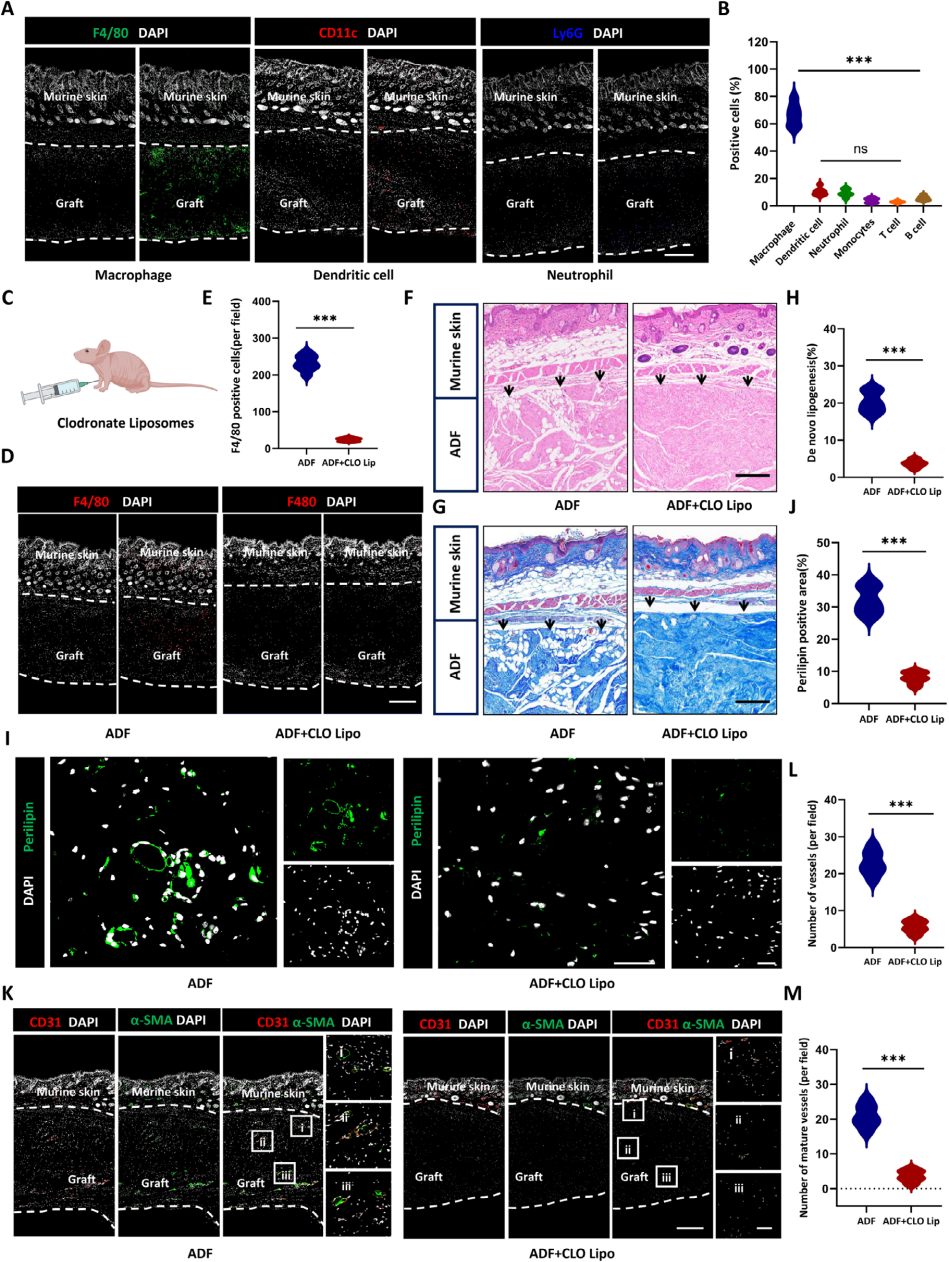
Macrophages as key modulators of ADF regeneration and angiogenic remodeling. (A) Immunofluorescent staining of ADF and F‐ADF post‐implantation with overlying murine skin and (B) analysis (F4/80 for macrophages, CD11c for dendritic cells, and Ly6G for neutrophils). Scale bar = 300 µm. Dotted lines: implant‐host tissue interface. (C) clodronate liposome traperitoneal administration for macrophage depletion. (D) Immunofluorescence staining of F4/80^+^ macrophages in ADF implants: clodronate liposome‐treated (right) versus control (left), with quantification (E). Scale bar = 300 µm. (F) HE staining of ADF post‐implantation: clodronate liposome‐treated (right) versus control (left). Scale bar = 300 µm. (G,H) Masson's trichrome staining of ADF post‐implantation: clodronate liposome‐treated (right) versus control (left) and analysis of adipose tissue regeneration volume (%). Scale bar = 300 µm. (I,J) Immunofluorescent staining (perilipin green) of ADF post‐implantation with (right) and without clodronate liposome (left) and analysis. Scale bar = 100 µm. (K) Immunofluorescent staining (CD31 red and α‐SMA green) of ADF post‐implantation: clodronate liposome‐treated (right) versus control (left) and (L) total vessels and (M) mature vessels analysis. Scale bars, 300 µm in overview images (left) 200 µm in magnified images (right). *p < 0.05; **p < 0.01; ***p < 0.001; “ns” means non‐significant difference.

Previous findings highlighted the critical involvement of infiltrating cells in vascularization during ADF regeneration. To delineate specific cellular contributions, we performed comprehensive immunofluorescence staining for key immune cell populations at time points of 4 weeks post‐implantation, including macrophages (F4/80^+^), dendritic cells (CD11c^+^), neutrophils (Ly6G^+^), monocytes (CD14^+^), T lymphocytes (CD3^+^), and B lymphocytes (CD19^+^) (Figure 7A and Figure S4I, Supporting Information). Skin fluorescence of F4/80 from normal nude mice served as controls (Figure S4H, Supporting Information). Quantitative analysis revealed macrophages as the predominant population (66.64 ± 8.46%), significantly outnumbering the aggregate of other cell types (dendritic cells: 10.39 ± 2.97%, neutrophils: 9.32 ± 2.76%, monocytes: 4.09 ± 1.75%, T lymphocytes: 2.82 ± 0.84%, and B lymphocytes: 5.59 ± 1.78%, *p* < 0.001) (Figure 7B). This striking cellular hierarchy strongly suggested macrophages might serve as the master regulators in ADF remodeling. To delineate the causative role of macrophage infiltration in tissue regeneration, we performed targeted macrophage depletion via clodronate liposomes (intraperitoneal injection every 3 days at a dose of 150 µL per 20 g body weight) in ADF‐implanted nude mice (Figure 7C). Compared to controls, clodronate liposome (CLO lipo) treatment effectively depleted macrophages, reducing F4/80^+^ cell counts from 232.75 ± 21.23 to 24.50 ± 4.93 cells per field (*p* < 0.001) (Figure 7D,E).

Subsequent histological analysis revealed severely impaired tissue regeneration in macrophage‐depleted mice. Both HE and Masson's trichrome staining showed significantly reduced adipose tissue formation (ADF: 20.33 ± 2.83% and ADF + CLO lipo: 3.71 ± 1.03%, *p* < 0.001) (Figure 7F–H). Similarly, perilipin^+^ adipocyte area decreased markedly from 32.19 ± 4.25% to 8.03 ± 1.79% (*p* < 0.001) (Figure 7I,J). Notably, CD31^+^ total vessels and α‐SMA^+^/CD31^+^ mature vessels were reduced by 75.39% and 82.61%, respectively (*p* < 0.001) (Figure 7K–M). These quantitative results provide conclusive evidence that macrophages are essential regulators of both adipogenesis and angiogenesis in ADF regeneration.

Traditional research divides macrophages into M1 and M2 types, and M2 macrophages have been widely reported to promote regenerative repair, including tissue regeneration and wound healing [35]. Therefore, we next characterized phenotype dynamics throughout the regeneration process. During the active regeneration phase (12 to 24 weeks), co‐staining for F4/80 and CD206 (M2 marker) revealed abundant M2 macrophages in both ADF (72.4 ± 6.1%) and F‐ADF (70.8 ± 5.7%, *p* = 0.4281). Skin fluorescence from normal nude mice served as controls, demonstrating that sham‐operated groups exhibited neither macrophage infiltration nor polarization changes in subcutaneous tissue, thereby excluding potential confounding effects from surgical procedures during model establishment (Figure S5A,C, Supporting Information). Further phenotyping using iNOS (M1 marker) and CD206 confirmed an M2‐dominant microenvironment (iNOS^+^/CD206^+^ ratio: ADF 0.16 ± 0.02 and F‐ADF: 0.16 ± 0.01, *p* = 0.8514) (Figure S5D,E, Supporting Information).

To elucidate the temporal dynamics of macrophage polarization initiation, we focused on the critical phenotypic transition window (weeks 2 to 4 post‐implantation). Quantitative analysis of polarization markers revealed distinct kinetic patterns. iNOS expression peaked at week 2 (12.38 ± 1.19 fold increase vs. baseline, *p* < 0.01), followed by significant resolution by week 4 (8.16 ± 0.59 fold, *p* < 0.01) (Figure S5F, Supporting Information). Conversely, Arg1 (M2 marker) exhibited progressive upregulation during this period (2 weeks: 3.49 ± 0.41 fold and 4 weeks: 22.32 ± 1.24 fold, *p* < 0.001) (Figure S5G, Supporting Information). Consequently, the iNOS/Arg1 ratio decreased sharply from 3.56 ± 0.12 to 0.37 ± 0.05 (*p* < 0.001) (Figure S5H, Supporting Information), mirroring the IL‐6/IL‐10 cytokine dynamics we previously observed (Figure S4C,D, Supporting Information).

This sequential polarization (M1 to M2 transition) precisely correlates with the cytokine shift required for regenerative progression, demonstrating that macrophages orchestrate ADF remodeling through phenotype dependent regulation of the tissue microenvironment. Our findings delineate a self‐amplifying regenerative cascade wherein early M1 macrophage infiltration initiates pro‐regenerative inflammation, followed by timely M2 polarization that facilitates extracellular matrix reorganization, while synchronized cytokine profile transitions collectively drive adipogenic differentiation and vascular network formation. Importantly, the conserved macrophage dependence between ADF and F‐ADF suggests these findings represent a fundamental mechanism of ECM scaffold‐mediated regeneration. The aforementioned results further illustrate the key role of infiltrating cells‐mediated angiogenesis in ADF regeneration and remodeling.

### 2.8 Recruitment of Circulating Cells Is Promoted in ADF Regeneration and Remodeling

**FIGURE 8:**
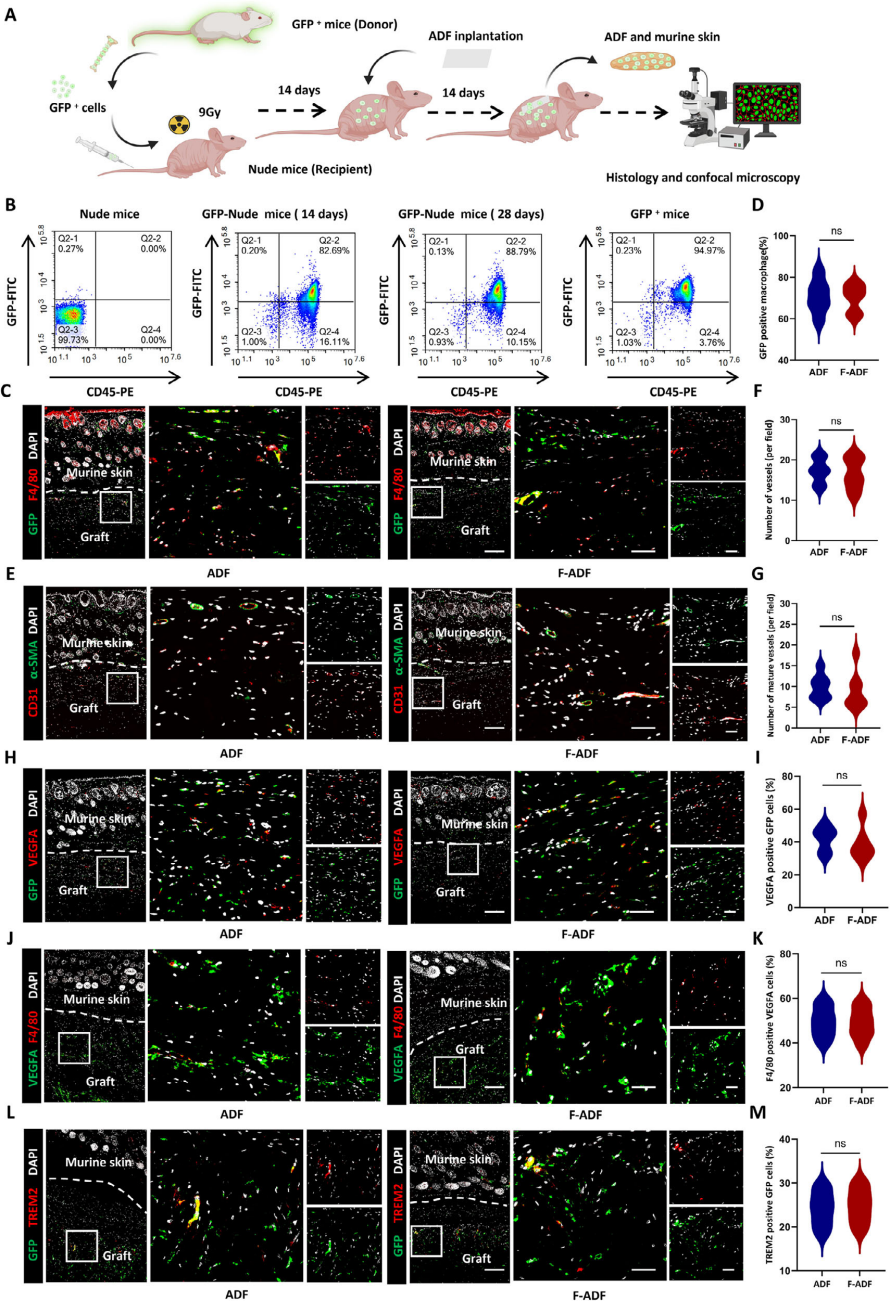
Recruitment of circulating cells is promoted in ADF regeneration and remodeling. (A) Schematic of GFP^+^ bone marrow transplantation from donor mice to nude mouse recipients. (B) Flow cytometry quantification of GFP^+^CD45^+^ cells in recipient's peripheral blood. (C) Dual immunofluorescence of F4/80^+^ macrophages (red) and GFP^+^ cells (green) in ADF/F‐ADF implants. (D) Quantification of GFP^+^ F4/80^+^ macrophages (*n* = 8 per group). Scale bars, 500 µm in (left) and 100 µm in (right and middle). Dotted lines: implant‐host tissue interface. (E) Immunofluorescent staining (CD31 red and α‐SMA green) of ADF and F‐ADF post‐implantation with overlying murine skin. Quantification of (F) total number of vessels and (F) mature vessels of ADF and F‐ADF post‐implantation to nude mouse recipients (*n* = 8 per group). Scale bars, 500 µm in (left) and 100 µm in (right and middle). Dotted lines: implant‐host tissue interface. (H,I) Immunohistochemical staining (VEGFA red and GFP green) of ADF and F‐ADF post‐implantation to the nude mouse recipients with overlying murine skin and analysis (*n* = 8 per group). Scale bars, 500 µm in (left) and 100 µm in (right and middle). Dotted lines: implant‐host tissue interface. (J,K) Immunohistochemical staining (VEGFA green and F4/80 red) of ADF and F‐ADF post‐implantation to the nude mouse recipients with overlying murine skin and analysis (*n* = 8 per group). Scale bars, 500 µm in (left) and 100 µm in (right and middle). Dotted lines: implant‐host tissue interface. (L,M) Immunohistochemical staining (GFP green and TREM2 red) of ADF and F‐ADF post‐implantation to the nude mouse recipients with overlying murine skin and analysis (*n* = 8 per group). Scale bars, 500 µm in (left) and 100 µm in (right and middle). Dotted lines: implant‐host tissue interface. *p < 0.05; **p < 0.01; ***p < 0.001; “ns” means non‐significant difference.

Building upon our previous findings that identified macrophages (F4/80^+^) as the predominant infiltrating cell population (66.64 ± 8.46%) driving ADF regeneration (Figure 7B). To definitively trace the cellular origin of these macrophages, we established a bone marrow chimera model through transplantation of GFP^+^ bone marrow cells into nude recipients, enabling precise discrimination between circulating (GFP^+^) and tissue‐resident (GFP^−^) macrophage populations (Figure 8A).

To evaluate the proportion of GFP‐positive immune cells in peripheral blood after bone marrow transplantation, we performed flow cytometry (GFP‐FITC, CD45‐PE) to detect GFP^+^ CD45^+^ cells at day 14 (transplantation day) and day 28 (14 days post‐transplantation). Unlabeled normal nude mice served as blank controls (double negative), while CD45‐labeled GFP mice served as double positive controls. The analysis revealed an absence of positive cells in unlabeled nude mice, while GFP mice showed a high prevalence of double positive cells (95.46 ± 4.24%) (Figure 8B). Following transplantation, the percentage of double positive cells was 81.42 ± 6.17% at day 14 and rose to 89.75 ± 9.54% by day 28 (Figure 8B). These results confirmed the successful establishment of the GFP^+^ bone marrow chimera model.

Subsequently, we conducted dual immunofluorescence staining for GFP and F4/80 to delineate the cellular origin of infiltrating macrophages (Figure 8C). The analysis revealed extensive colocalization of GFP^+^ and F4/80^+^ signals within both ADF and F‐ADF, with GFP^+^ macrophages constituting approximately 70% (ADF: 71.38 ± 8.41% and F‐ADF: 70.55 ± 6.68%, *p* = 0.8946) of the total macrophage population (Figure 8D). These findings provide definitive evidence that circulating precursor cells differentiate into functional macrophages within the ADF microenvironment.

To characterize the vascularization patterns of the GFP^+^ bone marrow chimera model, we performed immunofluorescence staining using CD31 to identify total vasculature and α‐SMA/CD31 dual labeling to distinguish mature vessels (Figure 8E). Quantitative analysis demonstrated robust and comparable neovascularization surrounding both ADF and F‐ADF implants (Figure 8F,G). No significant differences were observed between groups, both total vessel density (ADF: 17.29 ± 3.09% vs. F‐ADF: 16.58 ± 3.91%, *p* = 0.7113) and mature vessel density (ADF: 10.12 ± 2.94% vs. F‐ADF: 9.35 ± 4.54%, *p* = 0.6347) (Figure 8F,G).

These data support a mechanistic paradigm that ADF and F‐ADF recruit circulating cells that undergo macrophage differentiation to modulate vascularization, and the newly formed vascular network facilitates sustained recruitment of circulating cells. This reciprocal interaction establishes a self‐amplifying cycle that likely drives the progressive regeneration and remodeling of ADF.

### 2.9 ADF Promotes Healing of Full‐Thickness Skin Defects Through Enhanced Vascularization and Tissue Regeneration

The impaired healing of extensive acute wounds represents a significant clinical challenge, underscoring the critical need for developing functional bioactive dressings to accelerate wound repair [36, 37]. Our experimental studies have demonstrated that ADF promotes tissue regeneration through macrophage recruitment and polarization mediated pro‐angiogenic effects, with a pivotal role of vascularization in the wound healing process. Furthermore, in vitro experiments have shown that ADF enhances the proliferation and migration capacities of both epidermal cells and fibroblasts, strongly suggesting its potential as a functional dressing for promoting the healing of large wounds.

**FIGURE 9:**
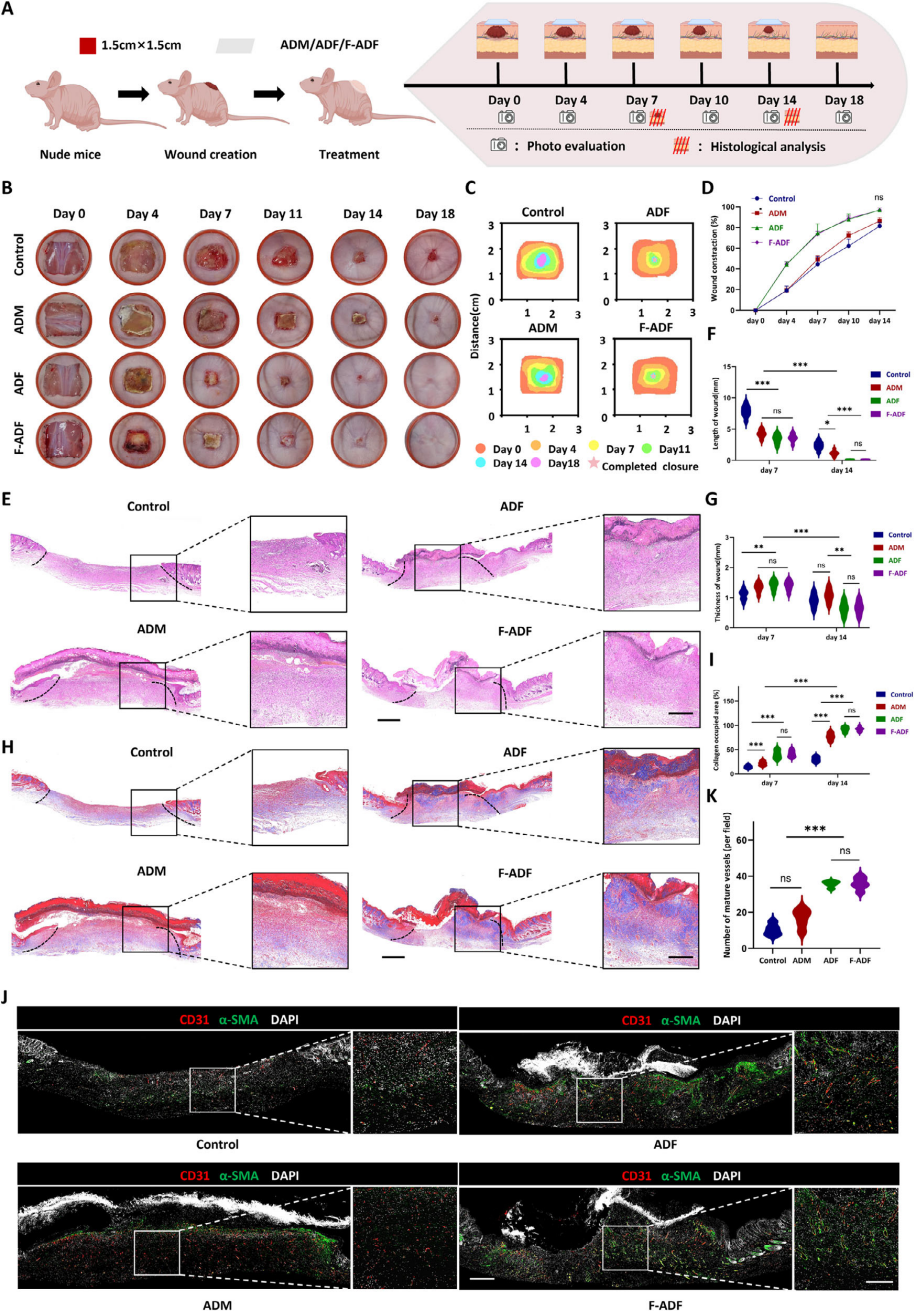
Treatment efficacy of ADF in a nude mouse wound model. (A) Experimental timeline of wound healing study. (B) Representative wound images from all treatment groups (control, ADM, ADF, F‐ADF) at days 0, 4, 7, 11, 14, and 18. (C) Schematic of wound margin tracing methodology for all groups. (D) Quantitative analysis of wound closure rate (%) over time (*n* = 8 per group). (E) HE and (H) Masson staining of wound beds at day 7. Scale bars, 500 µm in overview images (left) 200 µm in magnified images (right). (F) Wound epithelial gap and (G) granulation tissue thickness at days 7 and 14 (*n* = 8 per group). (I) Quantification of collagen deposition area (*n* = 8 per group). (J) Immunofluorescent staining (CD31 red and α‐SMA green) of all groups on day 7. Scale bars, 500 µm in overview images (left) 200 µm in magnified images (right). (K) Mature vessel density quantification (*n* = 8 per group). *p < 0.05; **p < 0.01; ***p < 0.001; “ns” means non‐significant difference.

In this study, we established a clinically relevant full‐thickness skin defect (1.5 cm × 1.5 cm) representing approximately 6% total body surface area in nude mice. The critically sized wound model exceeds the spontaneous healing capacity and permits robust evaluation of regenerative therapies (Figure 9A). The wounds were completely covered with blank control (with no treatment), ADF, F‐ADF, or ADM (commercial dressing) (Figure 9A). Wound healing progression was photographically documented on days 0, 4, 7, 10, 14, and 18 post‐intervention, with histological sampling performed on days 7 and 14 (Figure 9A). Quantitative assessment of wound closure rates at day 14 demonstrated statistically significant improvements across all experimental groups relative to untreated controls (control: 81.39 ± 3.45% closure). While the ADM group achieved 86.31 ± 3.33% wound closure (*p* < 0.001), both ADF (96.97 ± 1.48%, *p* < 0.001) and F‐ADF (96.75 ± 2.08%, *p* < 0.001) exhibited near‐complete re‐epithelialization (Figure 9B–D). Comparative analysis revealed the superiority of adipose derived scaffolds over ADM, with ADF showing a 10.66% greater closure rate than ADM (*p* = 0.0032), and F‐ADF demonstrating a 10.47 % improvement (*p* = 0.0020) (Figure 9B–D). Notably, the therapeutic performance between ADF and F‐ADF was statistically equivalent (*p* = 0.7241), suggesting comparable regenerative capacity between these scaffold variants (Figure 9B–D).

Histological evaluation via HE and Masson's trichrome staining at day 7 post‐intervention revealed robust inflammatory cell infiltration across all groups, confirming immune‐mediated initiation of tissue regeneration (Figure 9E,H). Quantitative analysis demonstrated superior re‐epithelialization in treatment groups compared to controls (control: 7.86 ± 0.96 mm, all *p* < 0.001), with ADM showing accelerated epithelial migration (4.25 ± 0.66 mm) and both ADF (3.40 ± 0.78 mm) and F‐ADF (3.58 ± 0.65 mm) achieving optimal advancement (intergroup, *p* = 0.6537). Notably, ADF and F‐ADF supported stratified neoepidermis formation (Figure 9E,F). Granulation tissue development was significantly enhanced in all treatment groups (ADF: 1.41 ± 0.27 mm, F‐ADF: 1.39 ± 0.35 mm, and ADM: 1.33 ± 0.16 mm) compared to controls (1.09 ± 0.17 mm, all *p* < 0.001) (Figure 9G). Masson's staining confirmed enhanced collagen deposition in treatment groups (ADF: 40.34 ± 8.82%, F‐ADF: 41.65 ± 7.12%, and ADM: 21.67 ± 4.83%) compared to controls (14.50 ± 3.34%). ADF and F‐ADF groups uniquely demonstrated early formation of type I collagen bundles (dark blue staining) that correlated with their superior epithelial migration. The results indicated robust regenerative capacity, while ADM lacked comparable mature collagen deposition (Figure 9H,I).

By day 14, the ADF and F‐ADF groups exhibited near‐complete wound closure with complete epidermal restoration (Figure S6A, Supporting Information). The characteristic “basket‐weave” collagen architecture in ADF and F‐ADF groups resembled native skin (Figure S6B, Supporting Information), while ADM showed partial healing with persistent collagen disorganization despite greater granulation tissue thickness (Figure S6A, Supporting Information). The development of distinct rete ridges and dermal papillae at the dermal‐epidermal junction in ADF and F‐ADF groups strongly correlated with Masson's staining patterns, demonstrating physiological tissue regeneration (Figure S6A,B, Supporting Information).

Subsequent immunofluorescence analysis using CD31 for total vessel labeling and α‐SMA/CD31 co‐staining for mature vessel identification (Figure 9J and Figure S6C,D, Supporting Information) revealed that while total vascular density showed no significant differences among treatment groups (ADF: 41.02 ± 3.81 vessels per field, F‐ADF: 42.61 ± 4.28 vessels per field, and ADM: 43.58 ± 4.47 vessels per field; *p* = 0.7579), substantial variations existed in vascular maturation (Figure 9K). The ADF and F‐ADF groups exhibited comparable and superior vessel maturation rates (35.62 ± 1.67 α‐SMA^+^ vessels per field and 35.84 ± 3.42 α‐SMA^+^ vessels per field, respectively, *p* = 0.9563), significantly exceeding both ADM (16.65 ± 5.18 α‐SMA^+^ vessels per field) and control groups (10.40 ± 3.21 α‐SMA^+^ vessels per field) (Figure 9K).

Spatial analysis demonstrated that ADF and F‐ADF promoted uniform vascular distribution throughout the granulation tissue with regular morphology, whereas ADM‐treated wounds showed irregular vessel patterning and decreased maturation despite comparable total vessel numbers. These findings collectively indicate that while all three materials enhanced angiogenesis, ADF and F‐ADF uniquely promoted the development of structurally organized, functionally mature vascular networks, with ADM showing limited capacity for vascular maturation despite its angiogenic potential. The superior vascular patterning achieved by ADF and F‐ADF likely underlies their enhanced wound healing performance, providing optimal perfusion and nutrient delivery to support tissue regeneration.

### 2.10 Trem2^+^ Macrophages and Vascular Endothelial Cells Interactions Promote Angiogenesis Through the VEGF Signaling Pathway

Recent studies highlight the functional diversity of macrophage subsets in tissue repair. Human extracellular matrix promotes wound healing by specifically activating Trem2^+^ macrophages to enhance vascularization, demonstrating superior efficacy compared to ADM [38].

**FIGURE 10:**
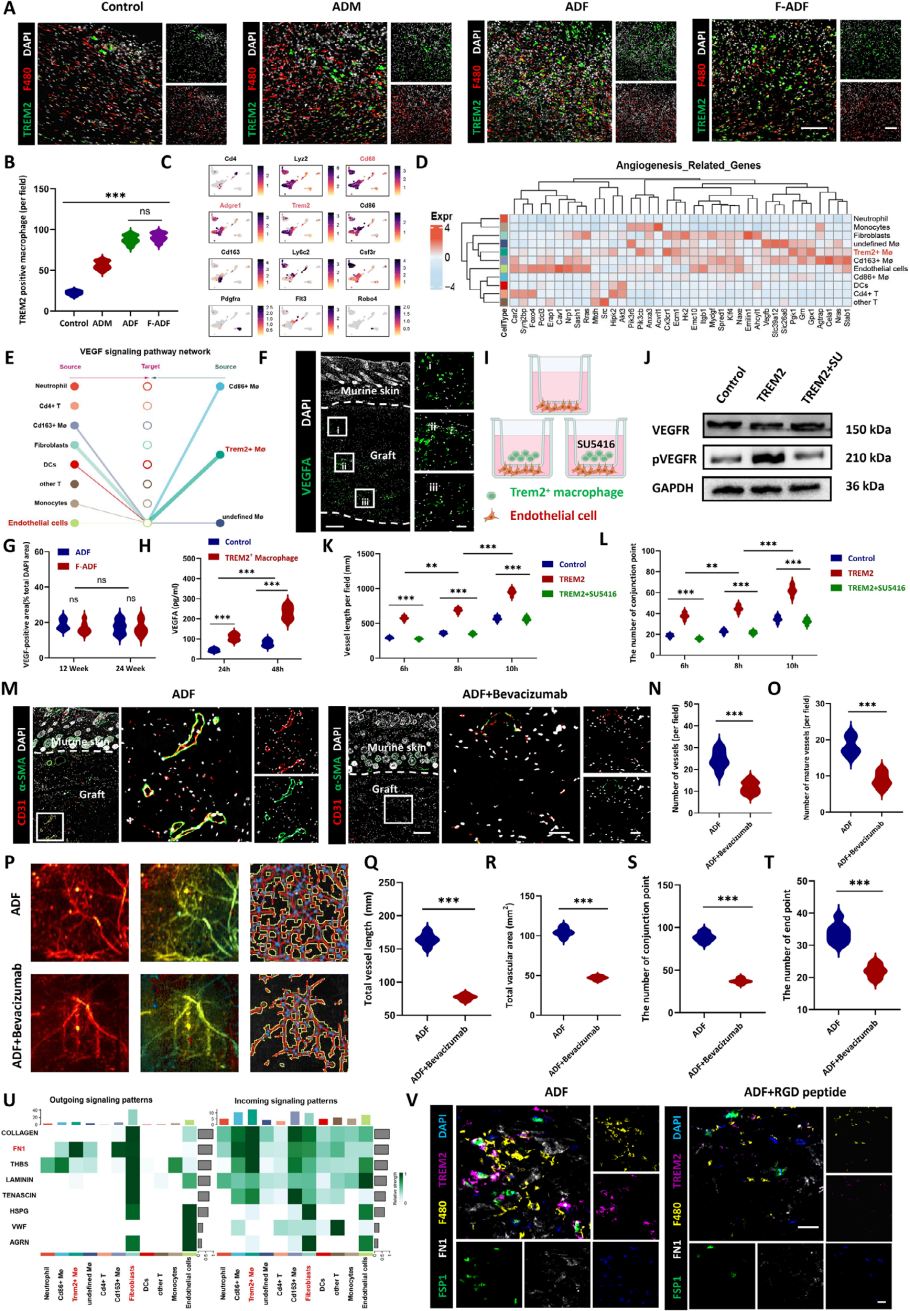
Trem2^+^ macrophages promoted angiogenesis in wound healing and ADF regeneration. (A) Immunofluorescent staining (TREM2) of all groups (Control, ADM, ADF, and F‐ADF) on day 7 of the wound model in nude mice. Scale bar = 100 µm. (B) Quantification of Trem2^+^ macrophages in all groups (Control, ADM, ADF and F‐ADF) on day 7 of the wound model in nude mice (*n* = 8 per group). (C) Feature plots and cluster‐defining markers (Trem2, Adgre1 encoding for F4/80, CD68, and C1qa for Trem2^+^ Macrophages). (D) Angiogenesis‐related genes (Vegfb, Pkg1, and Gpx1) enriched in Trem2^+^ macrophages population. (E) Cell–cell interaction analysis revealing predominant VEGF signaling between Trem2^+^ macrophages and endothelial cells. (F,G) Immunofluorescent staining (VEGFA green) and analysis. Scale bars, 500 µm in overview images (left) 100 µm in magnified images (right). (H) Analysis of VEGFA secretion by Trem2^+^ Macrophages via ELISA. (I) Co‐culture system of Trem2^+^ macrophages and HUVEC with/without VEGF inhibitor SU5416 and (J) Western blot analysis of VEGFR and pVEGFR (*n* = 8 per group). Tube formation assessment of HUVEC, (K) number of conjunction point and (L) total vessel length in co‐culture system of Trem2^+^ macrophages and HUVEC with/without VEGF signaling inhibitor SU5416 (*n* = 8 per group). (F) Immunofluorescent staining (CD31 red and α‐SMA green) of implanted ADF and ADF loaded with VEGF signaling inhibitor Bevacizumab, with overlying murine skin and (N) total vessels and (O) mature vessels analysis. Scale bars, 300 µm in overview images (left) 200 µm in magnified images (right). (P) 3D reconstruction image of vessels for implanted ADF and ADF loaded with VEGF signaling inhibitor Bevacizumab (left: origin 3D image; middle: 3D image with depth information; right: vessels fitting analysis plot) and analysis for (Q) total vessel length, (R) total vascular area, (S) number of conjunction points, and (T) number of end points. (U) FN1‐mediated Trem2^+^ macrophage‐fibroblast interactions. (V) Multiplex immunofluorescence of FSP1^+^ fibroblasts (green), FN1 (white), F4/80^+^ (yellow), and TREM2^+^ (purple) in ADF with/without RGD peptide. *p < 0.05; **p < 0.01; ***p < 0.001; “ns” means non‐significant difference.

To investigate whether ADF, a human ECM‐based bioactive material, shares the same regulatory mechanism, we performed immunofluorescence staining for F4/80 and TREM2 on wound tissues from four experimental groups (Figure 10A,B). Quantitative analysis revealed a significant increase in Trem2^+^ macrophage infiltration across all experimental groups compared to controls (ADF: 87.75 ± 5.42 cells per field, F‐ADF: 90.63 ± 5.24 cells per field, and ADM: 56.38 ± 5.01 cells per field vs. control: 22.38 ± 2.67 cells per field, *p* < 0.001 by one‐way ANOVA). ADF and F‐ADF demonstrated the most robust enhancement (Figure 10A,B), indicative of their superior Trem2^+^ macrophage activating potential. Immunofluorescence profiling further identified predominant Trem2^+^ macrophage accumulation (75.98 ± 7.43%) with minimal co‐localization in dendritic cells (4.79 ± 1.51%), neutrophils (3.10 ± 0.91%), or monocytes (1.84 ± 0.67%) (*p* < 0.001) within implanted ADF (Figure S7A,B, Supporting Information). The results established Trem2^+^ macrophages as the principal effector population in ADF‐mediated tissue regeneration.

To delineate the mechanistic basis of Trem2^+^ macrophage function, we performed re‐analysis of single‐cell RNA sequencing datasets (GSE179745) originating from research on human‐derived ECM modulating wound repair processes [38]. After rigorous quality control, 2114 high‐quality single cells were clustered into 11 distinct populations (Figure S7C, Supporting Information). Macrophages were subclassified into four subsets: Trem2^+^ macrophages (marked by Trem2, Adgre1, CD68, C1qa), CD86^+^ macrophages, CD163^+^ macrophages, and an undefined subset (Figure 10C and Figure S7D, Supporting Information). Functional enrichment analysis revealed that Trem2^+^ macrophages were specifically associated with angiogenesis (Vegf, Pkg1, Gpx1) (Figure S7E, Supporting Information). CellChat analysis identified robust Trem2^+^ macrophage‐endothelial cell crosstalk primarily mediated by VEGF signaling (Figure 10E and Figure S7F,G, Supporting Information), with VEGFA being predominantly expressed in Trem2^+^ macrophages (Figure S7H, Supporting Information). Immunofluorescence confirmed sustained VEGFA expression in ADF/F‐ADF groups at 12 and 24 weeks post‐implantation (Figure 10F,G and Figure S7I, Supporting Information). GFP^+^ cell tracking revealed that ADF and F‐ADF recruited circulating cells expressing VEGFA (ADF: 42.35 ± 7.54%, F‐ADF: 39.08 ± 9.80%, *p* = 0.5315) (Figure 8H,I). While F4/80^+^ VEGFA^+^ co‐staining verified macrophages as the primary source (ADF: 49.48 ± 6.86%, F‐ADF: 48.08 ± 8.16%, *p* = 0.9214) (Figure 8J,K). To delineate the cellular origins of Trem2^+^ macrophages, dual immunofluorescence staining for Trem2 and GFP revealed a subpopulation of Trem2^+^ GFP^+^ positive cells (ADF: 24.18 ± 4.02%, F‐ADF: 24.83 ± 6.16%, *p* = 0.7506) (Figure 8L,M), providing direct evidence that a proportion of Trem2^+^ macrophages were derived from circulating precursors.

Trem2^+^ macrophages represent a specialized subset of lipid‐associated macrophages that play crucial roles in metabolic regulation and tissue homeostasis. Emerging evidence indicates that lipid droplets (LDs), evolutionarily conserved organelles for neutral lipid storage, serve as potent inducers of Trem2 expression in macrophages [39]. Since ADF comes from adipose tissue (the main lipid storage of the body), and our data show it still contains lipids after processing (Oil Red O staining in Figure 1F and SEM in Figure 1K), we hypothesized these leftover lipids might directly activate Trem2^+^ macrophages. Experimental validation confirmed that LDs treatment significantly upregulated Trem2 expression in macrophages (2.12 ± 0.19 fold increase vs. control, *p* < 0.01) (Figure S8A,B, Supporting Information), establishing an effective protocol for partial Trem2^+^ macrophage induction.

Subsequent cytokine profiling by ELISA demonstrated that Trem2^+^ macrophages secreted substantially elevated levels of VEGFA (Trem2^+^: 108.41 ± 16.60 pg/mL and control: 44.01 ± 8.39 pg/mL at 24 h; Trem2^+^: 230.94 ± 33.14 pg/mL and control: 79.03 ± 15.53 pg/mL at 24 h, *p* < 0.001) (Figure 10H). To mechanistically characterize the regulatory effects of Trem2^+^ macrophages on vascular endothelial cells, we established a co‐culture system (Figure 10I). Then, we used the VEGFA inhibitor SU5416 to block VEGF signaling activation, thereby verifying whether Trem2^+^ macrophage‐mediated regulation of endothelial cell function depends on the VEGF pathway. The inhibitory effect of SU5416 on pVEGFR activation was confirmed through western blot (0.22 ± 0.04 fold decrease vs. control, *p* < 0.01) (Figure S8C–E, Supporting Information). Then, we examined VEGF pathway activation in endothelial cells under different co‐culture conditions. The results showed that Trem2^+^ macrophages significantly activated pVEGFR in endothelial cells (2.80 ± 0.22 fold increase vs. control, *p* < 0.01) (Figure 10J and Figure S8F,G, Supporting Information). The effect could be suppressed by SU5416 (Figure 10J and Figure S8F,G, Supporting Information). Tube formation assays demonstrated the tubulogenesis promotion effect of Trem2^+^ macrophages compared to control (vessel length: 517.13 ± 29.19, conjunction: 34.87 ± 2.72), which was partially inhibited by SU5416 (vessel length: Trem2^+^ macrophages: 950.36 ± 2.27, Trem2^+^ macrophages + SU5416: 561.38 ± 29.87; conjunction: Trem2^+^ macrophages: 62.31 ± 4.46, Trem2^+^ macrophages + SU5416: 32.50 ± 2.48, *p* < 0.01) (Figure 10K,L and Figure S8H, Supporting Information). These in vitro results confirm that Trem2^+^ macrophages activate endothelial VEGF signaling via paracrine VEGFA to promote vascularization.

To elucidate the mechanistic contribution of VEGF signaling to ADF‐mediated tissue regeneration, we implemented a targeted pharmacological inhibition approach. Bevacizumab (a clinically approved anti‐VEGFA monoclonal antibody) was administered systemically at 5 mg/kg twice weekly to specifically block VEGF‐dependent pathways (*n* = 8 per group). Integrated multimodal analysis revealed that VEGF blockade profoundly compromised both vascularization and tissue regeneration processes. Immunofluorescence quantification demonstrated significant reductions in total vessel density (ADF + Bevacizumab: 11.75 ± 2.76 vessels per field and ADF: 24.63 ± 4.27 vessels per field, *p* < 0.001) (Figure 10M,N) and mature vessel formation (ADF + Bevacizumab: 8.87 ± 2.03 vessels per field and ADF: 18.25 ± 2.49 vessels per field, *p* < 0.01) (Figure 10O). These vascular impairments were further confirmed by photoacoustic imaging (Figure 10P) showing decreased 3D vascular network complexity (vascular length ADF + Bevacizumab: 77.06 ± 4.11 and ADF: 164.20 ± 8.84; *p* < 0.001) (Figure 10Q), vascular area (ADF + Bevacizumab: 47.07 ± 2.42 and ADF: 104.41 ± 5.43, *p* < 0.001) (Figure 10R), conjunction points (ADF + Bevacizumab: 37.25 ± 2.81 and ADF: 88.75 ± 5.23, *p* < 0.001) (Figure 10S), and end points (ADF + Bevacizumab: 22.05 ± 2.13 and ADF: 33.75 ± 2.91, *p* < 0.001) (Figure 10T). Concomitant with the angiogenic deficits, bevacizumab treatment markedly attenuated adipose tissue regeneration, as evidenced by reduced adipocyte area (ADF + Bevacizumab: 6.22 ± 3.02% and ADF: 27.19 ± 7.79%, *p* < 0.001) (Figure S8I–K, Supporting Information) and diminished perilipin positive area (ADF + Bevacizumab: 5.49 ± 1.18% and ADF: 21.37 ± 2.48%, *p* < 0.01) (Figure S8L,M, Supporting Information). The coordinated impairment of both angiogenesis and adipogenesis following VEGF inhibition establishes VEGFA as a critical mediator of ADF‐driven tissue regeneration.

These in vivo and in vitro experiments collectively demonstrate that ADF and F‐ADF regenerative tissues can recruit cells from the circulatory system and promote their differentiation into Trem2^+^ macrophages, which then interact with vascular endothelial cells via the VEGFA signaling pathway to stimulate angiogenesis and vessel maturation, thereby playing a crucial regulatory role in ADF and F‐ADF regeneration.

### 2.11 Potential Coregulatory Relationships Between Trem2^+^ Macrophages and Other Cells

To characterize the ADF tissue microenvironment, we investigated reciprocal regulation between Trem2^+^ macrophages and other cells. Through systematic analysis of their intercellular communication network, we identified a particularly robust signaling axis with a specific focus on cell clusters modulating Trem2^+^ macrophage function. Intercellular communication analysis revealed a robust FN1‐mediated signaling axis between fibroblasts and Trem2^+^ macrophages (Figure 10U). This observation was consistent with our previous findings that FN1 serves as a pivotal regulatory protein during ADF tissue regeneration and repair (Figure 4G), prompting us to further investigate whether fibroblasts regulate Trem2^+^ macrophage function through the FN1 signaling pathway.

To experimentally validate this hypothesis, we performed a series of spatial and functional analyses. At the tissue level, we conducted dual staining for F4/80 and TREM2 to identify Trem2^+^ macrophages, along with FSP1 labeling for fibroblasts, in implanted ADF, while simultaneously detecting FN1 as a critical molecular marker of intercellular signaling (Figure 10V). Strikingly, immunofluorescence analysis revealed dense FN1 fiber networks surrounding fibroblasts (FSP1^+^), which exhibited substantial colocalization and envelopment relationships with Trem2^+^ macrophages (Figure 10V). To establish the functional significance of these observations, we next employed RGD‐competing peptides to specifically disrupt FN1‐mediated interactions. As predicted, this intervention significantly disrupted FN1 fiber integrity and reduced its expression, accompanied by marked decreases in both FSP1^+^ fibroblast and Trem2^+^ macrophage populations (Figure 10V), thereby confirming the critical role of FN1‐mediated fibroblast‐Trem2^+^ macrophage crosstalk.

To further elucidate the inter‐regulatory mechanisms among macrophage subsets, we systematically analyzed their dynamic interactions and integrated these findings with our previous work on phase‐dependent macrophage polarization during ADF‐mediated tissue regeneration. Through comprehensive cell–cell interaction analyses, we discovered intricate communication networks between Trem2^+^ macrophages and both M1 (CD86^+^) and M2 (CD163^+^) macrophage subsets (Figure S9A, Supporting Information), suggesting potential cross‐regulation among different macrophage populations. To mechanistically dissect Trem2^+^ macrophage function, we established a co‐culture system (Figure S9B, Supporting Information) to evaluate their influence on macrophage polarization. Trem2^+^ macrophage induction was established as mentioned above. Under M1‐polarizing conditions (LPS 100 ng/mL + IFN‐γ 20 ng/mL), qPCR analysis demonstrated that Trem2^+^ macrophages significantly suppressed iNOS expression (LPS + TREM2: 9.75 ± 1.38 fold and LPS: 19.41 ± 2.91 fold compared to controls, *p* < 0.001) (Figure S9C, Supporting Information). Conversely, under M2‐polarizing conditions (IL‐4 20 ng/mL + IL‐13 50 ng/mL), they enhanced Arg1 expression (IL‐4 + TREM2: 44.92 ± 7.03 fold change and IL‐4: 26.95 ± 5.83 fold change compared to controls, *p* < 0.001) (Figure S9D, Supporting Information).

This bidirectional regulatory capacity was further confirmed at the mRNA level by qPCR. Trem2^+^ macrophages inhibited TNFα expression (LPS + TREM2: 8.02 ± 1.25 fold change and LPS: 16.08 ± 2.29 fold change compared to controls, *p* < 0.001) (Figure S9E, Supporting Information) while simultaneously promoting IL‐10 expression (IL‐4 + TREM2: 20.99 ± 2.91 fold change and IL‐4: 13.34 ± 2.51 fold change compared to controls, *p* < 0.001) (Figure S9F, Supporting Information). These quantitative findings demonstrate that Trem2^+^ macrophages possess a unique immunomodulatory capacity to attenuate pro‐inflammatory M1 responses while enhancing anti‐inflammatory M2 polarization.

Collectively, our results identify FN1‐mediated fibroblast‐Trem2^+^ macrophage crosstalk as a pivotal regulatory mechanism in ADF‐driven tissue regeneration. The findings not only characterize this cellular communication axis but also underscore the need to investigate the participating fibroblast subsets and their temporal control of macrophage polarization during tissue repair progression. The study provides fundamental mechanistic insights into the dynamic macrophage polarization and cytokine profile changes following ADF implantation. Beyond elucidating the pleiotropic functions of Trem2^+^ macrophages, these data reveal the remarkable sophistication of intercellular signaling networks that coordinate regenerative processes, establishing a novel paradigm for understanding and potentially manipulating tissue repair mechanisms.

## 3 Discussions

The field of ECM‐based regenerative medicine has made significant strides in decellularization technologies, establishing multimodal approaches that integrate physical (freeze–thaw cycles, sonication), chemical (detergents, acid/base treatments), and enzymatic methods to achieve complete cell removal with residual DNA content below 50 ng/mg tissue [40, 41]. These advanced techniques not only effectively eliminate cellular components but also preserve critical ECM structural proteins (collagens, fibronectin) and bioactive molecules that regulate cellular behavior and tissue regeneration [42, 43, 44]. The development of tissue‐specific protocols, particularly perfusion decellularization, has enabled the successful generation of whole‐organ scaffolds with intact vascular networks, representing a major breakthrough in complex tissue engineering [45, 46]. Concurrently, the emergence of eco‐friendly alternatives such as supercritical CO_2_ and enzymatic methods addresses the limitations of conventional detergents by minimizing ECM damage while maintaining decellularization efficacy [47, 48].

Despite these advancements, the field continues to grapple with several interconnected challenges [49]. A fundamental paradox persists between achieving complete cellular removal and preserving essential bioactive components, as the rigorous processing conditions required for thorough decellularization often degrade critical growth factors and signaling molecules [50]. This balance is particularly difficult to achieve in elastic tissues, where the complex architecture makes complete cellular elimination especially challenging [51]. Additional limitations include persistent immunogenicity risks from residual cellular material in allogeneic applications, structural alterations that compromise cellular recognition and scaffold remodeling capacity, and the critical shortage of human tissue sources that restricts clinical scalability [49]. From a technical standpoint, large‐scale implementation faces substantial barriers. Perfusion decellularization, while effective for whole‐organ scaffolds, demands specialized equipment and time‐intensive protocols [45, 46]. Moreover, maintaining batch‐to‐batch consistency in tissue‐specific decellularization remains an inherent challenge, further complicating standardization and regulatory approval [49]. Developing alternative approaches that reconcile decellularization efficacy with ECM biofunctional preservation represents a critical pathway for advancing ECM‐based therapies into clinical practice.

Our innovative adipose‐derived matrix film (ADF) technology represents a paradigm shift in this field, employing a gentle physical processing technique inspired by traditional “papermaking” methods that effectively transforms clinically discarded adipose tissue into a ready‐to‐use bio‐membrane. Compared to existing decellularized ECM products like ADM, ADF offers distinct advantages: (1) elimination of immune rejection risks through autologous sourcing, (2) preservation of tissue‐specific bioactive components through gentle physical processing, (3) capacity for long‐term cryopreservation without functional compromise, and (4) abundant and diverse tissue sources with streamlined and efficient processing. Additionally, ADF holds particular significance when contextualized within current clinical applications of autologous adipose tissue, which remain largely confined to injectable formulations [20, 52, 53, 54, 55]. The development of ADF's unique membrane morphology constitutes a major conceptual and practical advancement in adipose tissue engineering, effectively overcoming the inherent limitations of particulate injectable systems while preserving the therapeutic benefits of autologous ECM. This novel physical form factor enables previously unattainable clinical applications that leverage both the biological properties of native adipose ECM and the practical advantages of membrane‐based delivery systems. ADF demonstrates optimal mechanical properties pre‐/post‐implantation, with suitable tensile strength and strain capacity ensuring surgical handling. Its stable regeneration maintains volume retention for long‐term durability, while controlled surface roughness prevents displacement after implantation. ADF demonstrates versatile clinical applications through its unique combination of mechanical and regenerative properties (Figure S10A, Supporting Information).

In wound management, its optimal strain capacity and tensile strength prove particularly valuable for treating complex chronic wounds such as diabetic foot ulcers, where the material dynamically accommodates wound contraction while maintaining structural integrity [56]. The same properties enable precise anatomical adaptation across a spectrum of reconstructive applications, including post‐mastectomy breast reconstruction [57]. ADF may enhance implant outcomes by improving contouring, tactile properties, and reducing capsular contracture [58]. It is also suitable for facial reconstruction after trauma/tumor resection, and craniofacial/gingival defect repair [59]. In aesthetic surgery, ADF effectively wraps nasal/auricular implants [60, 61], as an advanced biological membrane (Figure S10B, Supporting Information). It may prevent visibility/translucency while minimizing chronic implant‐skin inflammatory reactions, thereby reducing risks of skin breakdown and implant exposure [60, 61]. For mechanically demanding scenarios, an innovative “*sandwich*” composite design integrates ADF with clinically approved mesh materials [62], showing particular promise in complex abdominal wall reconstruction (Figure S10C, Supporting Information). Additionally, ADF can be processed into injectable formulations for facial volumization and aesthetic enhancement [63]. These diverse applications maintain ADF's inherent regenerative advantages while providing novel solutions for both elective and trauma‐related surgical interventions.

Through comprehensive proteomic analysis, we have demonstrated that ADF maintains exceptional preservation of native ECM components, with 418 identified proteins showing remarkable stability and only 24 exhibiting differential expression following 12 months of cryopreservation. This preservation capability not only validates the efficacy of our processing method but also enables the novel clinical concept of an “*ECM bank*” (Figure S10D, Supporting Information). It allows for the storage of a patient's own adipose ECM during periods of health for future regenerative applications, including potential interventions against age‐related tissue degeneration. The autologous nature of ADF completely eliminates risks of immune rejection while preserving the full complement of tissue‐specific biochemical and biomechanical cues essential for proper tissue regeneration [64, 65].

At the cellular and molecular level, ADF orchestrates tissue regeneration through an exquisitely coordinated multicomponent mechanism. The investigations reveal that during the well‐regulated degradation and regeneration processes of ADF, dynamic interactions occur between recruited circulating cells (predominantly macrophages) and ECM components. The specific activation of Trem2^+^ macrophages emerge as the central regulatory node in both implantation and wound healing models. The specialized subpopulation serves as a critical nexus for angiogenesis and tissue remodeling. Comprehensive in vitro and in vivo studies demonstrate that Trem2^+^ macrophages significantly enhance angiogenesis through paracrine VEGFA‐mediated regulation of endothelial cell function. The pro‐regenerative activity appears to be further modulated via potential FN1‐mediated crosstalk with fibroblasts and could be prevented by RGD peptide interference. Notably, our finding that Trem2^+^ macrophages are specifically activated by adipose‐derived lipid droplets suggests that controlled retention of lipid components may exert beneficial modulatory effects on tissue regeneration. The finding offers a novel perspective contrasting with prior reports that excessive lipid release from ruptured adipocytes triggers chronic inflammation and oil cyst formation in fat grafts, while optimal lipid retention thresholds need further validation. ADF also demonstrates precise temporal regulation of immune responses, facilitating a coordinated phenotypic transition from initial pro‐inflammatory (M1) to subsequent pro‐regenerative (M2) states, thereby creating an optimal microenvironment for tissue repair. Cellular interaction analyses and in vitro experiments show that Trem2^+^ macrophages play a pivotal regulatory role in macrophage phenotypic switching. When synergistically combined with its demonstrated capacity to promote the formation of mature, functional vascular networks, these properties collectively account for ADF's superior clinical performance compared to existing ADM products. These findings provide novel therapeutic targets and mechanistic insights for ECM‐mediated tissue regeneration.

While establishing ADF as a breakthrough platform in regenerative medicine, several critical limitations require consideration alongside promising research directions. The current nude mouse models, though valuable for initial human‐derived ADF evaluation, fail to fully replicate clinical immune cells‐ECM interactions, particularly regarding adaptive immunity's underexplored role in tissue regeneration [66, 67]. Beyond the well‐characterized innate immune responses, emerging evidence reveals that adaptive immune cells, including specialized T lymphocytes and B lymphocytes, orchestrate regeneration through multifaceted mechanisms [68, 69, 70]. The bidirectional crosstalk between adaptive immunity and ECM scaffolds presents particular clinical relevance for ADF therapy. ECM degradation products may shape T lymphocyte responses, while T lymphocyte cytokines conversely regulate macrophage‐mediated remodeling [71], interactions potentially modified by ADF's residual lipids and ECM epitopes. Current nude mouse models cannot assess these mechanisms, though single‐cell sequencing in immunocompetent systems reveals macrophages as central regulators within complex immune networks. In our experimental design, we tried to prepare ADF using adipose tissue from C57BL/6 mice to facilitate autologous transplantation studies under physiologically intact immune conditions. However, comparative analysis revealed substantial interspecies variations between murine‐derived and human‐derived ADF preparations. Most notably, murine‐derived ADF exhibited significantly higher retention of cellular nuclear material (Figure S10E, Supporting Information), a characteristic that could potentially induce pronounced inflammatory responses and compromise the faithful recapitulation of the immune remodeling effects characteristic of human‐derived ADF. More physiologically relevant models would likely demonstrate greater complexity, including enhanced inflammatory initiation, deeper adaptive immune involvement, and modified macrophage dynamics.

The in vivo transplantation experiments demonstrated that Trem2^+^ macrophages regulate endothelial function via VEGFA, supported by observed inhibition of angiogenesis and tissue regeneration upon bevacizumab administration. While this approach lacks cellular specificity, as bevacizumab blocks the activation of all cellular sources of VEGFA. Although in vitro studies confirmed the paracrine VEGF‐mediated pro‐angiogenic effects of Trem2^+^ macrophages. Furthermore, the use of RGD peptide to disrupt FN1‐mediated fibroblast‐Trem2^+^ macrophage interactions requires cautious interpretation. Given ADF's ECM‐rich composition, RGD peptides may non‐specifically inhibit both FN1‐dependent crosstalk and direct ADF‐fibroblast interactions, potentially causing broad suppression of cellular activation and intercellular communication. Critically, due to the inherent heterogeneity of fibroblast subpopulations, identifying the specific subtypes that regulate Trem2^+^ macrophages is essential for elucidating ADF's regenerative mechanisms.

Future studies should prioritize humanized mouse models [72] integrated with single‐cell sequence technologies to systematically delineate the spatiotemporal dynamics of cell‐ECM interactions, particularly involving adaptive immune components like lymphocytes, and to decipher the precise immunoregulatory mechanisms during ADF remodeling. Complementary approaches employing cell‐specific knockout models [73] and in vitro experiments should be implemented to validate the regulatory roles of key cellular players. Such a multidimensional research strategy will unlock ADF's full regenerative potential by orchestrating scaffold‐immune system crosstalk while mitigating fibrotic responses, thereby accelerating clinical translation of this cutting‐edge technology.

The papermaking‐inspired ADF fabrication method demonstrates notable advantages in operational simplicity and cost‐effectiveness. As a minimally manipulated human tissue product, autologous ADF qualifies for regulatory exemption under 21 CFR 1271.3 (FDA) by meeting three fundamental criteria: homologous use, standalone application, and localized effect. This relatively lenient regulatory framework facilitates rapid clinical translation in aesthetic medicine and small‐scale tissue repair through optimization of point‐of‐care processing systems and donor screening protocols. However, several technical challenges must be addressed during translation: Standardized operating procedures are required to mitigate inherent variability arising from inter‐patient adipose tissue heterogeneity, while sterilization processes need optimization to ensure complete pathogen eradication without compromising ECM structural integrity and bioactivity. Although the autologous nature circumvents immunogenicity concerns, it inherently restricts ADF to personalized therapies.

Allogeneic strategies utilizing decellularized adipose tissue could expand clinical applicability but face more stringent regulatory requirements as biological products [74, 75]. The involvement of inter‐donor transplantation triggers mandatory IND submissions and GMP certification, with particular emphasis on decellularization validation (residual DNA < 50 ng/mg) and immunogenicity control (anti‐HLA antibody detection). This regulatory divergence results in substantially higher development costs and prolonged translation timelines for allogeneic ADF. Furthermore, current evidence indicates that intensified processing protocols necessary for immunogenic safety may compromise ECM ultrastructure, presenting an unresolved efficacy safety trade‐off [5, 8, 76].

Moving forward, advancing ADF toward clinical implementation requires addressing several key priorities. More physiologically relevant animal models that better replicate human immune responses and healing processes must be developed to bridge the current translational gap. Concurrently, large‐scale manufacturing protocols need optimization to ensure consistent product quality and performance. Targeted clinical trials should be initiated to evaluate ADF's efficacy in specific indications such as chronic wounds and breast reconstruction, while combinatorial approaches incorporating mesenchymal stem cells or growth factor cocktails should be explored to enhance therapeutic potential.

From an industrial perspective, a dual‐track development paradigm is emerging. Autologous ADF maintains dominance in emergency and small‐scale repairs through innovations like intraoperative processing devices, while allogeneic ADF focuses on large‐area trauma and burns via shared donor banks and automated processing systems. FDA's “Same Surgical Procedure” exemption creates new pathways for autologous products, while the “Regenerative Medicine Advanced Therapy” designation accelerates allogeneic product approval. Critical future developments include establishing cross‐institutional networks for donor resource integration and quality standardization, alongside next‐generation immunomodulatory technologies to achieve complementary advantages. This translational process necessitates multidisciplinary collaboration among academia, industry, and clinicians to bridge the gap between laboratory research and standardized clinical implementation in regenerative medicine.

ADF research has revealed systemic dimensions of ECM‐mediated regeneration that challenge traditional paradigms focused solely on local cellular responses. Our findings demonstrate that circulating cell populations, particularly monocyte‐derived macrophages, serve as pivotal regulators during ADF remodeling and tissue regeneration. The discovery suggests that optimal therapeutic outcomes may require coordinated modulation of both local and systemic factors, opening new avenues for developing innovative treatment strategies, including combination therapies, to maximize the clinical potential of ECM‐based regenerative approaches. The integration of these translational and basic science insights will be crucial for fully realizing ADF's promise in clinical practice.

## 4 Conclusion

In summary, this comprehensive body of work establishes ADF as a transformative platform in regenerative medicine that successfully addresses multiple longstanding challenges in the field. By elucidating the dynamic interplay between ECM components, specialized macrophage populations, and stromal cells, we have not only developed a clinically applicable solution but also advanced fundamental understanding of ECM‐guided tissue repair mechanisms. The development of effective cryopreservation methods and innovative composite material strategies further enhances the practical utility and versatility of this technology. These scientific and technological advances, bridging fundamental matrix biology with unmet clinical needs, represent a significant leap forward toward realizing the potential of personalized, cell‐free regenerative therapies that could revolutionize treatment paradigms across multiple medical specialties, including wound care, reconstructive surgery, and potentially cardiovascular and musculoskeletal repair. Future research addressing the identified limitations and opportunities will be crucial for translating these promising laboratory findings into widespread clinical application and maximizing the therapeutic impact of ADF technology.

## 5 Materials and Methods

The Materials and Methods section can be found in the Supplementary Materials.

## Author Contributions

**Mengmeng Hou**: writing – review and editing, writing – original draft, validation, methodology, investigation, formal analysis, data curation, conceptualization. **Nini Shi**: writing – review and editing, writing – original draft, validation, methodology, formal analysis, data curation. **Yajie Guo**: writing – review and editing, writing – original draft, validation, methodology, formal analysis, data curation. **Jiezhang Tang**: writing – review and editing, validation, resources, methodology, formal analysis. **Han Peng**: writing – review and editing, validation, resources, methodology, investigation, formal analysis, data curation. **Baoyan Liang**: writing – review and editing, validation, resources, methodology, investigation. **Yixuan Yu**: writing – review and editing, validation, resources, methodology. **Chenggang Yi**: writing – review and editing, validation, resources, methodology, formal analysis. **Huichen Li**: writing – review and editing, writing – original draft, validation, supervision, methodology, funding acquisition, formal analysis, data curation, conceptualization.

## Ethics Statement

All animal experiments were approved by the Ethics Committee of the Fourth Military Medical University (No. IACUC‐20240726). Human tissue collection involved in this research were approved by the Ethics Committee of the Fourth Military Medical University (No. KY20243548‐1).

## Conflicts of Interest

The authors declare no conflicts of interest.

## Supporting information




**Supporting File 1**: exp270136‐sup‐0001‐SuppMat.doc.

**Supporting File 2**: exp270136‐sup‐0002‐Video1.mp4.

**Supporting File 3**: exp270136‐sup‐0003‐Video2.mp4.

## Data Availability

The data that support the findings of this study are available from the corresponding author upon reasonable request.
