# Supplementary material for: Autologous Extracellular Matrix‐Based Cell‐Free Therapy for Tissue Regeneration Through Trem2+ Macrophages Mediated Angiogenesis
Source: Exploration (Beijing). 2026 Feb 18;6(2):20250031. doi: 10.1002/EXP.20250031 (PMC13094522; doi:10.1002/EXP.20250031)
Supplement: Supplementary file 1 — Supporting File 1: exp270136‐sup‐0001‐SuppMat.doc. [file EXP2-6-20250031-s001.doc]

**Supplementary materials**

**Supplementary FIGURE 1.** Preparation and characterization of the ADF. (A,B) The processing of ADF from adipose tissue obtained through abdominal wall surgery. (C) Quantification of α-Gal. (D) Digital images of the ADF under different operations (bend, twist, and stretch). (E) Tensile stress-strain of ADF at 37 °C and humidity. Tensile stress-strain of ADM at (F) room temperature and (G) 37 °C and humidity. (H) Quantification of volume change of ADF under 37 °C and humidity for 2 h. (I) 3D reconstruction image of F-ADF after laser confocal microscopy scanning. (J) Image of ADF after laser confocal microscopy scanning. (K) Images of ADM immersed in physiological saline at different times. (L) Swelling ratio (weight) assessment of ADF and F-ADF immersed in physiological saline at different times.*p < 0.05; **p < 0.01; ***p < 0.001; “ns” means non-significant difference.


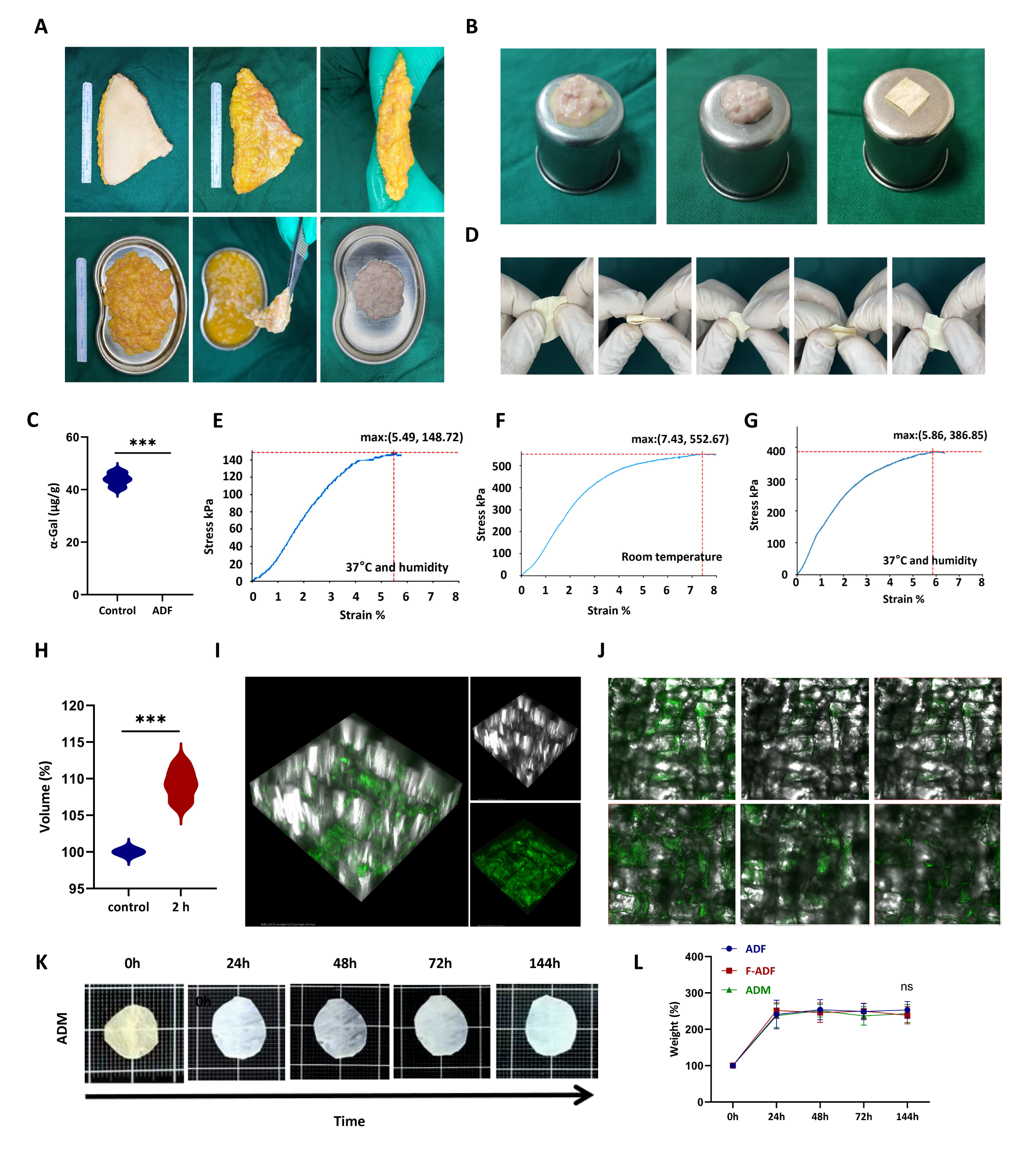


**Supplementary FIGURE 2.** In vitro biocompatibility and in vivo safety assessment of ADF.

Representative fluorescence images of Calcein-AM/PI/DAPI staining (green: viable cells; red: nonviable cells; blue: nuclei) in (A) ADSCs, (C) HUVEC and (E) HDF after being cultured with ADF extracts for 24 h and cell viabilitiy of (B) ADSCs, (D) HUVEC and (F) HDF. Assessment of red blood cells number (G) and (H) white blood cells number at 4 and 12 weeks post-implantation. Serum BUN (I) and CRE (J) detection at 4 and 12 weeks post-implantation (n = 8 per group). *p < 0.05; **p < 0.01; ***p < 0.001; “ns” means non-significant difference.


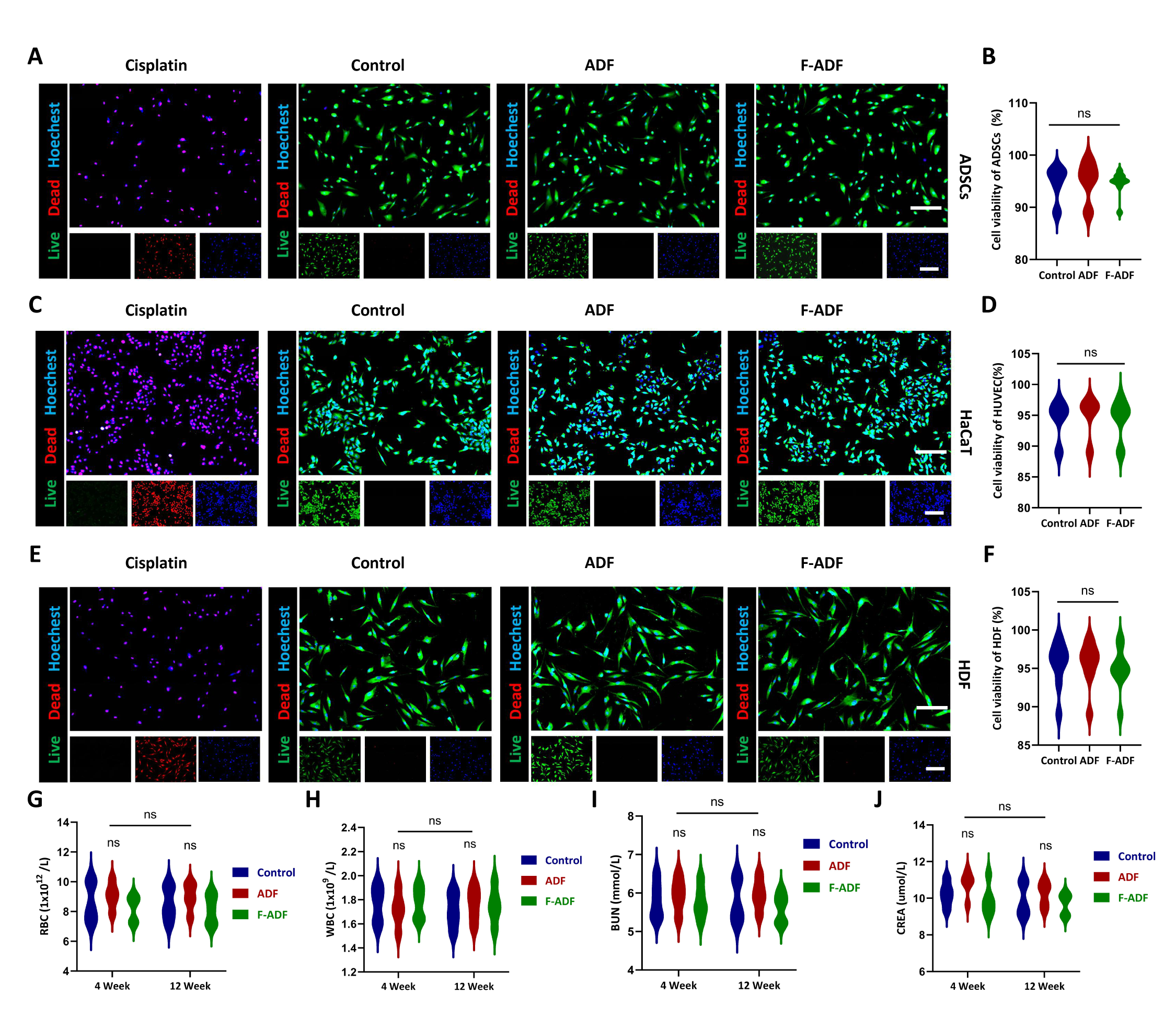


**Supplementary FIGURE 3.** Functional regulation of ADF on HDF and HaCaT cells. (A,B) Cell proliferation of HDF after being cultured with ADF and F-ADF extracts for 24 h with EdU assay (n = 8 per group). Scale bar = 100 μm. (C,D) Cell migration of HDF after being cultured with ADF and F-ADF extracts for 24 h with Transwell assay (n = 8 per group). Scale bar = 100 μm. (E,F) Cell proliferation of HaCaT after being cultured with ADF and F-ADF extracts for 24 h with EdU assay (n = 8 per group). Scale bar = 100 μm. (G,H) Cell migration of HaCaT after being cultured with ADF and F-ADF extracts for 24 h with Transwell assay (n = 8 per group). Scale bar = 100 μm. (I) CCK-8 assay of HDF after being cultured with ADF and F-ADF extracts for 24 h, 48 h and 72 h (n = 8 per group). (J) CCK-8 assay of HaCaT cells after being cultured with ADF and F-ADF extracts for 24 h, 48 h and 72 h (n = 8 per group). *p < 0.05; **p < 0.01; ***p < 0.001; “ns” means non-significant difference.


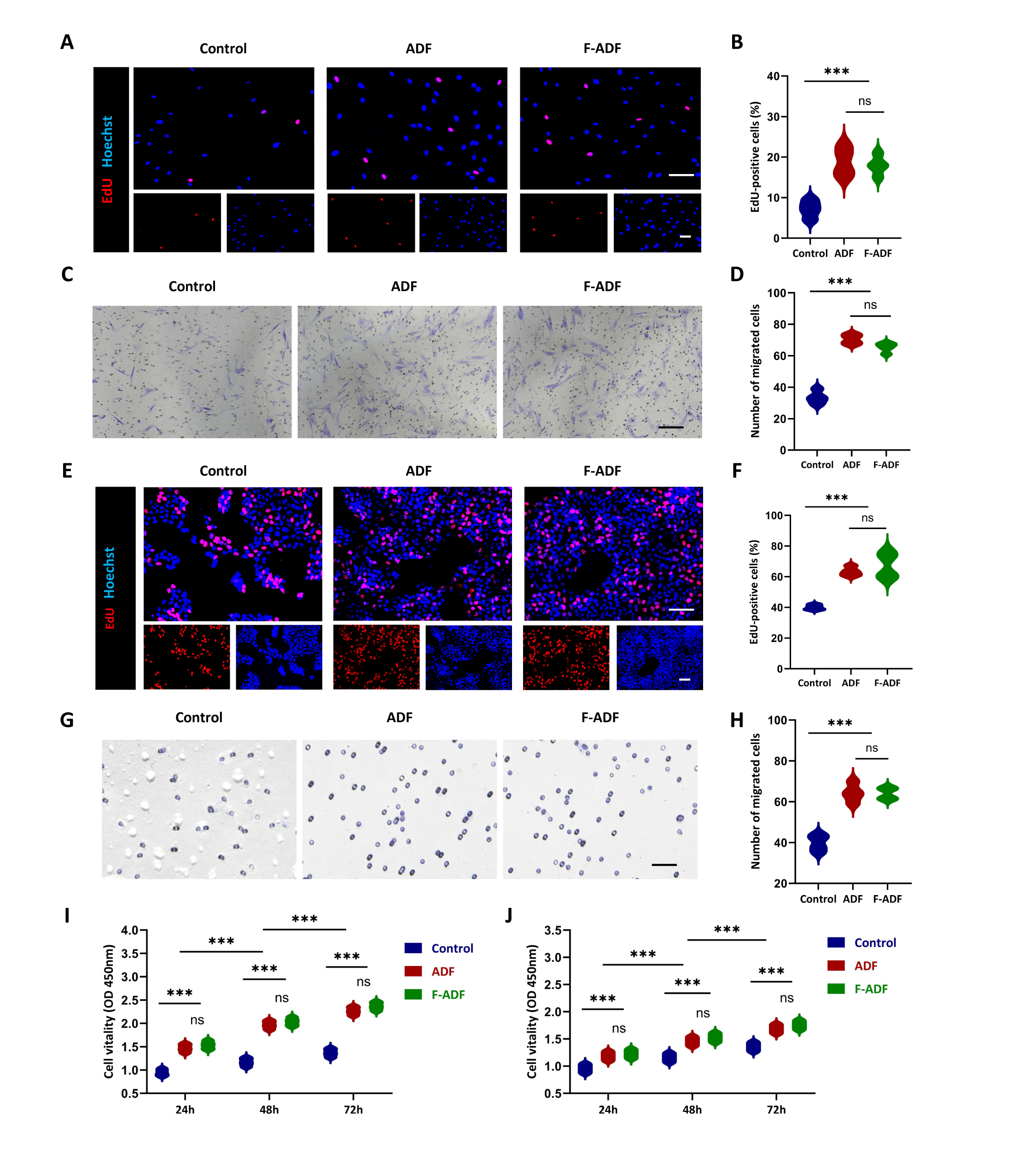


**Supplementary FIGURE 4.** Cell infiltration-mediated angiogenesis plays a crucial role in ADF regeneration and remodeling. (A) Digital images of ADF and F-ADF at 4 and 12 weeks post-implantation. (B) Tensile stress-strain of ADF post-implantation at 37 °C and humidity. IL-6 (C) and IL-10 (D) detection of ADF and F-ADF at 2, 4 weeks post-implantation using ELISA. (E) Cellular infiltration into ADF/F-ADF implants at 4, 12 weeks post-implantation. (F) Immunofluorescent staining (CD31 red and α-SMA green) of ADF and F-ADF post-implantation with overlying murine skin. Scale bars, 300 μm in overview images (left) 200 μm in magnified images (right). Dotted lines: implant-host tissue interface. (G) Immunofluorescent staining (CD31 red and α-SMA green) and (H) F4/80 of nude mice skin. Scale bar = 100 μm. (I) Immunofluorescent staining (CD14 for monocytes, CD19 for T cells and CD3 for B cells) of ADF and F-ADF post-implantation with overlying murine skin. Scale bar = 300 μm. Dotted lines: implant-host tissue interface.*p < 0.05; **p < 0.01; ***p < 0.001; “ns” means non-significant difference.


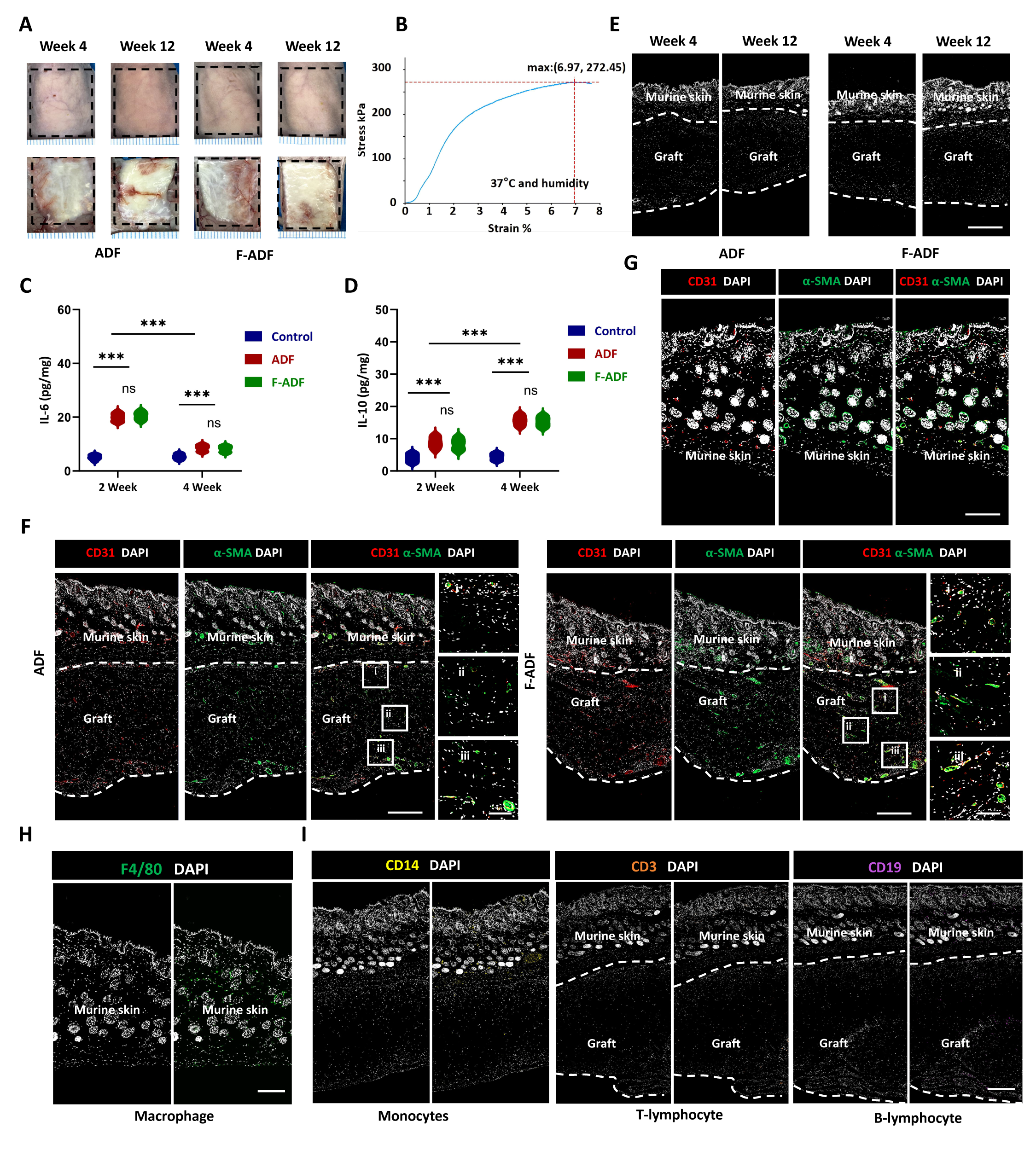


**Supplementary FIGURE 5.** M1 and M2 macrophage in ADF regeneration and remodeling. (A) Immunofluorescent staining (CD206 red and F4/80 green) of ADF/F-ADF post-implantation with overlying murine skin and (C) analysis. Scale bars, 300 μm in overview images (left) 200 μm in magnified images (right). Dotted lines: implant-host tissue interface. (B) Immunofluorescent staining (CD206 red and F4/80 green) of nude mice skin. Scale bar = 100 μm. (D) Immunofluorescent staining (CD206 red and iNOS green) of ADF and F-ADF post-implantation and (E) analysis. qPCR analysis of mRNA level of iNOS (F) and Arg1 (G), and iNOS/Arg1 ratio (H).*p < 0.05; **p < 0.01; ***p < 0.001; “ns” means non-significant difference.


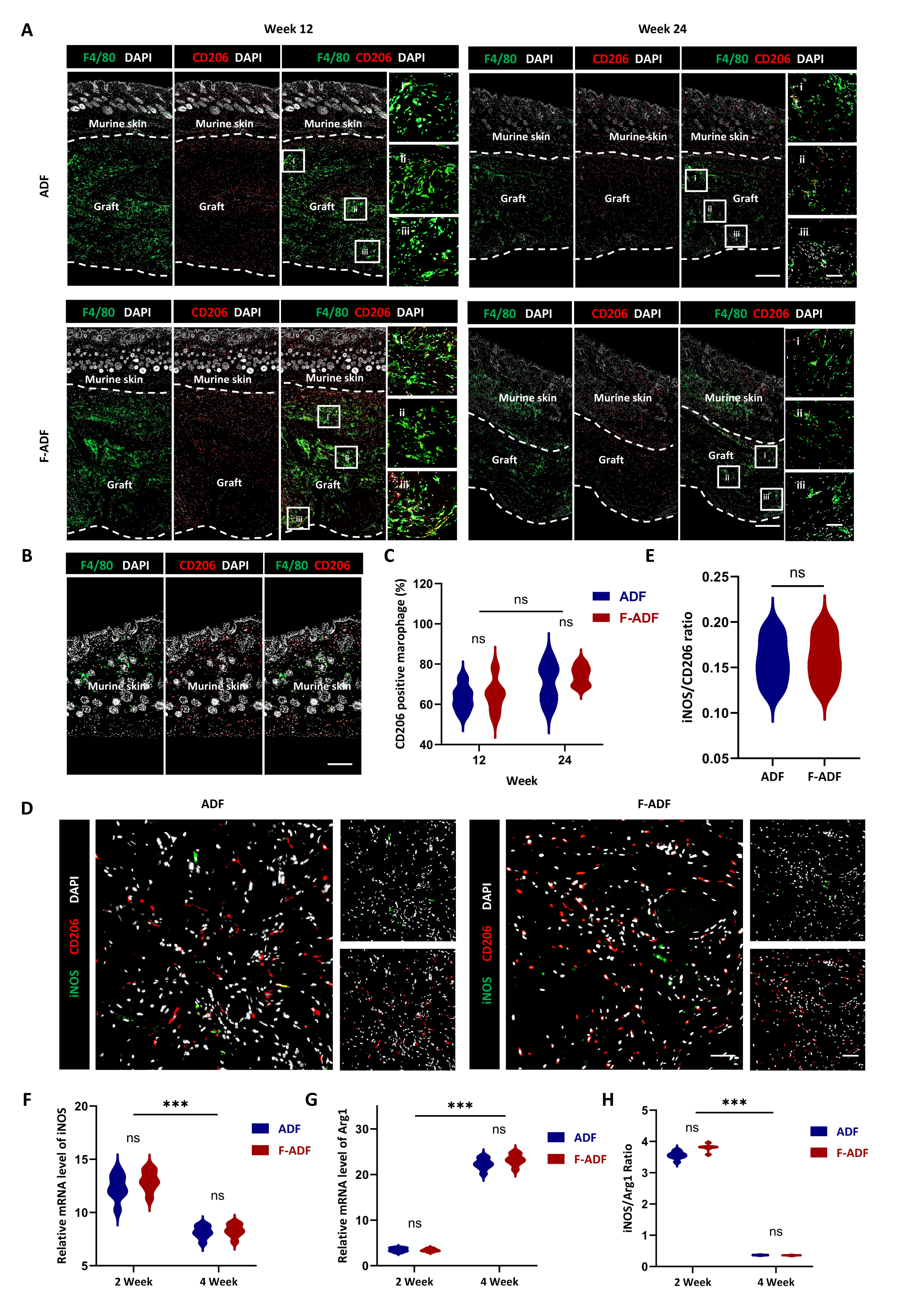


**Supplementary FIGURE 6.** Treatment efficacy of ADF in a nude mouse wound model. (A) HE and (B) Masson staining of skin tissue in all groups on day 14. Scale bars, 500 μm in overview images (left), 200 μm in magnified images (right). (C) Immunofluorescent staining (CD31 red and α-SMA green) of all groups on day 7. Scale bars = 100 μm. (D) Analysis of total vessels of all groups (n = 8 per group).*p < 0.05; **p < 0.01; ***p < 0.001; “ns” means non-significant difference.


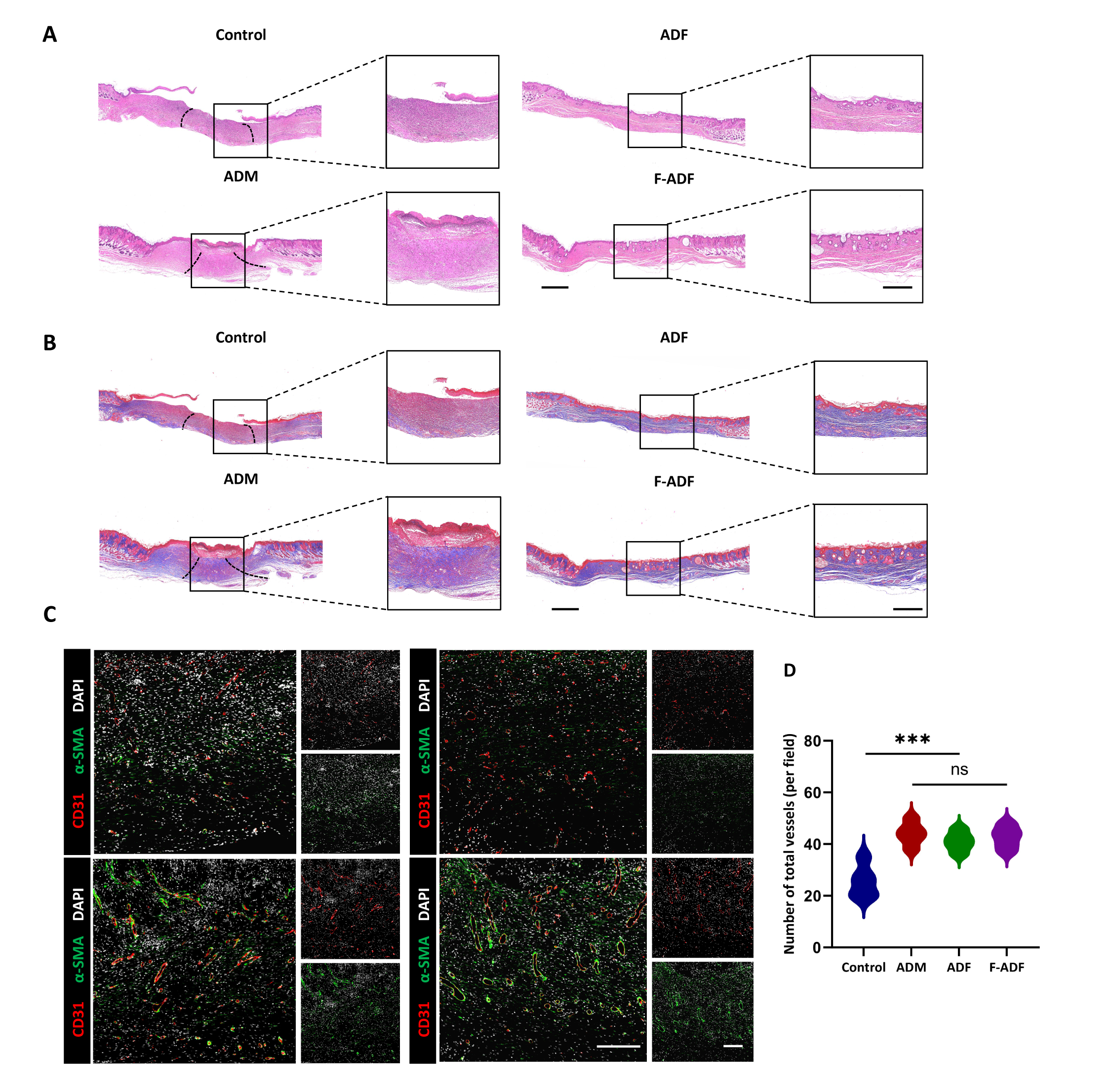


**Supplementary FIGURE 7.** Trem2^+^ macrophages and vascular endothelial cells interactions promote angiogenesis through the VEGF signaling pathway. (A,B) Immunofluorescent staining (TREM2) of ADF and F-ADF post-implantation with overlying murine skin and analysis (F4/80 for macrophages, CD11c for dendritic cells, Ly6G for neutrophils and CD14 for monocytes). Scale bars, 500 μm in(left) and 100 μm in (right and middle). Dotted lines: implant-host tissue interface. (C) Unsupervised clustering analysis showed distinct 11 cell types. (D) Cluster-defining markers (Trem2, Adgre1 encoding for F4/80, CD68, and C1qa for Trem2^+^ Macrophages). (E) The functional enrichment analysis of Trem2^+^ macrophages population involved in the angiogenesis pathway. (F,G) Trem2^+^ macrophages exhibited the highest interactions with other lineages for VEGF signaling. (H) Vegfa exhibits the predominance expression in Trem2^+^ macrophages cell population. (I) Immunofluorescent staining (VEGFA green). Scale bars, 500 μm in overview images (left) 100 μm in magnified images (right). *p < 0.05; **p < 0.01; ***p < 0.001; “ns” means non-significant difference.


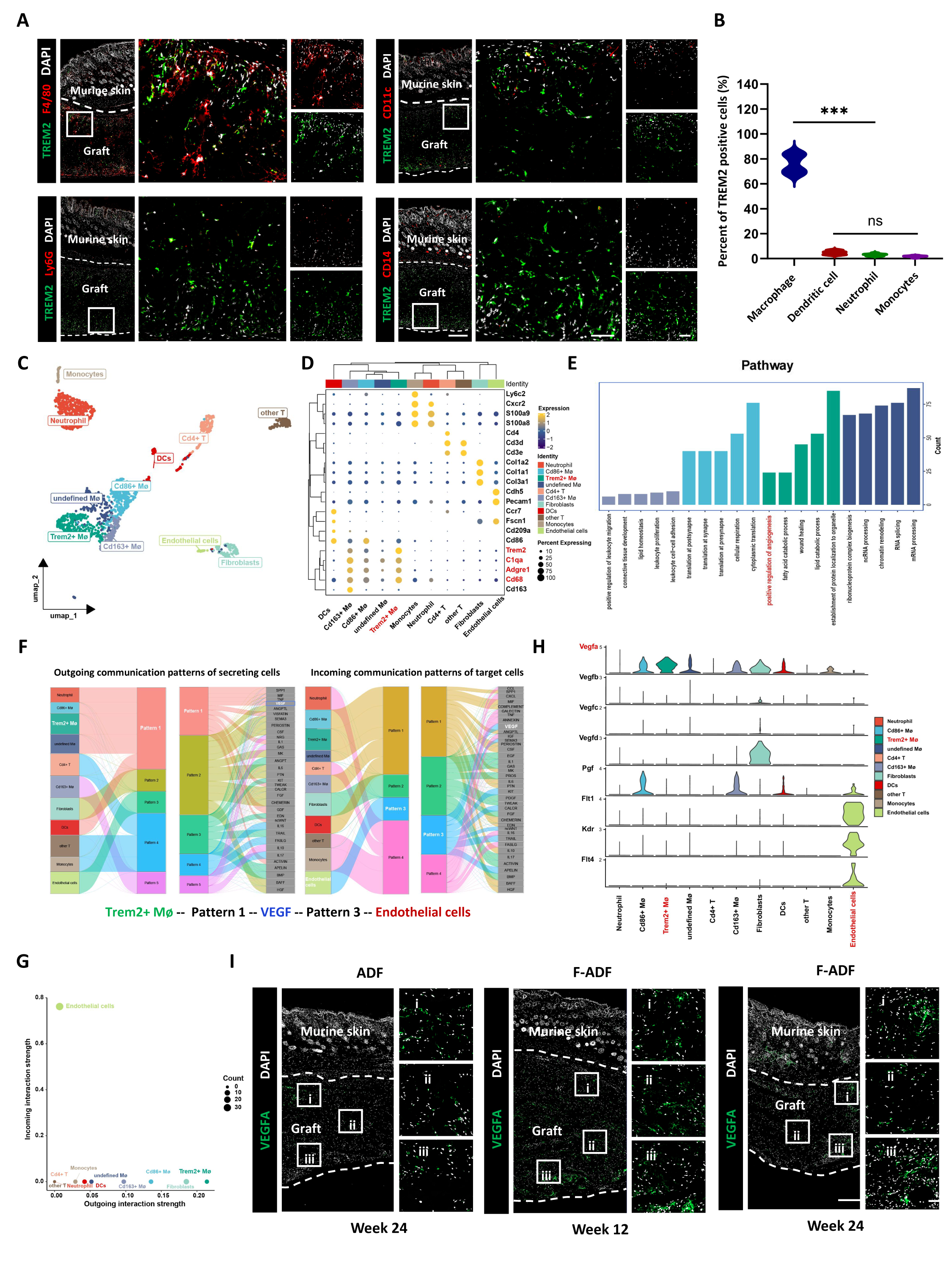


**Supplementary FIGURE 8.** VEGF signaling pathway is essential for ADF regeneration and Trem2^+^ macrophages interactions with endothelial cells. (A) Western blot analysis of TREM2 with the Liquid drops adding in macrophage culture (n = 8 per group) and (B) analysis. (C) Western blot analysis of VEGFR and pVEGFR with/without VEGF inhibitor SU5416 (n = 8 per group) and (D,E) analysis. (F,G) Western blot analysis of VEGFR and pVEGFR in co-culture system of Trem2^+^ macrophages and HUVEC with/without VEGF inhibitor SU5416. (H) Tube formation images. HE staining (I) and Masson’s trichrome staining (J) of ADF post-implantation and ADF loaded with VEGF signaling inhibitor Bevacizumab. Scale bar = 300 μm. (K) Quantification of adipose tissue regeneration volume (%) (L,M) Immunofluorescent staining (perilipin green) of ADF post-implantation and ADF loaded with VEGF signaling inhibitor Bevacizumab and analysis. Scale bar = 100 μm. *p < 0.05; **p < 0.01; ***p < 0.001; “ns” means non-significant difference.


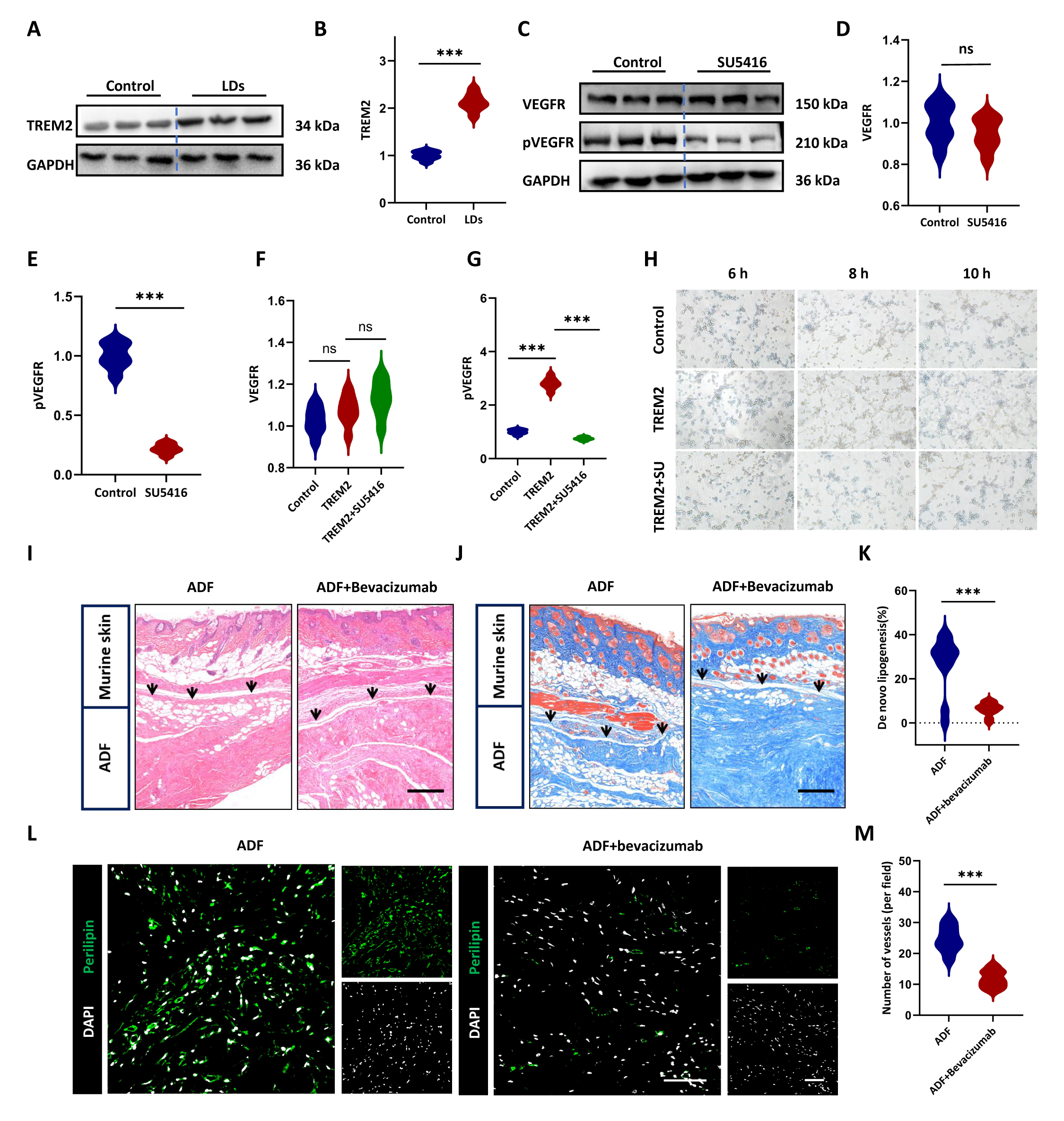


**Supplementary FIGURE 9.** Function of trem2^+^ macrophages in modulation of M1/M2 macrophage polarization. (A) Trem2^+^ macrophages exhibited the interactions with both CD86^+^ macrophages (M1) and CD163^+^ macrophages (M2). (B) Experiment scheme. qPCR analysis of M1/M2 macrophage marker (C) iNOS, (D) Arg1. qPCR analysis (E) TNF-α and (F) IL-10. (n = 8 per group). *p < 0.05; **p < 0.01; ***p < 0.001; “ns” means non-significant difference.


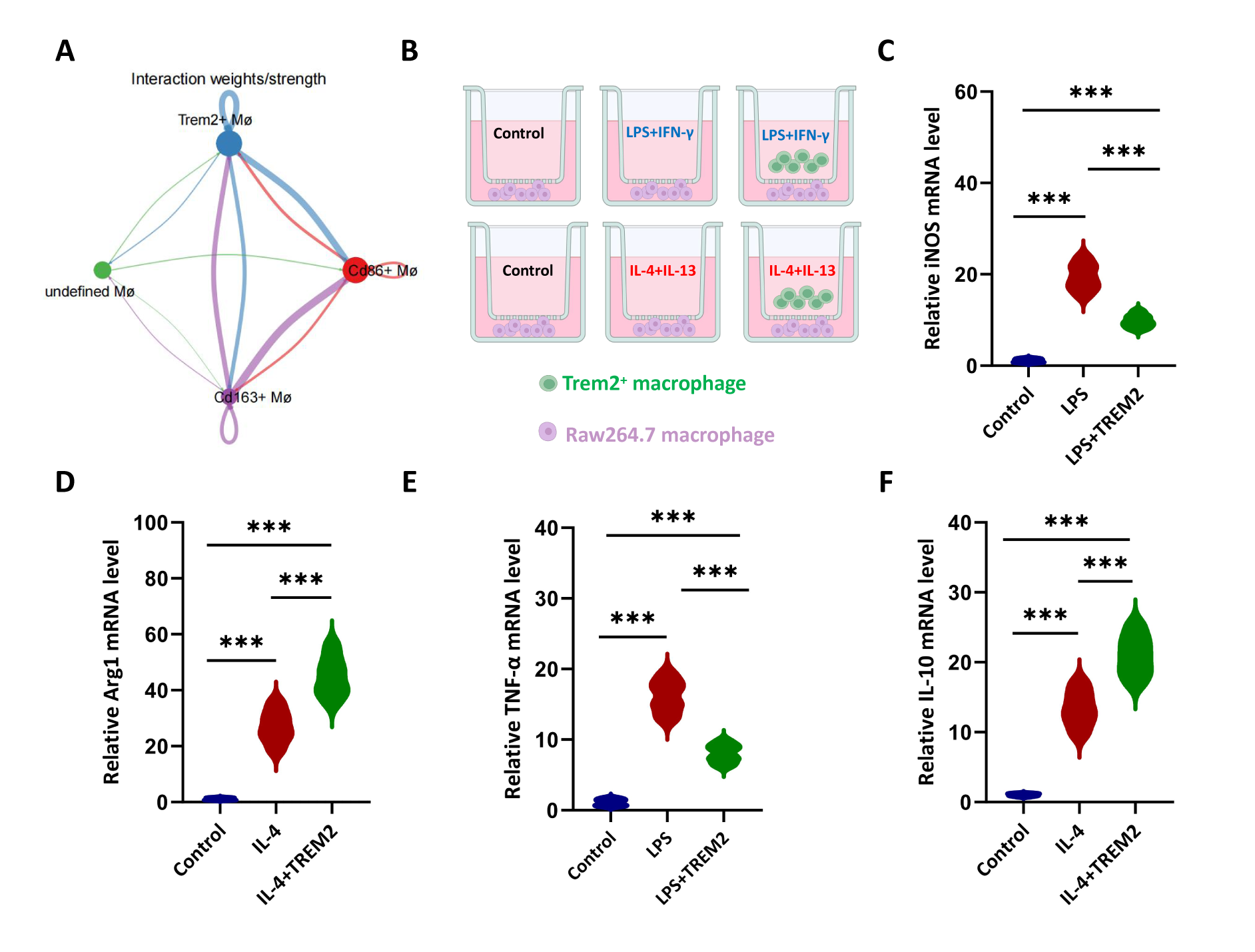


**Supplementary FIGURE 10.** Potential application of ADF in various regenerative repairs. (A) Potential application of ADF. (B) Sutured onto the surface of the prosthesis for application. (C) The “sandwich” composite membrane construction. (D) ECM bank for storage of ADF. (E) HE staining of ADF preparation with adipose tissue from C57/BL mice. Scale bars, 500 μm in(left) and 100 μm in (right).


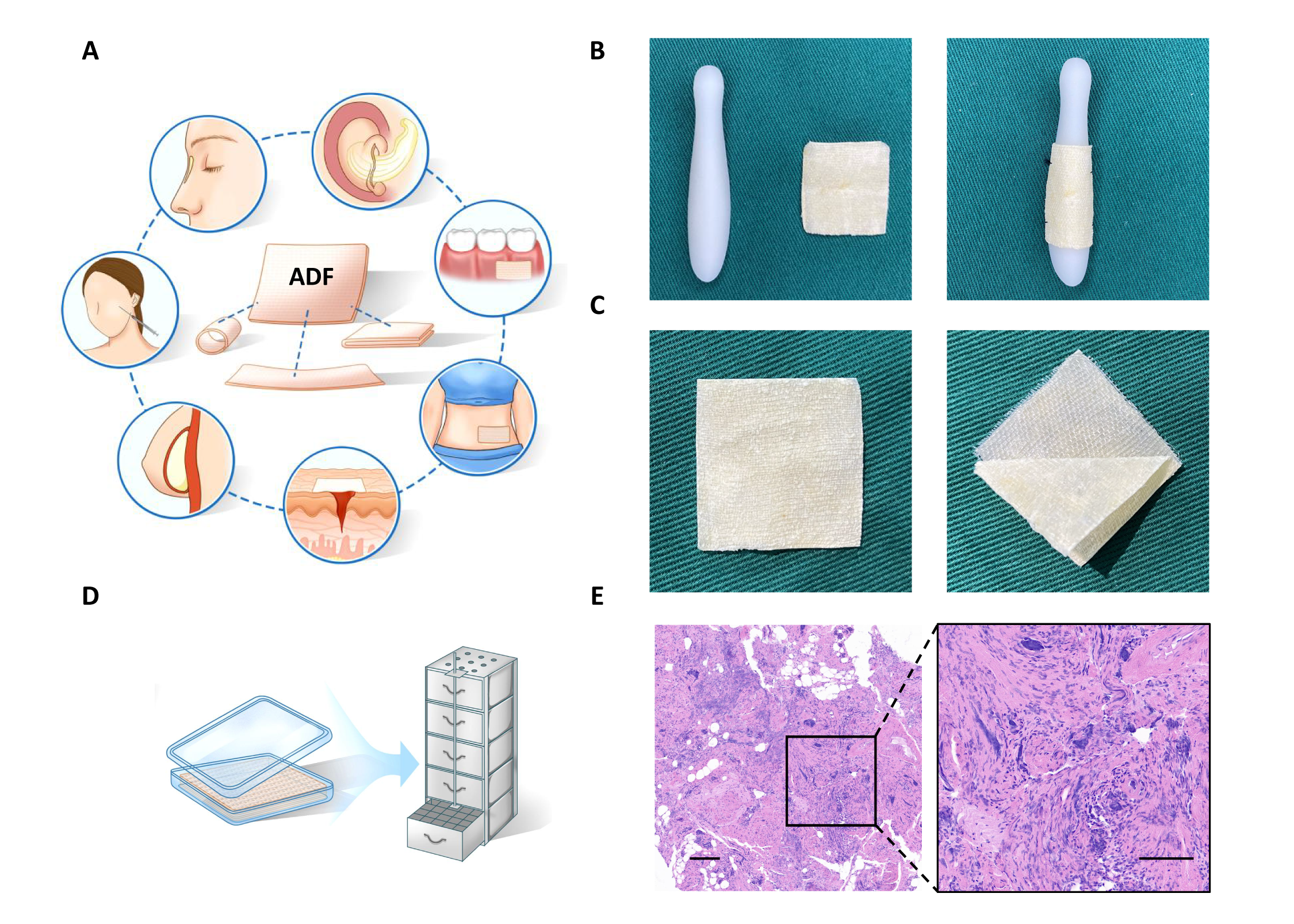


**Table 1: Blood Routine Examination of 4 Weeks**

|  | **Control** | **ADF** | **F-ADF** |
| --- | --- | --- | --- |
| WBC (10^9^ /L) | 1.79 ± 0.27 | 1.72 ± 0.21 | 1.81 ± 0.12 |
| Neu (10^9^ /L) | 0.94 ± 0.19 | 0.91 ± 0.09 | 0.96 ± 0.21 |
| Lym (10^9^ /L) | 0.77 ± 0.15 | 0.68 ± 0.13 | 0.72 ± 0.51 |
| Mon (10^9^ /L) | 0.05 ± 0.02 | 0.05 ± 0.06 | 0.04 ± 0.03 |
| Eos (10^9^ /L) | 0.04 ± 0.02 | 0.04 ± 0.01 | 0.03 ± 0.09 |
| Bas (10^9^ /L) | 0 ± 0 | 0 ± 0 | 0 ± 0 |
| Neu (%) | 52.13 ± 4.96 | 51.32 ± 4.89 | 52.4 5 ± 4.13 |
| Lym (%) | 42.43 ± 4.6 | 42.47 ± 4.7 | 42.75 ± 4.1 |
| Mon (%) | 3.09 ± 1.4 | 3.05 ± 1.2 | 3.12 ± 1.1 |
| Eos (%) | 2.21 ± 1.04 | 2.25 ± 1.03 | 2.19 ± 1.45 |
| Bas (%) | 0.14 ± 0.11 | 0.14 ± 0.09 | 0.16 ± 0.05 |
| RBC (10^12^/L) | 8.23 ± 0.73 | 8.14 ± 0.53 | 8.27 ± 0.89 |
| HGB (g/L) | 132.4 ± 11.76 | 133.7 ± 11.35 | 132.9 ± 11.24 |
| HCT (%) | 41.32 ± 3.5 | 41.57 ± 3.1 | 41.07 ± 3.9 |
| MCV (fL) | 50.27 ± 0.98 | 50.34 ± 0.78 | 50.51 ± 0.14 |
| MCH (pg) | 16.08 ± 0.43 | 16.13 ± 0.03 | 16.75 ± 0.41 |
| MCHC (g/L) | 320.4 ± 4.25 | 321.4 ± 4.22 | 320.1 ± 4.97 |
| RDW-CV (%) | 13.45 ± 0.54 | 13.51 ± 0.49 | 13.37 ± 0.76 |
| RDW-SD (fL) | 28.01 ± 0.93 | 27.03 ± 0.87 | 28.12 ± 0.33 |
| PLT (10^9^ /L) | 608.5 ± 82.91 | 607.5 ± 82.35 | 610.5 ± 82.11 |
| MPV (fL) | 5.65 ± 0.24 | 5.61 ± 0.56 | 5.57 ± 0.13 |
| PDW | 15.34 ± 0.21 | 15.37 ± 0.46 | 15.17 ± 0.88 |
| PCT (%) | 0.34 ± 0.05 | 0.31 ± 0.08 | 0.34 ± 0.14 |
| WBC: White Blood Cell Count; Neu:Neutrophil Count; Lym:Lymphocyte Count; Mon:Monocyte Count; Eos:Eosinophil Count; Bas:Basophil Count; Neu:Neutrophil Percentage; Lym:Lymphocyte Percentage; Mon:Monocyte Percentage; Eos:Eosinophil Percentage; Bas:Basophil Percentage; RBC: Red Blood Cell Count；HGB: Hemoglobin；HCT: Hematocrit；MCV: Mean Corpuscular Volume；MCH: Mean Corpuscular Hemoglobin；MCHC: Mean Corpuscular Hemoglobin Concentration；RDW-CV: Red Cell Distribution Width (Coefficient of Variation)；RDW-SD: Red Cell Distribution Width (Standard Deviation)；PLT: Platelet Count；MPV: Mean Platelet Volume；PDW: Platelet Distribution Width；PCT: Plateletcrit | | | |

**Table 2: Blood Routine Examination of 12 Weeks**

|  | **Control** | **ADF** | **F-ADF** |
| --- | --- | --- | --- |
| WBC (10^9^ /L) | 1.82 ± 0.26 | 1.71 ± 0.22 | 1.80 ± 0.13 |
| Neu (10^9^ /L) | 0.93 ± 0.18 | 0.92 ± 0.08 | 0.95 ± 0.20 |
| Lym (10^9^ /L) | 0.76 ± 0.16 | 0.69 ± 0.14 | 0.71 ± 0.50 |
| Mon (10^9^ /L) | 0.06 ± 0.03 | 0.04 ± 0.05 | 0.05 ± 0.04 |
| Eos (10^9^ /L) | 0.03 ± 0.03 | 0.05 ± 0.02 | 0.04 ± 0.08 |
| Bas (10^9^ /L) | 0 ± 0 | 0 ± 0 | 0 ± 0 |
| Neu (%) | 52.20 ± 4.95 | 51.30 ± 4.90 | 52.50 ± 4.12 |
| Lym (%) | 42.40 ± 4.59 | 42.50 ± 4.69 | 42.80 ± 4.09 |
| Mon (%) | 3.10 ± 1.39 | 3.04 ± 1.21 | 3.11 ± 1.11 |
| Eos (%) | 2.20 ± 1.05 | 2.26 ± 1.02 | 2.18 ± 1.44 |
| Bas (%) | 0.15 ± 0.10 | 0.13 ± 0.08 | 0.15 ± 0.06 |
| RBC (10^12^/L) | 8.22 ± 0.74 | 8.15 ± 0.52 | 8.26 ± 0.88 |
| HGB (g/L) | 132.3 ± 11.75 | 133.8 ± 11.34 | 133.0 ± 11.23 |
| HCT (%) | 41.30 ± 3.51 | 41.60 ± 3.09 | 41.10 ± 3.89 |
| MCV (fL) | 50.26 ± 0.97 | 50.35 ± 0.77 | 50.50 ± 0.15 |
| MCH (pg) | 16.09 ± 0.42 | 16.12 ± 0.04 | 16.74 ± 0.40 |
| MCHC (g/L) | 320.3 ± 4.26 | 321.5 ± 4.21 | 320.2 ± 4.96 |
| RDW-CV (%) | 13.44 ± 0.55 | 13.50 ± 0.48 | 13.38 ± 0.75 |
| RDW-SD (fL) | 28.00 ± 0.94 | 27.02 ± 0.86 | 28.11 ± 0.34 |
| PLT (10^9^ /L) | 608.6 ± 82.90 | 607.4 ± 82.34 | 610.4 ± 82.10 |
| MPV (fL) | 5.64 ± 0.25 | 5.62 ± 0.55 | 5.58 ± 0.14 |
| PDW | 15.33 ± 0.22 | 15.38 ± 0.45 | 15.18 ± 0.87 |
| PCT (%) | 0.35 ± 0.06 | 0.32 ± 0.07 | 0.33 ± 0.13 |
| WBC: White Blood Cell Count; Neu:Neutrophil Count; Lym:Lymphocyte Count; Mon:Monocyte Count; Eos:Eosinophil Count; Bas:Basophil Count; Neu:Neutrophil Percentage; Lym:Lymphocyte Percentage; Mon:Monocyte Percentage; Eos:Eosinophil Percentage; Bas:Basophil Percentage; RBC: Red Blood Cell Count；HGB: Hemoglobin；HCT: Hematocrit；MCV: Mean Corpuscular Volume；MCH: Mean Corpuscular Hemoglobin；MCHC: Mean Corpuscular Hemoglobin Concentration；RDW-CV: Red Cell Distribution Width (Coefficient of Variation)；RDW-SD: Red Cell Distribution Width (Standard Deviation)；PLT: Platelet Count；MPV: Mean Platelet Volume；PDW: Platelet Distribution Width；PCT: Plateletcrit | | | |

**Table 3: Blood Biochemical Analysis of 4 Weeks**

|  | **Control** | **ADF** | **F-ADF** |
| --- | --- | --- | --- |
| ALT (U/L) | 31.22 ± 9.43 | 30.98 ± 9.12 | 31.43 ± 8.98 |
| AST(U/L) | 240.86 ± 132.25 | 241.57 ± 130.14 | 239.98 ± 131.45 |
| TG(mmol/L) | 1.37 ± 0.41 | 1.34 ± 0.42 | 1.35 ± 0.34 |
| TC(mmol/L) | 2.58 ± 0.42 | 2.56 ± 0.34 | 2.61 ± 0.25 |
| Glu-G(mmol/L) | 4.66 ± 1.29 | 4.36 ± 1.31 | 4.27 ± 1.16 |
| ALP (U/L) | 193.81 ± 25.21 | 190.65 ± 25.21 | 189.81 ± 24.76 |
| γ-GT (U/L) | 1.46 ± 0.48 | 1.37 ± 0.52 | 1.49 ± 0.21 |
| CREA(umol/L) | 10.86 ± 1.56 | 10.23 ± 0.52 | 11.02 ± 0.11 |
| TP (g/L) | 55.38 ± 2.53 | 54.25 ± 2.33 | 55.22 ± 2.73 |
| α-AMY (U/L) | 3136.44 ± 925.96 | 3142.44 ± 923.94 | 3125.41 ± 947.36 |
| CK (U/L) | 2364.61 ± 1613.78 | 2378.16 ± 1613.32 | 2367.54 ± 1611.24 |
| LDH (U/L) | 1027.5 ± 172.53 | 1021.5 ± 174.36 | 1030.5 ± 171.61 |
| ALB Ⅱ (g/L) | 31.12 ± 1.34 | 30.21 ± 1.45 | 31.56 ± 1.31 |
| LIP (U/L) | 32.41 ± 5.73 | 33.15 ± 5.71 | 32.56 ± 5.32 |
| UREA(mmol/L) | 8.65 ± 1.1 | 8.63 ± 1.0 | 8.72 ± 1.4 |
| Ca(mmol/L) | 2.25 ± 0.07 | 2.31 ± 0.04 | 2.28 ± 0.13 |
| P(mmol/L) | 2.44 ± 0.2 | 2.56 ± 0.3 | 2.47 ± 0.1 |
| Mg Ⅱ(mmol/L) | 1.47 ± 0.08 | 1.46 ± 0.03 | 1.51 ± 0.11 |
| BUN(mmol/L) | 5.07 ± 0.37 | 5.17 ± 0.32 | 5.64 ± 0.72 |
| ALT: Alanine Aminotransferase；AST: Aspartate Aminotransferase ；ALP: Alkaline Phosphatase ；γ-GT: Gamma-Glutamyl Transferase ；ALB: Albumin ；TP: Total ProteinCREA: Creatinine ；UREA: Urea ；BUN: Blood Urea Nitrogen；TG: Triglycerides ；TC: Total Cholesterol ；Glu-G: GlucoseCK: Creatine Kinase ；LDH: Lactate Dehydrogenase ；α-AMY: Alpha-Amylase ；LIP: LipaseCa: Calcium ；P: Phosphorus ；Mg: Magnesium | | | |

**Table 4: Blood Biochemical Analysis of 12 Weeks**

|  | **Control** | **ADF** | **F-ADF** |
| --- | --- | --- | --- |
| ALT (U/L) | 31.20 ± 9.42 | 30.99 ± 9.11 | 31.43 ± 9.17 |
| AST(U/L) | 240.90 ± 132.20 | 241.55 ± 130.16 | 239.99 ± 131.41 |
| TG(mmol/L) | 1.38 ± 0.40 | 1.35 ± 0.41 | 1.36 ± 0.33 |
| TC(mmol/L) | 2.59 ± 0.41 | 2.57 ± 0.33 | 2.60 ± 0.26 |
| Glu-G(mmol/L) | 4.67 ± 1.28 | 4.35 ± 1.30 | 4.28 ± 1.15 |
| ALP (U/L) | 193.80 ± 25.20 | 190.64 ± 25.20 | 189.82 ± 24.75 |
| γ-GT (U/L) | 1.47 ± 0.47 | 1.38 ± 0.51 | 1.50 ± 0.20 |
| CREA(umol/L) | 10.85 ± 1.55 | 10.24 ± 0.51 | 11.03 ± 0.12 |
| TP (g/L) | 55.37 ± 2.52 | 54.26 ± 2.32 | 55.23 ± 2.72 |
| α-AMY (U/L) | 3136.40 ± 925.95 | 3142.45 ± 923.93 | 3125.40 ± 947.35 |
| CK (U/L) | 2364.60 ± 1613.77 | 2378.15 ± 1613.31 | 2367.55 ± 1611.23 |
| LDH (U/L) | 1027.4 ± 172.52 | 1021.4 ± 174.35 | 1030.6 ± 171.60 |
| ALB Ⅱ (g/L) | 31.11 ± 1.33 | 30.20 ± 1.44 | 31.55 ± 1.30 |
| LIP (U/L) | 32.40 ± 5.72 | 33.14 ± 5.70 | 32.55 ± 5.31 |
| UREA(mmol/L) | 8.66 ± 1.09 | 8.64 ± 1.01 | 8.73 ± 1.39 |
| Ca(mmol/L) | 2.26 ± 0.08 | 2.32 ± 0.05 | 2.27 ± 0.14 |
| P(mmol/L) | 2.45 ± 0.19 | 2.55 ± 0.29 | 2.48 ± 0.11 |
| Mg Ⅱ(mmol/L) | 1.48 ± 0.09 | 1.45 ± 0.04 | 1.52 ± 0.10 |
| BUN(mmol/L) | 5.08 ± 0.36 | 5.18 ± 0.31 | 5.63 ± 0.71 |
| ALT: Alanine Aminotransferase；AST: Aspartate Aminotransferase ；ALP: Alkaline Phosphatase ；γ-GT: Gamma-Glutamyl Transferase ；ALB: Albumin ；TP: Total ProteinCREA: Creatinine ；UREA: Urea ；BUN: Blood Urea Nitrogen；TG: Triglycerides ；TC: Total Cholesterol ；Glu-G: GlucoseCK: Creatine Kinase ；LDH: Lactate Dehydrogenase ；α-AMY: Alpha-Amylase ；LIP: LipaseCa: Calcium ；P: Phosphorus ；Mg: Magnesium | | | |

**5. Materials and methods**

- 1. **Materials**

Histological assessments were performed using Hematoxylin and eosin (HE) staining kit (Sigma-Aldrich, USA, Cat. No. HT110116), Masson's trichrome staining kit (Sigma-Aldrich, USA, Cat. No. HT15), and Oil Red O staining solution (Sigma-Aldrich, USA, Cat. No. O0625). Physical characterization was conducted using FT-IR spectroscopy (Thermo Fisher Scientific, USA), tensile testing machine (Instron, USA), scanning electron microscope (Hitachi, Japan), and laser confocal microscope (Zeiss, Germany). Molecular analysis employed Quant-iT™ PicoGreen® dsDNA Assay Kit (Thermo Fisher, USA, Cat. No. P11496), Proteinase K (Sigma-Aldrich, USA, Cat. No. P2308), DNase I (Roche, Switzerland, Cat. No. 4716728001), ELISA kits for IL-6 (R&D Systems, USA, Cat. No. DY506), TNF-α (R&D Systems, USA, Cat. No.DY410), CRP (R&D Systems, USA, Cat. No.DY1707), and VEGFA (R&D Systems, USA, Cat. No. DVE00), TRIzol® Reagent (Invitrogen, USA, Cat. No. 15596026), SYBR Green Master Mix (Applied Biosystems, USA, Cat. No. 4309155), Collagenase Type I (Worthington, USA, Cat. No. LS004196), and ACK Lysing Buffer (Gibco, USA, Cat. No. A1049201). In vivo studies used nude mice (6-8 weeks old, Fourth Military Medical University Animal Center, China) with Isoflurane anesthesia (Baxter, USA, Cat. No. 1001936060), 7-0 nylon sutures (Ethicon, USA), physiological saline (Sigma-Aldrich, USA, Cat. No. S8776), Clodronate Liposomes (Liposoma BV, Netherlands, Cat. No. CP-005-005), SU5416 (VEGFR2 inhibitor, Selleckchem, USA, Cat. No. S1494), Bevacizumab (Avastin, Genentech, USA), Cisplatin (Sigma-Aldrich, USA, Cat. No. P4394) and RGD competing peptides (Sigma-Aldrich, USA, Cat. No. SCP0190). Cell culture and functional assays using EdU Cell Proliferation Kit (Beyotime, China, Cat. No. C0071S), Transwell chambers (Corning, USA, Cat. No. 3422), Matrigel (Corning, USA, Cat. No. 356234), CCK-8 Assay Kit (Dojindo, Japan, Cat. No. CK04), Live/Dead Cell Assay Kit (Thermo Fisher, USA, Cat. No. L3224), Hoechst 33342 (Thermo Fisher, USA, Cat. No. H3570), and RGD-competing peptides (Sigma-Aldrich, USA, Cat. No. SCP0190). Protein analysis was performed using RIPA Buffer (Pierce, USA, Cat. No. 89900), PVDF Membranes (Millipore, USA, Cat. No. IPVH00010), SDS-PAGE gels (Bio-Rad, USA, Cat. No. 4561096), and LTQ mass spectrometer (Thermo Fisher, USA). a-Galactosidase Microplate Assay Kit (Abbioscience (Shanghai) Biological Technology Co., Ltd.). Immunostaining employed DAPI (Sigma-Aldrich, USA, Cat. No. D9542), EDTA-K2 Tubes (BD Biosciences, USA, Cat. No. 366643), and antibodies against CD3 (Servicebio, China, Cat. No. GB13421), CD19 (Servicebio, China, Cat. No. GB11064), perilipin 1 (Abcam, UK, Cat. No. ab3526), CD31 (Servicebio, China, Cat. No. GB11063-2), α-SMA (Servicebio, China, Cat. No. GB12045), F4/80 (Servicebio, China, Cat. No. GB113373), CD11c (Servicebio, China, Cat. No. GB11059), Ly6G (Servicebio, China, Cat. No. GB11229), CD14 (Servicebio, China, Cat. No. GB14023-50), CD206 (Servicebio, China, Cat. No. GB113497-100), iNOS (Servicebio, China, Cat. No. GBGB11119), TREM2 (Abcam, UK, Cat. No. ab305103), COL1 (Abcam, UK, Cat. No. ab279711) ,VEGFR2 (Cell Signaling Technology, USA, Cat. No. 2479), phospho-VEGFR2 (Tyr1175) (Cell Signaling Technology, USA, Cat. No. 2478),GAPDH (Proteintech, USA, Cat. No. 60004-1-Ig), GFP (Abcam, UK, Cat. No. ab13970), VEGFA (Abcam, UK, Cat. No. ab52917), FSP1 (Abcam, UK, Cat. No. ab27957), and FN1 (Abcam, UK, Cat. No. ab2413).

**5.2 Preparation of the ADF**

Human adipose tissue collection were approved by the Ethics Committee of the Fourth Military Medical University (No. KY20243548-1). Liposuction and abdominoplasty patients undergo surgical procedures as previously described. Residual edema fluid and blood components are removed by washing. The method to obtain adipose tissue from liposuction is through physical sorting using syringes and forceps, to isolate the adipose tissue derived matrix. Mechanical methods are used to break adipose tissue, mainly through mechanical crushing methods (1500~2000 rpm for 1 minute, such as homogenization, grinding, etc.) to further process the adipose tissue derived matrix, with the aim of breaking adipose tissue until no yellow fat is visible, at which point the adipose tissue derived matrix appear white. Gently pull out the white fibers with forceps. Most of the crushed cellular components are removed through washing, with repeated washing to remove cell debris and oil droplets, using deionized water or physiological saline for 1-3 times, each for 0.5 minutes. After washing, the adipose tissue derived matrix are isolated with forceps. Through mechanical pressing in a fixed mold, the material is formed into membrane patches of consistent thickness.. The obtained adipose tissue derived matrix are wrapped into a specific size using a polyethylene mesh approved by the FDA. The wrapped adipose tissue derived matrix are placed in a specific mold, with filter paper or other absorbent materials on the top and bottom to absorb the squeezed-out oil and water. External pressure is applied for pressing, with a pressure of 1 x 10^5^-1 x 10^6^ kPa (3 minutes). After pressing, remove the wrapped mesh to obtain a clinically ready human adipose tissue-derived matrix membrane patch, adipose-derived matrix film (ADF). (The membrane patch can also be formed under insufficient pressure, but the removal of water and oil is not as complete as above protocol).

**5.3 Morphological Studies of Different Stages in the ADF Preparation**

To further evaluate the impact of each treatment method on adipose tissue, hematoxylin and eosin(HE) staining, Masson's trichrome staining, and Oil red O staining were conducted.

*HE staining*: Take samples of different stages in ADF preparation and place them in EP tubes with 4 % paraformaldehyde tissue fixative. Dehydrate the samples using a gradient alcohol concentration method, typically choosing concentrations of 50 %-60 %-70 %-90 %-95 %-100 %, with each alcohol concentration being precise. Dehydrate for 1 hour at each step before 95 %, and for 95 % and 100 %, the process is divided into two steps, each for 30 minutes. To facilitate the entry of paraffin into the tissue, the dehydrated tissue must be cleared with a solution of xylene : absolute ethanol (1: 1) and then with xylene for 30 minutes each. Soak the samples thoroughly in a paraffin solution at 60 °C for 2 h. Embed the soaked tissue blocks using a paraffin embedding machine, then place them on a cold plate to solidify. After that, trim the blocks and fix them on a microtome for continuous sectioning (5 µm). The sections are put in a 42 °C warm water bath with forceps, then fixed onto glass slides, dried at 40 °C, and then subjected to HE staining. Immediately after removing the sections from the incubator, immerse them in xylene for 5-10 minutes to dewax. Transfer them into absolute ethanol (100 %) for about 2 minutes. Pass through 90 %, 80 %, and 70 % alcohol for about 2 minutes each. Finally, transfer into water to wash off the alcohol for about 2-3 minutes, then into distilled water for about 2 minutes. Immerse the sections in hematoxylin for 8-15 minutes, with a slightly deep color being preferable. Transfer into water to wash off the hematoxylin and floating color for about 1-2 minutes. Transfer into the differentiation solution (1 % hydrochloric acid alcohol) for a few seconds to 30 seconds to lighten the sections to a pale blue. Rinse in running water for 30-60 minutes until the tissue appears bright blue or sky blue (blueing). Transfer into eosin solution for 2-5 minutes; if staining is slow, add glacial acetic acid to the eosin solution to assist. Transfer into water to wash off the eosin floating liquid and wipe the excess dye off the slide with a gauze. Dehydrate through absolute alcohol, then make the sections transparent with xylene. Apply neutral resin to the transparent sections and cover with a cover slip to seal.

*Masson's trichrome staining*: The processes of sample fixation, embedding, sectioning, and dewaxing are as described above. After completion, place the tissue sections in a potassium dichromate at room temperature for 12-18 h, then briefly rinse with running water and use Weigert's iron hematoxylin staining solution for 5 minutes. Rinse with water for 3-5 minutes twice until colorless, and then dry. Place in 1 % hydrochloric alcohol for 1-2 seconds. Terminate the differentiation in water for 10 minutes. Use acid fuchsin for 10 minutes, and the tissue will appear bright red under the microscope. Rinse with phosphomolybdic acid solution, and the rinsing time will depend on the type and condition of the tissue sections. Terminate the differentiation when the collagen around the blood vessels fades from bright red to light red to colorless under the microscope. Use aniline blue staining solution for 30 seconds. Rinse with water for 2 seconds. Dehydrate the sections through the following steps: absolute ethanol for 30 seconds,absolute ethanol for 1 minute, xylene for 1 minute, xylene for 2 minutes. Remove the sections from xylene and let them air dry slightly before sealing with neutral resin.

*Oil Red O Staining*: The processes of sample fixation, embedding, sectioning, and dewaxing are as described above. After completion, briefly rinse the sections with 60 % isopropanol to remove residual water. Immerse the sections in Oil Red O staining solution for 15-20 minutes. After staining, briefly rinse the sections with 60 % isopropanol to remove excess staining solution. Counterstain with hematoxylin or another nuclear dye to stain the cell nuclei. Dehydrate through a gradient of ethanol, then make transparent with xylene. Seal with neutral resin or another mounting medium.

**5.4 Basic Performance Evaluation of the ADF**

*FT-IR Test of the ADF:* Transfer the sample to be tested into a three-well plate and mount the plate on a sample holder. Install the sample holder on the sample platform, ensuring the sample is securely fixed. Open the software interface, select the "View and Collect" screen, and click on the transmission collection mode and the detector in use. Adjust both the reflection and transmission illumination to the appropriate brightness, move the virtual joystick to locate and focus on the sample. Click the "Infrared Energy" icon to optimize the condenser height. Set the collection parameters in the software, including the size of the aperture. Click the collection button to start collecting the sample spectrum.

*Tensile Test:* Cut the ADF and F-ADF into shapes and sizes as stipulated by standard methods. Employ an universal testing machine (UTM) capable of performing various tests such as tension, compression, bending, and shearing. Secure both ends of the ADF and F-ADF in the machine's clamps to ensure it can withstand axial tensile force. Apply axial tensile force to the specimen until it breaks. Record the applied tensile force and the corresponding elongation to obtain the stress-strain curve. To better evaluate the performance of the membrane in an in vivo environment, the membrane was pre-warmed in a 37 °C incubator and its properties were promptly tested.

*Scanning Electron Microscopy (SEM) Test:* Place the ADF, F-ADF and ADM samples to be observed on a sample holder and insert the holder into the SEM's sample stage. Ensure the holder is securely connected to the stage to prevent movement or tilting during scanning. Turn on the vacuum pump and start it working. Wait until the vacuum pump has evacuated the interior of the electron microscope to a high vacuum state. Monitor the vacuum level to ensure it meets the required working conditions. In the SEM Control Panel, set parameters such as EHT target (accelerating voltage), I Probe (probe current), and Filament I target (filament current). Turn on the beam, activating the filament and accelerating voltage. Set the scanning speed and Noise reduction mode, and save the image when the scanning is complete. Use software to analyze the collected images to obtain surface morphology and structural information of the ADF and F-ADF samples.

*Laser Confocal Microscopy Detection of the ADF:* Under the locate module, select the bright-field RBF mode, adjust the optical path settings, and focus under the objective lens to find the area to be photographed. Switch to LSM for confocal settings and acquisition. Set the excitation wavelength for the fluorescent sample according to the experimental requirements, and select the appropriate filter cube to ensure the photomultiplier tube (PMT) detector receives sufficient signal. Adjust the magnification of the objective lens in visual mode, and locate the cells or area to be inspected under the fluorescence microscope. Switch to scanning mode, adjust the pinhole and laser intensity parameters to obtain a clear confocal image. Choose the appropriate image resolution, scan the sample completely, and save the image results.

*Swelling Test of the ADF:* Weigh a certain amount of ADF and F-ADF set aside. Add a certain amount of distilled water to a graduated cylinder and place it in a constant temperature water bath to heat to 37 °C. Use an electronic balance to weigh the dry ADF and F-ADF and record the mass (md). Add the dry ADF to the solvent and allow it to swell fully. After certain time intervals (24 h, 48 h, 72 hand 144 h), remove the swollen ADF, dry the surface moisture with filter paper, and then weigh and record the mass (mt ). Calculate the swelling ratio: Use the formula SR = (mt-md)/md × 100 % to calculate the swelling ratio (SR), where md is the initial dry mass of ADF, and mt is the mass after swelling. We measured the changes in volume at the same time points and conducted statistical analysis.

*ECM Degradation Analysis*: Samples of ADF and F-ADF (20-50 mg) are homogenized in degradation buffer (50 mM Tris-HCl, 150 mM NaCl, 5 mM CaCl₂, pH 7.4) with protease inhibitors and subjected to enzymatic digestion using collagenase Type I (100 U/mL, 4 h) at 37 °C. Reactions are terminated with 10 mM EDTA, followed by centrifugation (12,000×g, 10 min) to collect supernatants for quantitative analysis. All data are normalized to tissue weight with controls including no-enzyme blanks and heat-inactivated enzymes.

*Residual DNA Detection:* To assess residual DNA in ADF and F-ADF, samples (10 mg) are homogenized in TE buffer, digested with Proteinase K (56 °C, 2 h), and quantified using the Quant-iT PicoGreen assay (detection limit: 0.1 ng/µL) with fluorescence measurement (Ex/Em 480/520 nm). A standard curve (0–100 ng/mL genomic DNA) validates quantificatio. Controls include human genomic DNA (positive) and nuclease-free water (negative). Residual DNA in D-ADF has also been assessed.

*TUNEL Apoptosis Detection:* Fixed tissue sections (4-5 μm) are permeabilized (0.1 % Triton X-100, 10 min) and incubated with TUNEL reaction mix (TdT enzyme + fluorescein-dUTP, 37 °C, 1 h) to label DNA breaks, with DNase I-treated samples as positive controls. After PBS washes, nuclei are counterstained with DAPI (1 μg/mL, 5 min) and mounted for fluorescence microscopy (FITC: 488/520 nm; DAPI: 358/461 nm). Apoptotic cells are quantified as (TUNEL⁺ cells / total DAPI⁺ cells) × 100 % using ImageJ.

**5.5 Subcutaneous Implantation of ADF in Nude Mice Model**

All animal experiments are approved by the Ethics Committee of the Fourth Military Medical University (IACUC No. 20240726). The trials used nude male mice (6- to 8-week-old) acquire from the animal center of the Fourth Military Medical University, China. Preparation of the ADF (about 1.5 cm × 1.5 cm) for implantation is as mentioned above. Anesthesia is administered to the nude mice using a inhalational anesthesia system, followed by sterilization of the dorsal skin. A 2 cm incision is then made on the back of the nude mouse, and a cavity approximately 2 cm × 2 cm is carefully dissected using scissors. The ADF and F-ADF are moistened with physiological saline before transplantation. They are carefully grasped with forceps and placed into the dissected cavity, ensuring they are spread out without any folded areas. The incision is then meticulously sutured with 7-0 nylon sutures, and the area is sterilized again. Nude mice were kept warm at 37 °C. The mice are housed separately to prevent them from biting each other. The sutures are removed after 7 days (they often fall off on their own). The Implanted tissues are retrieved for evaluation at weeks 2, 4, 12, and 24 post-transplantation. At the end of the experiment, the mice are euthanized by carbon dioxide asphyxiation.

**5.6 In Vitro Biocompatibility and In Vivo Safety Assessment of ADF**

*Live/Dead Cell Assay of the ADF:* By co-culturing with keratinocytes (HaCat), fibroblasts (HDFs), vascular endothelial cells (HUVECs) and adipose-derived stem cells (ADSCs), cell compatibility of ADF and F-ADF was assessed. Remove the culture medium from the co-incubated cells and gently wash the cells with phosphate-buffered saline (PBS) 1-2 times to remove residual medium and serum, which could interfere with subsequent staining processes. Prepare the working solutions of live cell dye (Calcein AM) and dead cell dye (Propidium Iodide, PI) at appropriate concentrations according to the instructions provided with the live/dead cell staining kit. Mix the washed cells with the prepared staining working solutions, ensuring that the dyes evenly cover the cells. Incubate at room temperature in the dark for a certain period (usually 10-30 minutes) to allow the dyes to fully penetrate the cells and bind to the target substances. After incubation, wash the cells again with PBS to remove unbound dyes and background interference. Observe the cells using a fluorescence microscope or flow cytometer. Live cells will emit green fluorescence, while dead cells will emit red fluorescence. Collect data and perform further analysis to calculate the proportion of live cells and apoptotic cells.

*Hemolysis Test:* Take 10-20 ml of fresh blood and place it in a triangular flask containing glass beads, shake for 10 minutes, or stir the blood with a glass rod to remove fibrinogen and make it fibrin-free. Add 100 ml of physiological saline, mix well, and centrifuge at 1000-1500 rpm for 15 minutes to remove the supernatant. The precipitated red blood cells are then washed 2-3 times with physiological saline as described above, until the supernatant is no longer red. Prepare a 2 % suspension of the obtained red blood cells with physiological saline for testing (2 ml of red blood cells added to 100 ml of saline). Weigh a certain amount of ADF and F-ADF and place it in a 1.5 mL centrifuge tube, add DPBS solution and stabilize for 15 min. In a 200 μL blood sample, add 250 μL of PBS to dilute the anticoagulant-added blood, ready for use. Prepare two clean 1.5 mL centrifuge tubes, add 3 mL of water and 3 mL of DPBS solution respectively, then add 100 μL of diluted blood to each of the three 15 mL centrifuge tubes, and incubate at 37 °C for 1 hour. After centrifugation at 5000 rpm at room temperature for 3 minutes, hemolysis is considered significant if the hemolysis rate exceeds 5 %. Triton X-100 serves as the positive control and PBS is as the negative control.

*Serum IL-6, TNF-α, and CRP detection using ELISA:* To evaluate systemic inflammation, 500 μL blood samples are collected from mice via retro-orbital bleeding using heparinized capillaries or 1 mL syringes. The whole blood is allowed to clot at room temperature for 30 min, followed by centrifugation at 3000 g for 15 min at 4 °C to separate serum, which is then aliquoted (50 μL/tube) and stored at -80 °C until analysis. For inflammatory marker quantification, serum concentrations of IL-6, TNF-α, and CRP are determined using standardized ELISA kits ( R&D Systems). Briefly, 96-well plates are coated with capture antibodies (4 °C overnight), blocked with 1 % BSA in PBS (37 °C, 1 h), and incubated with serially diluted standards (0-500 pg/mL) and test samples (100 μL/well). After sequential incubations with detection antibodies (37 °C, 2 h) and streptavidin-HRP (37 °C, 30 min), the reaction is developed with TMB substrate (15 min) and stopped with 1 M H₂SO₄. Absorbance (450 nm) is measured using a microplate reader, with analyte concentrations calculated from standard curves via 4-parameter logistic regression.

*Nude mice Complete Blood Count (CBC) Analysis:* Blood is collected via retro-orbital or submandibular puncture using EDTA-coated capillaries (500 μL per mouse) and immediately mixed with 10 % EDTA-K2 in PBS (1: 9 ratio) to prevent clotting. Samples are analyzed within 1 hour on an animal optimized hematology analyzer with species specific settings.

*Nude mice Blood Biochemical Analysis:* Blood is collected via retro-orbital puncture or cardiac puncture (terminal) into serum separator or EDTA tubes, with serum obtained by centrifugation (3000×g, 10min, 4 °C) after 30min clotting. Key parameters are analyzed using an automated biochemistry analyzer.

*CD68 positive cells of vital organ:* Fixed tissues of vital organ are embedded in paraffin (4 % PFA-fixed), sectioned at 5**–**10 μm, and mounted on charged slides. After deparaffinization (for paraffin sections) or acetone fixation (for cryosections), antigen retrieval is performed using citrate buffer (pH 6.0, 95 °C, 20 min). Sections are blocked with 5 % species-matched serum (1 h, RT) and incubated with CD68 antibodies (4 °C overnight) followed by fluorophore-conjugated secondary antibodies (1 : 500, 1 h, RT, dark). Nuclei are counterstained with DAPI (1 μg/mL, 5 min) and slides mounted with antifade medium. Imaging is conducted using a confocal microscope with appropriate filter sets, with controls including no primary antibody and isotype matched IgG.

**5.6 Functional Regulation of ADF in Vitro**

*EdU Assay:* Seed keratinocytes (HaCat), fibroblasts (HDFs), human vascular endothelial cells (HUVECs), and adipose-derived stem cells (ADSCs) into a 96-well plate at a density of 5000 cells per well. Once the cells have adhered, add 0.25 % trypsin-EDTA solution and incubate for 5 minutes to digest. Then, add culture medium containing FBS to stop the digestion. EdU reagent to a working concentration of 20 μM with culture medium, and add 100 μL of the EdU working solution to each well to achieve a final concentration of 10 μM. Incubate the cells in a 37 °C incubator for an appropriate duration (approximately 2 h). After labeling is complete, remove the culture medium and wash the cells with PBS 1-2 times. Add 50 μL of 4 % paraformaldehyde to each well and fix the cells for 10-15 minutes. Remove the fixative, and wash the cells with PBS three times, each for 3-5 minutes. Add 100 μL of permeabilization solution (PBS containing 0.1 % Triton X-100) to each well and incubate at room temperature for 10-15 minutes. Follow the instructions of the EdU reaction detection kit for fluorescence labeling; nuclear dyes such as DAPI (1 μg/mL) can be used simultaneously for nuclear staining. Detect EdU-positive cells with fluorescence microscopy, calculate the proportion of these cells relative to the total cell count to assess cell proliferation.

*Transwell Assay:* Digest keratinocytes (HaCat), fibroblasts (HDFs), and adipose-derived stem cells (ADSCs), into a single cell suspension and count them. Adjust the cell density according to the experimental requirements. Add the cell suspension to the upper chamber of the Transwell, avoiding air bubbles. Simultaneously, add chemoattractant medium to the lower chamber to induce cell migration. Place the Transwell in a 37 °C, 5 % CO_2_ incubator and culture for an appropriate duration as required by the experiment. Observe cell migration and invasion in the Transwell chamber using an inverted microscope. Record the number of migrated cells and morphological changes.

*Adipogenic Differentiation Assay:* Culture adipose-derived stem cells in medium containing 10 % fetal bovine serum until they reach 80-90 % confluence. Digest the cells with trypsin-EDTA, then use medium containing 10 % fetal bovine serum to stop the digestion and resuspend the cells. Count the cells and adjust the cell density to 2.0 × 10^4^ cells/mL, seed them into a 24-well plate at 0.5 mL per well, and culture until they reach 80-90 % confluence again. Replace the medium with adipogenic differentiation medium (0.5 mL per well) and culture in a 37 °C, 5 % CO_2_ incubator for 10 to 21 days, with medium changes every 2-3 days. During the culture period, adjust the differentiation period by observing the intracellular lipid droplet formation under a microscope. Stain the lipid droplets within the cells using methods such as Oil Red O staining to assess the effectiveness of adipogenic differentiation.

*Scratch Assay:* On the bottom of a six-well plate, use a marker and a ruler to draw three horizontal lines as markers. Seed vascular endothelial cells (HUVECs) at a density of approximately 5 × 10^5^ cells per well according to the group assignment and spread evenly. After the cells have grown to confluence, use a ruler to guide two vertical lines with a 200 μL pipette tip, intersecting the marked lines to form several fixed points for observation. Discard the old culture medium and gently rinse with PBS 2-3 times until the detached cells are thoroughly washed away. Add compound-containing medium or serum-free culture medium according to the group assignment. Take photos under a microscope at 100× magnification, ensuring the scratches are centered and the background is consistent. Process the images and measure the scratch width using software ImageJ. Calculate the wound closure rate and present the results in a bar graph with time on the x-axis and healing degree on the y-axis.

*Tube Formation Assay:* Choose vascular endothelial cells (HUVECs) and use Matrigel to create a basement membrane-like gel matrix as a growth area. Thaw Matrigel from -20 °C and allow it to transition from solid to liquid at 0 °C, avoiding temperatures above 10 °C to prevent premature solidification. Unopened Matrigel® should be aliquoted to avoid repeated freeze-thaw cycles that could affect quality. Add 50 μL of Matrigel® to each well of a pre-chilled 96-well plate, gently tap to ensure an even surface, and then incubate at 37 °C for 30 minutes to 1 hour to solidify. Digest HUVECs with trypsin, centrifuge, resuspend, and count them. Resuspend cells at a density of approximately 2.0 × 10^4^ cells per 100 μL of culture medium. After the Matrigel® has solidified, gently add the cell suspension on top of the Matrigel®, being careful not to disrupt the surface. Set up three replicates for each experimental group. Place the 96-well plate with cells into the cell culture incubator for the tube formation assay. Begin observation after 4 h, capture bright-field images of tube formation directly using an image acquisition device. Analyze the images using software ImageJ.

*CCK-8 Cell Viability Assay:* Cells are seeded in 96-well plates (5 × 10³ cells/well) and incubated overnight (37 °C, 5 % CO₂) prior to treatment application. After 24, 48 and 72 h treatment exposure, 10 μL CCK-8 reagent is added per well (1 : 10 ratio with culture medium) and incubated for 1-4 h (37 °C, light-protected). Absorbance is measured at 450 nm with 650 nm reference using a microplate reader, with viability calculated as: [(ODₜᵣₑₐₜ - ODₙₑᵍ)/(ODᵤₙₜᵣₑₐₜ - ODₙₑᵍ)]×100 %. The assay includes blank control (medium-only) and positive (10 % DMSO in medium) controls (n = 3 replicates/group), with optimization of incubation time confirmed via linear correlation (R²>0.98) between cell number (1 × 10³-1 × 10⁴ cells/well) and OD450.

*LDs isolated from human adipose tissue:*

**5.7 Mass Spectrometry Analysis**

The samples of ADF and F-ADF were treated with RIPA lysis buffer and were loaded onto a 10 % SDS-PAGE gel. Following Coomassie brilliant blue staining, the gels were excised into five molecular weight fractions based on their molecular weight profiles. The gel pieces were then dehydrated with acetonitrile and rehydrated in 1 % (v/v) formic acid to prepare for enzymatic digestion with trypsin. Subsequently, the samples were loaded into a mass spectrometer (Model LTQ, Thermo Fisher Scientific, USA) for analysis. The spectrometer was meticulously calibrated with standard compounds and set to operate in a data-dependent acquisition mode. This mode facilitated an automated sequence of full mass spectrometry (MS) scans across the mass-to-charge (m/z) range of 300-2000, coupled with the targeted MS/MS scans of the ten most prevalent ions detected in each MS scan, ensuring comprehensive data collection for peptide and protein identification.

**5.8 Mass spectrometry data processing**

We implemented a rigorous, multi-stage proteomic analysis pipeline beginning with stringent quality control of mass spectrometry data, requiring protein detection across at least three biological replicates to ensure reproducibility. Initial data assessment employed multivariate analytical approaches including principal component analysis, Spearman rank correlation, and hierarchical clustering to verify technical consistency across experimental conditions prior to downstream analysis. For differential protein expression profiling, we utilized the limma package with conservative dual-threshold criteria: absolute log2-fold change ≥ 1 (corresponding to linear fold changes ≥ 2 for upregulation or ≤ 0.5 for downregulation) combined with false discovery rate (FDR) adjustment < 1 % (-log_10_[p-value] > 2). Functional characterization focused on abundant proteins through comprehensive annotation including biological process enrichment, subcellular localization mapping, and molecular function analysis. Pathway interrogation was conducted through dual complementary approaches: KEGG pathway enrichment analysis and Gene Ontology classification via the PANTHER system. Protein interaction networks were reconstructed and analyzed using the MCODE algorithm in Cytoscape (v3.9.1) with optimized parameters (Node Score Cutoff = 0.2, K-Core = 2, Max Depth = 100) to identify functionally relevant protein modules. All analytical workflows were implemented in R (v4.2.1) with appropriate multiple testing corrections, employing specialized visualization packages including ggplot2 for general plotting, UpSetR for set visualization, and Cytoscape (v3.9.1) for network representation. This integrated methodology was designed to maintain statistical rigor while enabling multi-dimensional investigation of proteomic profiles, from initial quality assessment to functional and network-level characterization.

**5.9 Retention rate evaluation (mass, volume) of the ADF**

The volume and mass of the Implanted ADF and F-ADF at different time points ( 2 weeks, 4 weeks, 12 weeks, and 24 weeks) were measured using the displacement method and a balance scale, respectively. The calculation formula for the retention ratewasas follows:


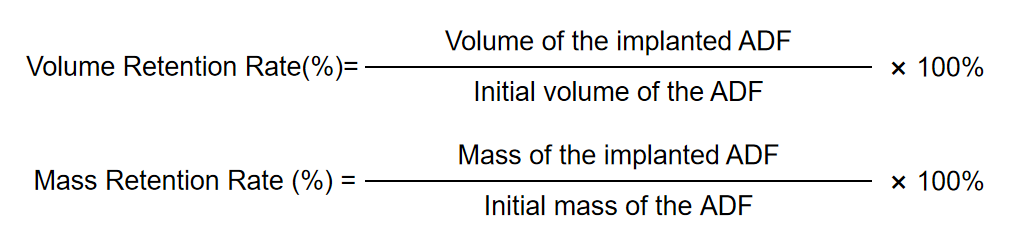


**5.10 Morphological Studies of the Implanted ADF**

A normal process mentioned above was used to obtain formalin-fixed, paraffin-embedded the implanted ADF and F-ADF slices. A standard protocol was followed for hematoxylin and eosin (HE), and Masson’s staining as mentioned above. Conventional protocols for immunofluorescence staining and immunohistochemistry assays were followed.

**5.11 Immunofluorescence Staining of the Implanted ADF**

The implanted ADF and F-ADF sections were dewaxed, followed by antigen retrieval with 10 mmol/L citrate buffer at pH 6.0. After serum blocking, the sections were incubated with the following primary antibodies: anti-perilipin 1 (ab3526, Abcam, dilution 1:500), anti-CD31 (GB11063-2, Servicebio, dilution 1:1000), anti-α-SMA (GB12045, Servicebio, dilution 1:5000), anti-F4/80 (GB113373, Servicebio, 1:1000), anti-CD11c (GB11059, Servicebio, 1:500), anti-Ly6G (GB11229, Servicebio, 1:500), anti-CD14 (GB14023-50, Servicebio, 1:500), anti-CD206 (GB113497-100, Servicebio, 1:500) and anti-TREM2 (ab305103, Abcam, dilution 1:500). These antibodies were used for detection of targeted proteins. The corresponding species-specific secondary antibodies were applied for 1 hour at room temperature.

**5.12 Immunohistochemistry Analysis of the Implanted ADF**

Immunohistochemistry was performed on the Implanted ADF and F-ADF at different time points ( 2 weeks, 4 weeks, 12 weeks, and 24 weeks). Paraffin sections were dewaxed, followed by antigen retrieval with 10 mmol/L citrate buffer at pH 6.0. Deparaffinized sections were treated with methanol containing 3 % hydrogen peroxide for 15 min. After washing with PBS, the sections were incubated with blocking serum for 30 min. Then, the sections were incubated with anti-COL1 (ab279711, Abcam, dilution 1:500) at 4 °C overnight to detect the target proteins. Hematoxylin was used to counterstain the nuclei.

**5.13 The GFP^+^ Mouse to Nude Mouse Bone Marrow Transplantation Model**

Male C57/BL6 mice (approximately 8 weeks old) were irradiated (900 cGy) and then injected via the tail vein with bone marrow isolated from age-matched GFP^+^ donor mice (1 × 10^6^ cells in 200 μL PBS). Two weeks later, GFP^+^ cells were present in the mouse circulatory system, and their presence was verified using immunofluorescence methods.

**5.14 Flow Cytometry for Peripheral Blood Leukocytes**

Fresh peripheral blood (EDTA-anticoagulated) is processed within 2 h using a modified erythrocyte lysis protocol: (1) 100 μL whole blood is mixed with 2 mL room-temperature ammonium-chloride-potassium (ACK) lysis buffer (155 mM NH4Cl, 10 mM KHCO3, 0.1 mM EDTA, pH 7.4) by gentle inversion for 5 minutes; (2) The reaction is immediately stopped by adding 10 mL ice-cold PBS/0.5 % BSA; 3) Leukocytes are pelleted (300 × g, 5 min, 4 °C) and washed twice. For immunophenotyping, the isolated leukocytes (1 × 10^6^ cells/test) undergo sequential staining: Surface marker staining with CD45-PE. Flow acquisition on calibrated instruments includes compensation controls.

**5.15 Macrophage Depletion using Clodronate liposome**

Clodronate Liposome (5 mg/mL) are administered via intraperitoneal injection (200 μL per 20-25g mouse) to achieve targeted macrophage depletion, with PBS-loaded liposomes serving as controls. For sustained depletion, repeat injections are performed every 72 h.

**5.16 Wound Healing Evaluation**

The wound healing efficacy of ADF, F-ADF, and ADM was evaluated in a full thickness wound model in nude mice (20–25 g). All procedures were approved by the Institutional Animal Care and Use Committee (No. IACUC-20240726) and conducted in compliance with ethical guidelines. Following anesthesia (isoflurane), a 15 × 15 mm full thickness wounds were created. To standardize wound induction, all procedures were performed by a single operator. Wounds were covered with 3M film to prevent contraction. Nude mice were randomized into four groups (n = 8/group): (1) untreated control, (2) ADM, (3) ADF, and (4) F-ADF. Treatments were applied topically. Wound areas were photographed on days 0, 4, 7, 10, 14, and 18 post-injury and quantified using ImageJ. On day 7 and day 14, wound tissues were harvested for histopathological (HE; Masson’s trichrome staining) and immunofluorescence analyses (CD31/α-SMA for angiogenesis; F480/TREM2 for Trem2^+^ macrophage).

**5.17 Single-Cell RNA-seq Analysis**

ScRNA-seq data were subjected to Seurat package (version 5.0)^[71]^. The preprocessing for each sample was performed as follows. Genes detected in fewer than 3 cells and those cells with expression of fewer than 200 genes were all excluded. Then, cells with > 200 genes and < 10 % mitochondrial reads were retained for downstream analysis. For the doublet detection, the R package DoubletFinder (version 2.0.3)^[72]^ was used to remove the potential doublets with default settings. Following this, the preprocessed dataset was normalized, scaled and followed by using highly variable genes for PCA. The main cell groups were identified using a resolution = 0.8. Similarly, in the major clustering analysis of all cells, the resolution value = 0.6. The data visualization was displayed using the UMAP projection with the first 20 principal components. Additionally, we utilized the FindAllMarkers function to identify the marker genes of each cluster for cell population annotation. Meanwhile, the classical markers for cell identities and lineages were also used for assigning each cluster. Finally, eleven distinct subgroups were well characterized by their unique gene markers. The clusterProfiler package with Benjamini-Hochberg multiple testing adjustment^[73]^ was employed to conduct GO-BP enrichment analysis for each macrophage subgroup. The significantly enriched Gene Ontology Biological Process (GO-BP) terms were shown based on the p < 0.05.

**5.18 Cell-Cell Chat Analysis**

Cell-cell ligand-receptor interaction analysis was conducted using CellChat (version 5.0)^[74]^. The potential communication between four macrophage subpopulations and other remaining clusters were evaluated. Briefly, the normalized counts and cell-type annotations for each cell were first imputed into CellChat to create a CellChat object. Then, the communication probability was inferred using the compute Commun Prob and compute Commun Prob Pathway function. Moreover, the potential ligand-receptor pairs were determined using default settings. In addition, the contribution of each ligand receptor pair to the signaling pathways was further calculated and filtered. Finally, selected specific ligand-receptor pairs were displayed in CellChat with default parameters.

**5.19 Western Blot**

Tissues and cells are lysed in RIPA buffer (containing 1 % protease/phosphatase inhibitors) on ice for 30 min, followed by centrifugation (12,000×g, 15 min, 4 °C) to collect supernatants. Protein concentrations are determined by BCA assay, and equal amounts (20-50 μg) are separated by SDS-PAGE (8-12 % gels) and transferred to PVDF membranes. After blocking with 5 % non-fat milk (1 h, RT), membranes are incubated with primary antibodies (diluted in TBST, 4 °C overnight) followed by HRP-conjugated secondary antibodies (1:5000, 1 h, RT). Signals are developed using ECL substrate and quantified by ImageJ, with GAPDH as loading controls.

**5.20** **qPCR for Gene Expression Analysis**

Total RNA is extracted using TRIzol® reagent, followed by DNase I treatment and reverse transcription into cDNA using oligo(dT)18/random hexamers. qPCR reactions are prepared in triplicate with SYBR Green Master Mix (10 μL), diluted cDNA (1 μL), and gene-specific primers (0.8 μL each, 10 μM) in a 20 μL final volume. Amplification is performed under standardized cycling conditions: initial denaturation at 95 °C for 10 min, followed by 40 cycles of 95 °C for 15 sec and 60 °C for 1 min, with melt curve analysis (65-95 °C) to verify amplification specificity. Data are analyzed using the ΔΔCt method with GAPDH/β-actin as reference genes, applying efficiency correction (slope -3.1 to -3.6) and excluding samples with Ct >35 or abnormal melt curves.

PPARγ

Forward: 5’-TCTGGCCCACCAACTTCGG-3’;

Reverse: 5’-GCAGCAGGTTGTCTTGGATG-3’

C/EBP-α

Forward: 5’-CAAGAACAGCAACGAGTACCG-3’

Reverse: 5’-GTCACTGGTCAACTCCAGCAC-3’

iNOS

Forward: 5’-GTTCTCAGCCCAACAATACAAGA-3’

Reverse: 5’-GTGGACGGGTCGATGTCAC-3’

Arg1

Forward: 5’-CTCCAAGCCAAAGTCCTTAGAG-3’

Reverse: 5’-AGGAGCTGTCATTAGGGACATC-3’

TNF-α

Forward: 5’-CCCTCACACTCAGATCATCTTCT-3’

Reverse: 5’-GCTACGACGTGGGCTACAG-3’

IL-10

Forward: 5’-GCTCTTACTGACTGGCATGAG-3’

Reverse: 5’-CGCAGCTCTAGGAGCATGTG-3’

**5.21** **Statistical analysis**

For comparisons between two independent groups, Student's t-tests (unpaired and two-tailed) with Welch's correction (for unequal variances) were employed. Data are reported as mean ± standard deviation (SD). Effect sizes were calculated as Cohen's d with 95 % confidence intervals (CIs). Multiple group comparisons were analyzed using one-way analysis of variance (ANOVA) with Tukey's post hoc test following confirmation of normality (Shapiro-Wilk test, p > 0.05) and homogeneity of variance (Levene's test, p>0.05). Results are presented as mean ± standard error of the mean (SEM), with effect sizes quantified using partial eta-squared. For comparisons involving two independent variables (e.g., group × time), a two-way analysis of variance (two-way ANOVA) was performed. If the design included repeated measures, sphericity was assessed using Mauchly’s test (p < 0.05); violations were corrected via the Geisser-Greenhouse method. Significant main effects or interactions were further analyzed with Sidak’s post hoc tests for multiple comparisons. Continuous data are presented as mean ± SD, with generalized eta-squared reported as the effect size metric. All statistical analyses were performed using GraphPad Prism version 8.0 (GraphPad Software, San Diego, CA, USA), with statistical significance thresholds set at *p < 0.05, **p < 0.01, and ***p < 0.001. Image-based quantifications (e.g., fluorescence intensity, wound closure area) were processed using ImageJ software (v1.53; National Institutes of Health). This standardized analytical pipeline ensures robust statistical evaluation and reproducibility across biological replicates.
